# Two ARGONAUTE proteins loaded with transposon-derived small RNAs are associated with the reproductive cell lineage in Arabidopsis

**DOI:** 10.1093/plcell/koad295

**Published:** 2023-12-07

**Authors:** Gabriele Bradamante, Vu Hoang Nguyen, Marco Incarbone, Zohar Meir, Heinrich Bente, Mattia Donà, Nicole Lettner, Ortrun Mittelsten Scheid, Ruben Gutzat

**Affiliations:** Austrian Academy of Sciences, Vienna Biocenter (VBC), Gregor Mendel Institute of Molecular Plant Biology, 1030 Vienna, Austria; Austrian Academy of Sciences, Vienna Biocenter (VBC), Gregor Mendel Institute of Molecular Plant Biology, 1030 Vienna, Austria; Austrian Academy of Sciences, Vienna Biocenter (VBC), Gregor Mendel Institute of Molecular Plant Biology, 1030 Vienna, Austria; Faculty of Mathematics and Computer Science & Department of Plant and Environmental Sciences, Weizmann Institute of Science, 7610001 Rehovot, Israel; Austrian Academy of Sciences, Vienna Biocenter (VBC), Gregor Mendel Institute of Molecular Plant Biology, 1030 Vienna, Austria; Austrian Academy of Sciences, Vienna Biocenter (VBC), Gregor Mendel Institute of Molecular Plant Biology, 1030 Vienna, Austria; Austrian Academy of Sciences, Vienna Biocenter (VBC), Gregor Mendel Institute of Molecular Plant Biology, 1030 Vienna, Austria; Austrian Academy of Sciences, Vienna Biocenter (VBC), Gregor Mendel Institute of Molecular Plant Biology, 1030 Vienna, Austria; Austrian Academy of Sciences, Vienna Biocenter (VBC), Gregor Mendel Institute of Molecular Plant Biology, 1030 Vienna, Austria

## Abstract

In sexually propagating organisms, genetic, and epigenetic mutations are evolutionarily relevant only if they occur in the germline and are hence transmitted to the next generation. In contrast to most animals, plants are considered to lack an early segregating germline, implying that somatic cells can contribute genetic information to progeny. Here we demonstrate that 2 ARGONAUTE proteins, AGO5 and AGO9, mark cells associated with sexual reproduction in Arabidopsis (*Arabidopsis thaliana*) throughout development. Both AGOs are loaded with dynamically changing small RNA populations derived from highly methylated, pericentromeric, long transposons. Sequencing of single stem cell nuclei revealed that many of these transposons are co-expressed within an AGO5/9 expression domain in the shoot apical meristem (SAM). Co-occurrence of transposon expression and specific ARGONAUTE (AGO) expression in the SAM is reminiscent of germline features in animals and supports the existence of an early segregating germline in plants. Our results open the path to investigating transposon biology and epigenome dynamics at cellular resolution in the SAM stem cell niche.

## Teaser

Transposons are expressed in a subset of SAM stem cells, processed into small RNAs, and loaded onto AGO proteins to influence DNA methylation and likely transposon activity.

**IN A NUTSHELL**

**Background:** In plants, stem cells in the shoot apical meristem (SAM) produce new organs such as leaves and flowers. Transposons are parasitic, self-replicating genetic elements, and evolutionary theory predicts that transposons are active in stem cells, allowing them to be transmitted to the next generation. Transposon activity can damage the cell's genome and, therefore, plants also deploy cellular defenses against the propagation of transposons. Among these defenses are epigenetic mechanisms involving ARGONAUTE (AGO) proteins and small RNAs, with AGO5 and AGO9 showing high abundance in SAM stem cells.

**Question:** This study delves into the roles of AGO5 and AGO9 throughout plant development, particularly their potential in safeguarding meristem cells from transposon activity.

**Findings:** Investigating *AGO5* and *AGO9* expression through various stages of plant development, the study unveils surprising dynamics within SAM stem cells, especially in the subepidermal layer, which provides the progenitors for reproductive cells. These cells also exhibit heightened activity of potentially dangerous transposons. Furthermore, these transposons are processed into small RNAs by the cell and loaded onto AGO5 and AGO9. This resembles a genomic conflict between the host genome and transposons, similar to observations in animal reproductive cells. This is also evidence for the presence of a specialized group of reproductive cells within the meristem from an early developmental stage.

**Next step:** These insights into the variability of SAM stem cells pave the way for in-depth research on transposon behavior and gene control within these cells. Future studies could extend to examining transposon control through generations, particularly under environmental stresses like elevated temperatures.

## Introduction

All postembryonic, above-ground organs of plants originate from stem cells in the center of the shoot apical meristem (SAM), marked by the expression of *CLAVATA3* (*CLV3*) in Arabidopsis (*Arabidopsis thaliana*) (Gross-Hardt and Laux 2003). Upon initiation of flowering, the vegetative SAM develops into an inflorescence meristem, which produces floral meristems. These develop floral organs including stamens and carpels that harbor male and female gametophytes. Gametophytes develop within flower organ primordia from micro- and megaspore mother cells and are derived from the subepidermal (L2) layer of the inflorescence meristem (Jenik and Irish 2000).

Whether or not plant germline cells are set apart before floral development and when germline identity is established has been debated (Lanfear 2018; Burian 2021). The developmental timing of germline segregation affects the rate of heritable mutations and determines the units of selection for a given species (Sutherland and Watkinson 1986). The germline sensu stricto (in the strict sense) describes morphologically distinct germ cells and their precursor cells (Berger and Twell 2011; Grossniklaus 2011; Burian 2021). However, the germline sensu *lato* (in the broad sense) described as the lineage of cells connecting one generation to the next, also includes the zygote and cells of the SAM. These germline cells sensu *lato* have also been referred to as germ track (Haig 2016) and are synonymous with August Weismann's *Keimbahn* (literally translating to germline) (Weismann 1893). Cells of an early segregating germ track in Arabidopsis would likely reside in L2 cells of the SAM (Jenik and Irish 2000) and should be recognizable by the expression of specific genes. Evolutionary theory also predicts the activity of transposable elements (TEs) in cells of the germ track (Haig 2016). The evolutionary success of transposons depends on their ability to proliferate in host cells that guarantee vertical transmission from parent to offspring. In somatic cells, new insertions may pose a risk to host fitness without the benefit of being propagated to subsequent generations. Therefore, transposons are subjected to selective pressure to exert their activity in cells that are part of the germline or germ track. Any potential reduction in host fitness due to transposon activity during vertical transmission can be compensated by the increased copy number of transposons.

High expression levels of TEs, as well as TE-silencing-related genes such as ARGONAUTE (AGO) genes, have indeed been found in vegetative SAM stem cells of Arabidopsis (Gutzat et al. 2020) and rice (*Oryza sativa*) (Higo et al. 2020). However, addressing the question of germ track identity in the SAM remains challenging due to the difficulty of isolating and characterizing specific cell populations from shoot meristems.

AGO proteins are components of all small RNA (sRNA)-related pathways. The Arabidopsis genome contains 10 genes in 3 clades encoding AGO proteins. The AGO1/5/10 clade is associated with post-transcriptional gene silencing (PTGS) by binding to microRNAs (miRNAs) and targeting mRNA for degradation or translational inhibition (Borges and Martienssen 2015). The AGO4/6/9 clade is associated with guiding RNA-directed DNA methylation (RdDM) to transposon sequences (Borges and Martienssen 2015). RdDM activity can be recognized by DNA methylation in the CHH context (H indicates any base but G), mainly on short TEs on chromosome arms (Kim and Zilberman 2014). Pericentromeric TEs are kept in a methylated heterochromatic state by the activity of the SWI/SNF2 chromatin remodeler DDM1 and the DNA methyltransferase CMT2 to establish CHH methylation (Zemach et al. 2013; Dubin et al. 2015). In mutants lacking DDM1, transposons and other repetitive sequences are massively transcribed (Dieguez et al. 1998; Jeddeloh et al. 1999; Hirochika et al. 2000; Singer et al. 2001; Kato et al. 2003), and binding of miRNA-loaded AGO1 to transposon transcripts triggers the synthesis of secondary 21/22 nt-long siRNAs, thereby adding a PTGS layer to transposon repression (Creasey et al. 2014). These transposon-derived siRNAs, termed epigenetically activated siRNAs (easiRNAs), have also been found in male gametes (Borges et al. 2018; Martinez et al. 2018).

We previously observed *AGO5* and *AGO9* expression in SAM stem cells (Gutzat et al. 2020) and hypothesized that they might contribute to safeguarding germline-precursor cells in the meristem from transposon invasion. Here, we characterize the spatial and temporal expression of AGO5 and AGO9 and their small RNA cargo. Both AGOs are expressed in distinct domains of the SAM, and their expression follows the developmental route to gametes and gamete companion cells. Furthermore, the specific expression patterns in vegetative meristems allowed us to determine sRNA populations of L2 stem cells. Our results suggest that L2 stem cells of vegetative SAMs permit the expression of TEs from highly heterochromatic regions while they are also equipped with additional silencing layers. Taken together, AGO5 and AGO9 are hallmarks of SAM stem cell heterogeneity, and the L2 is characterized by inflated TE expression and host counter-defense, including the easiRNA pathway.

## Results

### AGO5 and AGO9 are present along SAM stem and reproductive cells throughout development

To investigate the spatial distribution of AGO5 and AGO9 *in planta*, we generated reporter lines expressing both proteins with N-terminal GFP tags under the control of their respective promoters and in the respective mutant background.

**Figure 1.**
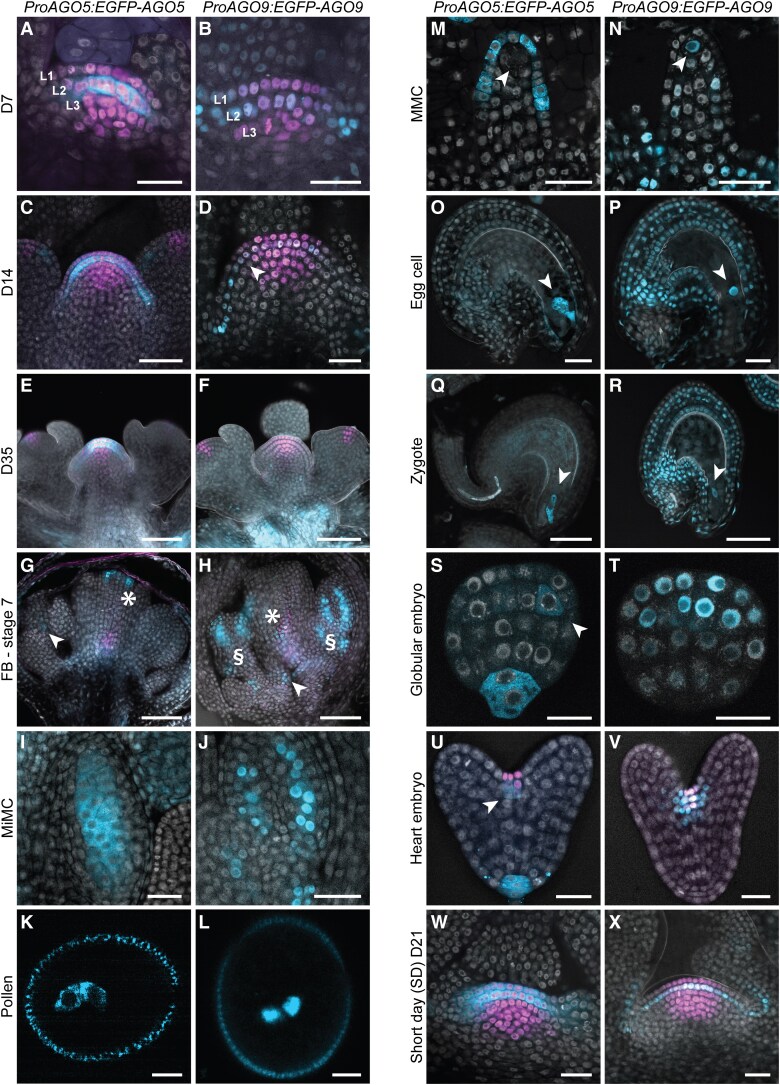
*AGO5* and *AGO9* expression throughout development. Expression of *ProAGO5:EGFP-AGO5* (in cyan, A, C, E, G, I, K, M, O, Q, S, U, W) and *ProAGO9:EGFP-AGO9* (in cyan, B, D, F, H, J, L, N, P, R, T, V, X) in plant lines containing the stem cell reporter *ProCLV3:H2B- mCherry* (red) and lacking endogenous *AGO5* or *AGO9*. **A, B)** Meristems of 7-day-old seedlings. **Cc, Dd)** Meristems of 14-d-old seedlings. **E, F)** Inflorescence meristems of 35-day-old plants. **G)** FB in stage 7; asterisk marks the upper region of the gynecium; arrowheads the inner layers of future anthers. **H)** as **G)** Arrowhead marks the base of the bud; asterisk the gynecium (asterisk); section signs (§) the inner layers of future stamens. **I, J)** Microspore mother cells in developing anthers. **K, L)** Mature pollen. **M, N)** megaspore mother cells (arrowhead) in developing ovules. **O, P)** Egg cells (arrowhead) within embryo sacs. **Q, R)** Zygotes (arrowhead). **S, T)** Embryos at the globular stage. **U, V)** Embryos at the heart stage. **W, X)** Meristem of 21-d-old plants grown under a short daylight regime. Scale bars k, l = 5 *μ*m; A, B, D, I, J, M, N, S, T, U, V, W, X = 20 *μ*m; C, E, F, G, H, O, P, Q, R = 50 *μ*m.

*ProAGO5:EGFP-AGO5* yielded a specific signal in the cytoplasm of stem cells in the L2 of seedlings 7 days after germination (D7) (Fig. 1A; Supplemental Figs. S1 to S3A). During development, AGO5 also localized to the L1 (Fig. 1, E and W; Supplemental Fig. S3, E and G) and was visible in axillary meristems (Supplemental Fig. S4A). Throughout flower development, AGO5 was initially seen in the L1 of developing carpels (Fig. 1, G and M; Supplemental Fig. S3, I, K, S and U), male meiocytes (Fig. 1, G and I; Supplemental Fig. S3, K and M), and eventually in egg and sperm cells of mature gametophytes, in agreement with previous reports (Borges et al. 2011; Sprunck et al. 2019) (Fig. 1, K and O) but was absent in microspores (Supplemental Fig. S3O). AGO5 signal was also present in the zygote (Fig. 1Q; Supplemental Fig. S3Y) and during embryo development, up to the octant stage, uniformly distributed in the embryo proper (Supplemental Fig. S3, A′ and C′). In the globular stage, AGO5 appeared to be restricted to the SAM L2, hypophysis, and organizer (Fig. 1S; Supplemental Fig. S3E′), in the heart and torpedo stage to L2 and L3 of the SAM and the root apical meristem (RAM) (Fig. 1U; Supplemental Fig. S3, G′ and I′), in agreement with (Tucker et al. 2012).

*ProAGO9:EGFP-AGO9* localized to nuclei, mainly of the L2 in SAMs, until floral induction (Fig. 1, B and D; Supplemental Figs. S1 to S3, B, D and F). AGO9-labeled nuclei were also visible along the adaxial side of leaf petioles, apparently connecting to developing axillary meristems, where AGO9 was found at later time points (Supplemental Figs. S3F and S4B). In plants grown under a long daylight regime (causing early flower induction), AGO9 was not found in plants older than 20 d.a.g. (D21 + D35) (Supplemental Figs. S5 and S6A) but was present in D21 plants grown in a short-day regime, before flower induction, where it was restricted to the L2 (Fig. 1X, Supplemental Fig. S6B). At the onset of flowering, the AGO9 signal relocated from the inflorescence meristem into floral meristems (Fig. 1F; Supplemental Fig. S, 3F and H; Supplemental Fig. S6, C to E), initially between the whorls of carpels and stamens (Supplemental Fig. S3J). It was later found along the female and male lineages (Fig. 1, H, J and N; Supplemental Fig. S3, N, P, T and V). Like AGO5, AGO9 was present in egg and sperm cells of mature gametophytes (Sprunck et al. 2019; Jullien et al. 2022) (Fig. 1, L and P; Supplemental Fig. S3, R and X), in the zygote (Fig. 1R), and in all nuclei of early embryos (Supplemental Fig. S3, B′ and D′). After the octant stage, it gradually became more restricted to the SAM region (Fig. 1, T and V; Supplemental Fig. S3, F′, H′ and J′), where it has been observed previously (Parent et al. 2021). These localization data show that AGO9 is continuously present in the nuclei of germ cells or their precursors throughout plant development, with the exception of mature inflorescence meristems.

As the gametophytes develop from meristematic L2 cells (Jenik and Irish 2000), AGO9 labels the progenitors of the reproductive cell lineage. Although mostly cytoplasmic, AGO5 labels germ or meristematic L2 cells throughout most of development in a pattern very similar to that of AGO9 (Supplemental Fig. S3). The cytoplasmic and nuclear preference for AGO5 and AGO9, respectively, suggests that both AGOs might play complementary roles in PTGS and transcriptional gene silencing (TGS). We excluded the notion that the localization was influenced by the fixing procedure (Supplemental Fig. S7). We also tested whether the environment could influence their localization by subjecting seedlings to 24 h of severe heat stress at 37 °C. This did not change the localization patterns (Supplemental Fig. S8), confirming that the localization of AGO5 and AGO9 in the meristem is stable at different temperatures.

Neither the *ago5* nor *ago9* mutants have easily scorable phenotypes that would allow us to confirm the functionality of the tagged reporter lines by complementation. However, *ago9* was reported to have an increased number of enlarged subepidermal cells in ovule primordia—the likely precursors of megaspore mother cells (MMCs) (Olmedo-Monfil et al. 2010). We asked whether *ProAGO9:EGFP-AGO9* would complement this developmental defect. Unexpectedly, we could not observe the described difference between wild type and *ago9* mutant plants, possibly due to differences in growth conditions, as the number of enlarged subepidermal cells was also relatively high in the wild type (Supplemental Fig. S9). However, we detected a significantly increased number of enlarged subepidermal cells in *ago5 ago9* double mutants (Supplemental Fig. S9). This phenotype could be rescued by introducing either *ProAGO5:EGFP-AGO5* or *ProAGO9:EGFP-AGO9* (Supplemental Fig. S9), demonstrating that both tagged proteins are functional. This further supports the hypothesis that AGO5 and AGO9 have partially redundant functions, in this case, to restrict the number of MMC precursors in ovule primordia. We also asked whether *ago5* and *ago9* could influence stem cell number. However, analysis of D7 SAMs of *ago5*, *ago9*, and *ago5 ago9* revealed no significant differences from the control (Supplemental Fig. S10).

### The sRNA cargo of AGO5 and AGO9 is dynamic and derived from transposons

**Figure 2.**
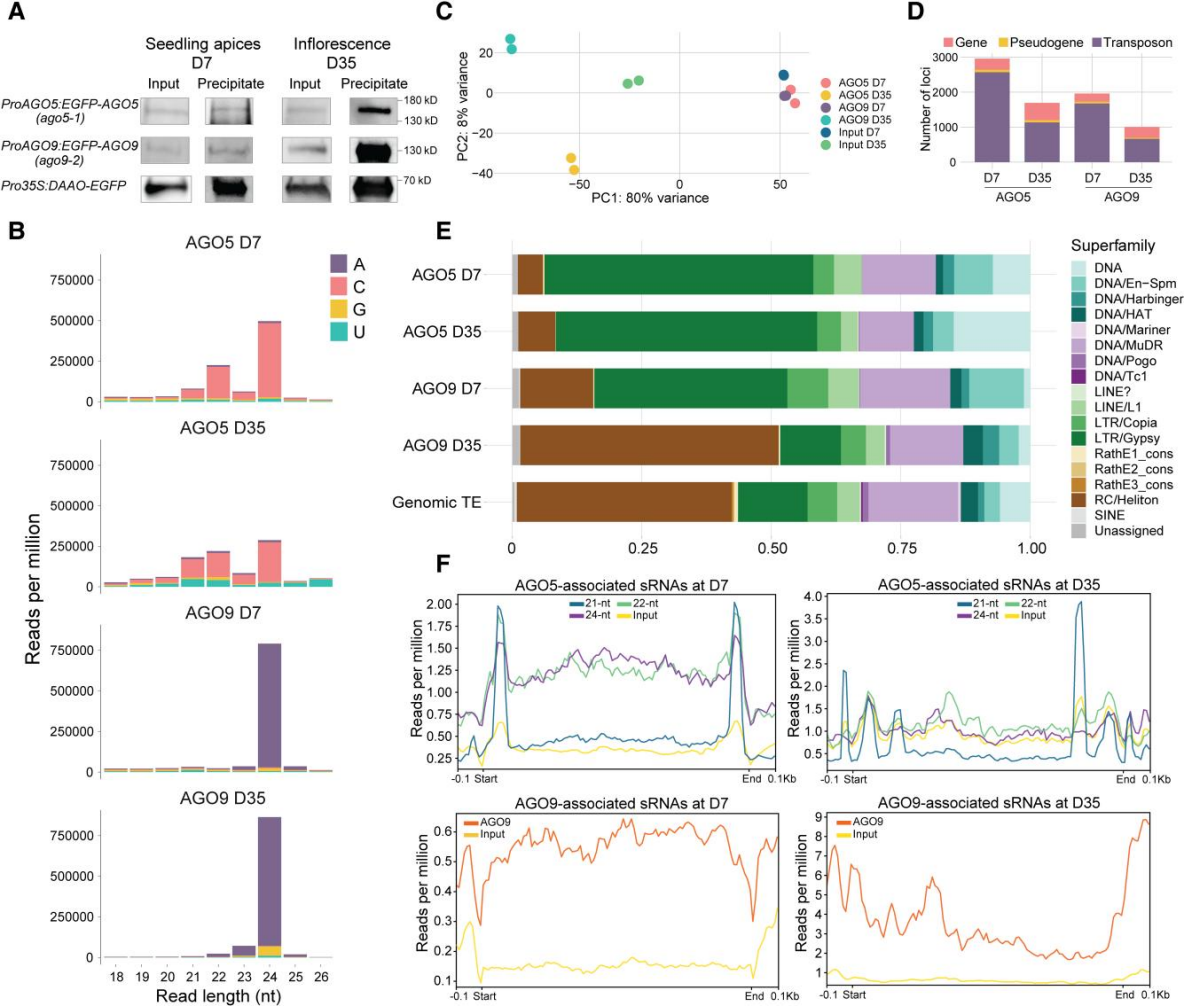
AGO5 and AGO9 sRNA cargo derived from transposons changes dynamically throughout development. **A)** Immunoblot probed with anti-GFP primary antibody after immunoprecipitation of GFP-tagged AGO5 and AGO9 from apices of 7-d-old seedlings and inflorescences from 35-d-old plants. A plant line constitutively expressing GFP-tagged DAAO served as a positive control (see Supplemental Fig. S11). **B)** Read length distribution and 5′ bias of AGO5- and AGO9-associated sRNAs. **C)** Principal component analysis of the sRNA sequencing data. **D)** Number of features enriched among AGO5 and AGO9 sRNA cargo compared to input (Wald test, fdr < 0.05, log2-fold change >1). **E)** Potential transposon targets classified by superfamily. **F)** Metaplots of sRNA distribution across the potential transposon targets. Experiments were done in biological duplicates (n = 2); however, b shows the size distribution only for 1 replicate (see Supplemental Fig. S12).

To explore the putative functions of AGO5 and AGO9 in Arabidopsis SAM stem cells and to assess sRNA pools from L2 SAM stem cells, we isolated and sequenced AGO5- and AGO9-bound sRNAs at 2 developmental time points (Fig. 2A, Supplemental Fig. S11). We chose shoot apices of D7 seedlings because of the specific expression of AGO5 and AGO9 in L2 and L1/L2 (Fig. 1). To investigate changes in AGO loading during germline differentiation, we also chose dissected apices from mature plants (D35) encompassing the inflorescence meristem, floral meristems, and very young flower buds (FB). Protein levels of AGO5 were low compared to the strongly accumulated AGO1, which resulted in residual AGO1 in the AGO5 precipitate (Supplemental Fig. S11). To avoid this contamination during D7 AGO5 precipitation, we depleted AGO1 by immunoprecipitation before precipitating AGO5 (Supplemental Fig. S11A). The preferential AGO5 cargo consisted of 21, 22, and 24 nt sRNAs with a 5′ C bias (Fig. 2B, Supplemental Fig. S12), as previously reported for cell cultures (Mi et al. 2008). In agreement with (Havecker et al. 2010), AGO9 was loaded mainly with 5′ A-biased 24 nt sRNAs (Fig. 2B, Supplemental Fig. S12). Principal component analysis showed an increased variance of sRNA populations at D35 (Fig. 2C), suggesting diversification of sRNA populations in AGO5 and AGO9 during later development.

A comparison between AGO-bound sRNAs with total (input) sRNAs (FDR <0.05, log2-fold change >1, Wald test) revealed that both AGOs were preferentially loaded with sRNAs derived from transposons (Fig. 2D). AGO5 was predominantly associated with LTR/Gypsy retrotransposon-derived sRNAs, whereas AGO9 cargo contained proportionally more RC/Helitron sequences (Fig. 2E, Supplemental Fig. S13, Supplemental Data Set 1). The overlap between TEs complementary to AGO5- and AGO9 cargo was highly significant but less pronounced at D35 due to the strong bias toward Helitron-derived sequences in AGO9 (Fig. 2E, Supplemental Fig. S13). Notably, TEs represented in AGO5-sRNAs were pericentromeric, but the origin of AGO9-sRNAs shifted from pericentromeres to chromosome arms during development (Supplemental Fig. S14).

AGO5-bound 21/22 nt sRNAs were mainly derived from LTR/Gypsy elements, similar to emerging easiRNAs in mutants lacking the chromatin remodeler DDM1 (Creasey et al. 2014) and must derive from the L2 of D7 seedlings. The AGO5 21 nt cargo mapped most prominently to the 3′ and 5′ end of TEs (Fig. 2F), similar to the profiles of easiRNAs in pollen (Martinez et al. 2018). AGO5-associated 22/24 nt sRNAs were distributed more uniformly along TEs, as were AGO9-bound 24 nt sRNAs (Fig. 2F). The preferential loading of TE-related sRNAs implies that both AGO5 and AGO9 are TE-silencing factors in Arabidopsis SAM stem cells throughout development.

### *AGO5*- and *AGO9*-expressing cells show high expression of TEs

TE-derived siRNAs do not necessarily act in a cell-autonomous manner. A model for the male germline proposes that TEs are expressed in companion cells and that TE-derived siRNAs migrate to gametes to reinforce RNA-directed silencing (Slotkin et al. 2009; Ibarra et al. 2012; Long et al. 2021). Therefore, we wanted to understand whether this is similar in SAMs and asked if the observed increase in TE expression in SAM stem cells (Gutzat et al. 2020) is confined to L2 cells (analogous to future gametes), to stem cells surrounding the L2 (analogous to companion cells), or is uniform across all stem cells.

**Figure 3.**
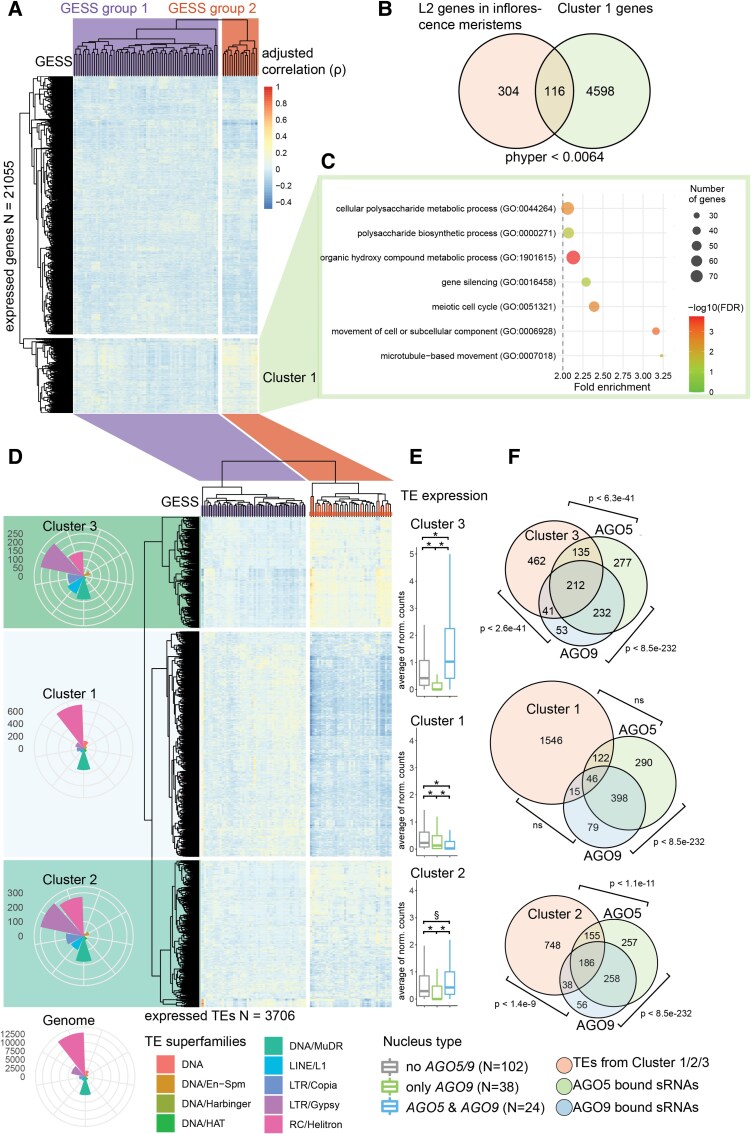
*AGO5*- and *AGO9*-expressing cells show high expression of TEs. **A)** Clustering of gene expression correlations with 79 genes expressed specifically in stem cells of 7-d-old seedlings (GESS, see text: These genes are specifically expressed in stem cells at this developmental stage). **B)** The overlap between cluster 1 genes (from our analysis) and genes expressed in the L2 layer according to a different study (Yadav et al. 2014) is shown, indicating common gene expression patterns between the 2 datasets. (Yadav et al. 2014). **C)** The most significantly enriched GO terms associated with genes in cluster 1 are displayed, providing insights into the biological processes associated with these genes. **D)** Clustering of TE expression correlation with GESS. Polar plots show the proportion of TE superfamilies and the number of TEs per superfamily in each cluster. **E)** Boxplots illustrate the expression levels of TEs in each cluster specifically in AGO5- and AGO9-containing nuclei. Box plots represent the median, upper and lower quartiles, and 1.5× interquartile range. **F)** The overlap between expressed TEs in the 3 clusters and the AGO5 and AGO9 cargo. The Venn diagram indicates shared TEs between the clusters and AGO5/AGO9 cargo. Venn diagram areas are not drawn proportionally. ρ = Spearman correlation, p = phyper, § = U-test <1e-6, * = U-test <2e-16.

To test this, we FACS-sorted and analyzed the transcriptomes of 188 individual *ProCLV3:H2B-mCherry* nuclei derived from D7 plants using SMART-seq. We found 21,055 genes and 3,706 TEs expressed in at least 4 nuclei (median of 3,197 expressed genes and TEs per nucleus, Supplemental Fig. S15A). The expression of 3 cell cycle reporters (*HTR13*, *CDT1A*, and *CYCB1.1* (Desvoyes et al. 2020)) assigned a cell cycle state to most nuclei (Supplemental Fig. S15B). To detect robust gene expression heterogeneity within this sparse dataset, we first adjusted for correlation between any 2 genes based on their total sampling (Meir et al. 2020). Then we extracted the correlations for the 3 cell cycle genes and 79 genes we previously identified with specifically high expression in D7 stem cells (Gutzat et al. 2020). We reasoned that these genes, referred to as **G**enes **E**xpressed **S**pecifically in D7 **S**tem cells (GESS) hereafter, could contain spatial information. Indeed, clustering of the adjusted correlations identified 2 major clusters, separating GESS into 2 groups (Fig. 3A). Besides *AGO5,* GESS group 2 comprises *MCT2* and *PHDG4,* 2 indicators of the L2 layer (Yadav et al. 2014), and *CDT1A,* labeling cells in the G1 phase of the cell cycle (Fig. 3A). Notably, we found cluster 1 enriched for genes involved in the meiotic cell cycle, gene silencing, and microtubule-associated genes, and a significant overlap with genes expressed in L2 cells in the inflorescence meristems (Yadav et al. 2014) (Fig. 3, B and C). These data suggest that L2 cells of the SAM stem cell niche already have a distinct expression pattern early during vegetative development and are mainly in the G1 state of the cell cycle.

Performing the same correlation analysis between GESS and TE transcripts revealed 3 main TE clusters, separating 2 groups of GESS (Fig. 3D). All GESS from group 2 in the gene cluster were present in GESS group 2 in the TE cluster, suggesting that the TE expression pattern in GESS group 2 is mainly determined by L2 nuclei (Fig. 3D). Most TEs present in cluster 1 consist of RC/Helitrons and DNA/MuDR TEs, and their relative abundance resembles their genome-wide distribution (Fig. 3D). By contrast, TE clusters 2 and 3 are strongly enriched for LTR/Gypsy transposons. Importantly, we found that sRNA cargoes loaded onto AGO5 and AGO9 are mainly derived from TEs expressed in clusters 2 and 3. (Fig. 3F).

To confirm the results of correlation analysis, we analyzed the expression of TEs in nuclei grouped into those expressing both *AGO5* and *AGO9* (representing L2 nuclei), *AGO9* only (representing L1 nuclei), or neither *AGO5* nor *AGO9*. We only used nuclei with at least 2 reads of either *AGO5*, *AGO9,* or both for this analysis. TEs from clusters 2 and 3 showed significantly increased expression in *AGO5* & *AGO9* compared to *AGO9* only or no *AGO5/9* nuclei (Fig. 3E). This demonstrates that TEs with the highest expression levels in our dataset mainly come from L2 nuclei expressing *AGO5* and *AGO9* and are also the templates for sRNAs loaded onto AGO5 and AGO9.

We also analyzed the length of the TEs present in the single nuclei RNA-seq data and found that TEs with complementary AGO5 and AGO9 sRNA cargo are significantly larger than the genomic average (Supplemental Fig. S16), suggesting that these TEs are more likely still functional copies.

We also tested whether we could directly sort L2 nuclei from AGO9-GFP labeled meristems, as the AGO9-GFP signal exhibited strict localization to the L2 layer in 21-d-old short day-grown plants and persisted for several days after floral induction by shifting plants to long days (Supplemental Fig. S17). We measured mRNA expression of L2 stem cell nuclei, L2 nonstem cell nuclei, non-L2 stem cell nuclei, and meristematic cell nuclei before and after floral induction (Supplemental Fig. S17, A and B). The purity of the cell nuclei populations was confirmed by the expression of *CLV3*, *AGO5,* and *AGO9* (Supplemental Fig. S17C). This revealed many intriguing differences in gene expression in the L2 before and after flower induction, particularly regarding cell cycle genes (Supplemental Data Sets 2 and 3). Besides *AGO5* and *AGO9*, *AGO1*, *AGO10*, and *AGO4* also showed high, but not specific expression in the L2 (Supplemental Fig. S18).

Additionally, we observed a decrease in TE expression in the L2 of 21 d-old seedlings, followed by an increase in expression after floral induction (Supplemental Fig. S17D). This dynamic TE expression pattern in the meristem throughout development aligns with our previous work (Gutzat et al. 2020) and suggests potential cycles of TE repression and derepression, possibly related to developmental transitions. In summary, the data reveal the existence of distinct niches of TE expression in SAM stem cells.

Notably, there was high expression of pericentromeric LTR/Gypsy elements in *AGO5*-expressing cells, indicating cell-autonomous synthesis of TE-derived sRNAs in 7-d-old seedlings.

### AGO5 contributes to methylation of CMT2-targeted TEs

**Figure 4.**
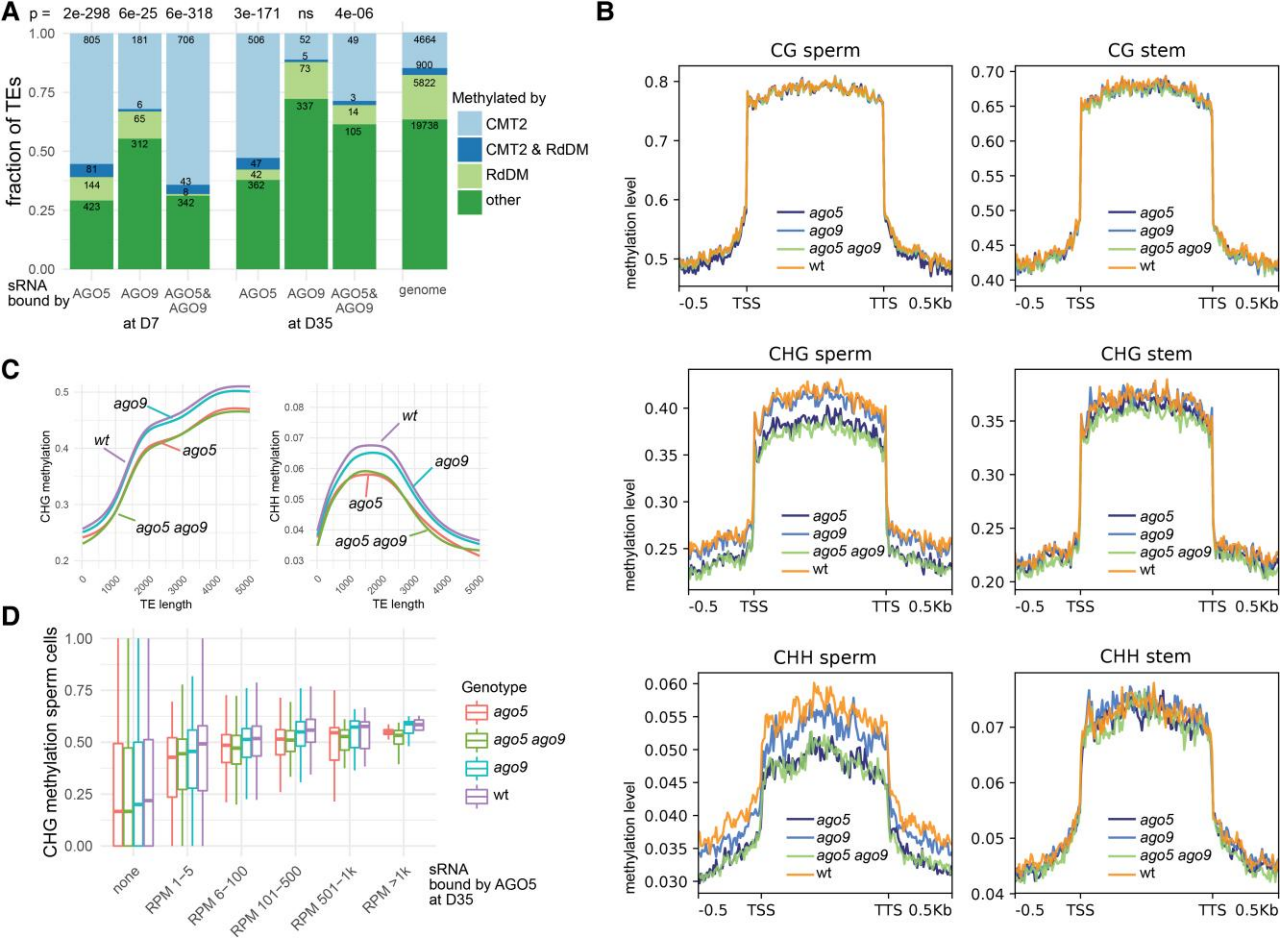
AGO5 contributes to DNA methylation of heterochromatic TEs in SAM stem and sperm cells. **A)** DNA methylation pathways of transposons (TEs) targeted by AGO5- or AGO9-associated sRNAs at an early (D7) or late (D35) developmental stage as identified by (Stroud et al. 2013) in somatic (leaf) tissue. The numbers above the bar plots indicate *P*-values (phyper) for the enrichment of transposons methylated by CMT2. Numbers in the bar plots indicate the number of transposons. **B)** Metaplots of CG, CHG, and CHH methylation at TEs in sperm and stem cells in WT, *ago5*, *ago9*, and *ago5 ago9*. **C)** CHG and CHH methylation over TE length in WT, *ago5*, *ago9*, and *ago5 ago9*. **D)** CHG methylation in sperm cells of TEs corresponding to AGO5 cargo and sorted by the abundance of AGO5-associated sRNAs. Box plots represent the median, upper, and lower quartiles and 1.5× interquartile range. RdDM, RNA directed DNA methylation; RPM, Reads per Million; TSS, Transcription Start Site; TTS, Transcription Termination Site.

Our results raised the question of whether AGO5 and AGO9 activity is important for maintaining the heterochromatic state of strongly methylated pericentromeric TEs. DNA methylation is highly dynamic in the male germline during differentiation (Walker et al. 2018) and in stem cells, which display an increase in CHG methylation and a decrease in CHH methylation during development (Gutzat et al. 2020). This indicates that the affected TEs are methylated by CMT2, as loss of this methyltransferase leads to reduced CHG and CHH methylation, especially on long heterochromatic TEs (Zemach et al. 2013). Intriguingly, we found that AGO5- and AGO9-associated sRNAs were highly enriched for sequences matching TEs methylated by CMT2 rather than the RdDM pathway (Fig. 4A) (Stroud et al. 2013; Kawakatsu et al. 2016; Papareddy et al. 2020). This could indicate an unusual AGO5- or AGO9-mediated contribution to DNA methylation at these loci in stem cells or cells of the germline by CMT2 (Stroud et al. 2013). To test this at different stages during plant and gamete development, we performed DNA methylation analysis on stem cells and male germ cells by sorting and collecting D7 stem cells and sperm nuclei of wild type (wt), *ago5, ago9,* and *ago5 ago9*. We observed a slight reduction of CHG and CHH methylation at TEs in stem cells of *ago5* and *ago5 ago9* seedlings; however, this reduction was more pronounced in sperm cells (Fig. 4B). This, surprisingly, shows that AGO5, and not the nuclear AGO9, contributes to CHG and CHH methylation. CHG and CHH methylation in sperm cells of *ago5* and *ago5 ago9* was especially reduced on TEs longer than 1000 bp (Fig. 4C). Total methylation levels of TEs matching AGO5 and AGO9 cargo were significantly higher than at other TEs (Supplemental Fig. S19), and CHG methylation levels were higher on TEs corresponding to the most abundant AGO5/9-bound sRNAs (Fig. 4D). As AGO9 is a nuclear protein, it was unexpected that *ago9* single mutants only showed a slight reduction of CHH methylation at TEs in sperm cells, with minimal additional effects observed in the double mutant (Fig. 4B).

The observed influence of AGO5 on DNA methylation could either be indirect (by repressing the mRNA of genes important for DNA methylation in the cytoplasm) or direct (by the shuttling of AGO5 with its cargo into the nucleus). AGO5 shares high sequence similarity, including a nuclear export signal at the N-terminus (NES), with AGO1, for which nuclear shuttling has been demonstrated (Bologna et al. 2018; Liu et al. 2018). To test whether AGO5, like AGO1, uses this potential NES sequence for nuclear shuttling, we mutated the AGO5 NES and transformed the resulting *ProAGO5:Clover-AGO5-NESm* into the *ago5* mutant. We observed significant accumulation of nuclear AGO5-NESm in stem and sperm cells of several independent lines (Supplemental Fig. S20). This shows that AGO5 could directly reinforce DNA methylation in the CHH and CHG context in SAM stem cells, but especially in sperm heterochromatin.

### TEs corresponding to AGO5 and AGO9 cargo are derepressed when DNA methylation is impaired

To address whether the loss of AGO5 and AGO9 results in increased transcription of the TEs corresponding to their cargo, we sequenced mRNA of D7 stem cells and nonstem cells of the SAM and sperm and vegetative nuclei of pollen from wt, *ago5, ago9,* and *ago5 ago9*. Expression of marker genes for the respective cell types (*CLV3, mCherry*, *DUO1*, *MGH3*, *VCK1)* confirmed high cell-specific enrichment (Supplemental Fig. S21B). Except for *AGO3* and *AGO8,* all *AGO* genes were expressed in D7 stem cells. By contrast, *AGO5* and *AGO9* were the only *AGO* family members for which we could detect transcripts in sperm cell nuclei (Supplemental Fig. S21C), suggesting that these 2 AGOs have nonredundant functions in sperm cells. However, the nuclear transcriptome of sperm and vegetative cells differed only minimally between the different genotypes, and only 6 TEs showed increased expression in *ago5 ago9* sperm nuclei (Supplemental Fig. S21A). This demonstrates that AGO proteins do not contribute to maintaining TE transcriptional silencing in sperm cells. Alternatively, perhaps AGO proteins other than AGO5 and AGO9 are essential for transcriptional TE silencing in sperm cells without being transcribed there but are carried over from the microspore precursor cells.

Among the AGO4/6/9 clade of AGO proteins, AGO4 is crucial for TGS in seedlings (Stroud et al. 2013) and is highly expressed in stem cells (Supplemental Figs. S18 and S21C). To probe for redundancy of AGO9 with AGO4 in vegetative meristems, we created additional multiple mutants and sequenced mRNA from shoot apices of D7 seedlings of wt, *ago4*, *ago5*, *ago9, ago4 ago9, ago5 ago9,* and *ago4 ago5 ago9*. To investigate a potential connection to the easiRNA pathway, we included *ddm1,* which is characterized by a global loss of DNA methylation, strong derepression of long and heterochromatic transposons, and emergence of easiRNAs (Creasey et al. 2014).

**Figure 5.**
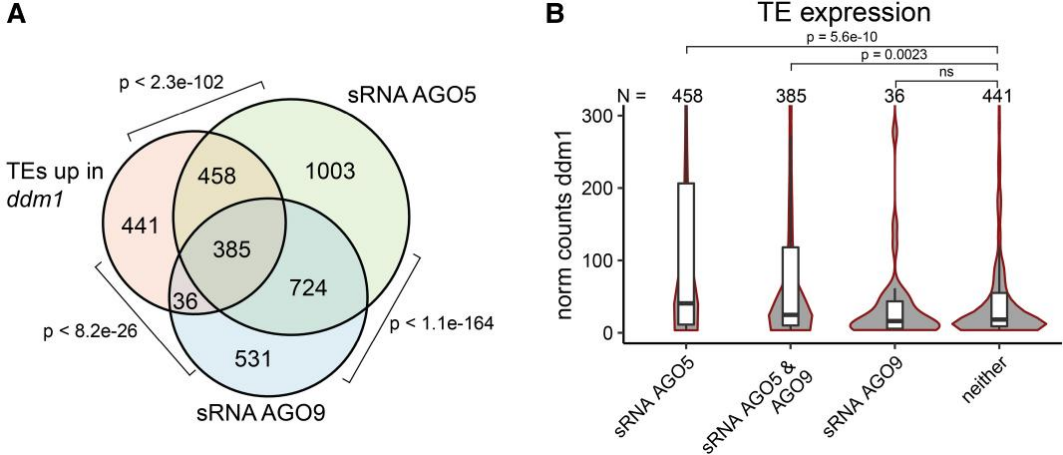
AGO5 is an easiRNA pathway effector. **A)** Overlap between AGO5 and AGO9 cargo with transposons (TEs) expressed in *ddm1*. p = phyper. **B)** Box plots showing expression levels of TEs in *ddm1*, which are either represented among AGO5 and AGO9 cargo or not. Box plots represent the median, upper, and lower quartiles and 1.5× interquartile range. p = Welch’s t-test.

Comparisons between shoot apex transcriptomes from the a*go* mutants revealed only a few differentially expressed genes and TEs (DEGs and DETs, Supplemental Fig. S22A), and only 13 TEs were upregulated in *ago4 ago9* (Supplemental Fig. S22B). By contrast, 1,320 TEs were derepressed in *ddm1* compared to wt (Supplemental Fig. S22). Interestingly, TEs upregulated in *ddm1* displayed a highly significant overlap with those specified by the AGO5 and AGO9 cargo (Fig. 5A). Furthermore, these overlapping TEs were more highly expressed in *ddm1* than those not represented among AGO5-/AGO9-associated small RNAs (Fig. 5B). Hence, TEs that are precursors of AGO5- and AGO9-associated sRNAs—and potentially targeted by these AGOs—react most strongly to the loss of DNA methylation in *ddm1*. DDM1 is a chromatin remodeler that prevents transposon activity through the deposition of histone H2A.W variants (Osakabe et al. 2021). Interestingly, we detected the reduced abundance of H2A.W6 and H2A.W7, characteristic of heterochromatin (Yelagandula et al. 2014), in the L2 of D7 seedlings relative to L1 and L3 (Supplemental Fig. S23, E to J). This reduction might contribute to the observed increase in TE expression. By contrast, the DDM1-GFP signal (Slotkin et al. 2009) was abundant in all layers of the meristem (Supplemental Fig. S23, C and D), suggesting differential post-translational regulation of DDM1 in the L2.

Furthermore, our data show that DDM1-silenced, long pericentromeric TEs are expressed in AGO5- and AGO9- containing stem cells and that siRNAs derived from these TEs are incorporated into AGO5 and AGO9. Subsequently, cells expressing AGO5 must allow either PolII or PolIV access to pericentromeres to generate the precursors.

21 and 22 nt long sRNAs bound to AGO5 resemble easiRNAs from *ddm1* or pollen. easiRNA synthesis in *ddm1* is initiated by the activity of miRNAs. We found that miRNA845, a crucial trigger of easiRNA biosynthesis (Borges et al. 2018), and other potentially TE-targeting miRNAs were significantly associated with AGO5 in SAM stem cells (Supplemental Data Set 4). The association of miRNA845 with AGO5 has also been described for pollen (Oliver et al. 2022).

The synthesis of easiRNAs in *ddm1* further depends on RNA-dependent RNA polymerase 6 (RDR6), but data from pollen suggest that easiRNAs can also be produced from POLIV transcripts (Creasey et al. 2014; Martinez et al. 2018). Therefore, we wanted to understand which of the 2 alternative pathways synthesizing TE-derived sRNAs is active in shoot apices. To this end, we used RNA gel blot analysis to probe for sRNAs of TEs that could be targeted by AGO5-bound sRNAs. Of all the mutants that we tested, *dcl2 dcl3 dcl4*, *polIV,* and *rdr1 rdr2 rdr6* showed an almost complete loss of sRNA signal (Supplemental Fig. S24). These results indicate that the generation of heterochromatic, AGO5-associated sRNAs depends on DCL2-4, POLIV, and RDR2 (Supplemental Fig. S24), which is similar to the situation in pollen and embryos (Papareddy et al. 2020).

## Discussion

Our study presents evidence for SAM stem cell heterogeneity in the young vegetative SAM. Here, we determined that the L2 is characterized by high expression of TEs, genes connected with host-counter defense, and genes with an assigned role later in meiosis, as defined by Gene Ontology (GO) analysis. We show that at any developmental time, at least 1 of the 2 AGOs, *AGO5* and *AGO9* are expressed in the cell lineage, which potentially contributes to sexual reproduction.

The change in spatial gene expression within the meristem during development, and especially at the onset of flowering, might indicate additional developmental functions of AGO5 and AGO9 that are not obvious under standard growth conditions. Alternatively, the altered spatial gene expression could also be a consequence of a developmental transition or a change in TE expression.

Co-expression analysis of single stem cell nuclei revealed 2 stem cell niches in young vegetative meristems displaying an increased expression of transposons in *AGO5-* and *AGO9-*expressing cells. Furthermore, we showed that sRNAs derived from these TEs are loaded into AGO5 and AGO9 and likely either help prevent TE mobilization by reinforcing DNA methylation at CHG and CHH sites or by PTGS in the case of AGO5; this effect is not mutually exclusive from the involvement of these proteins in de novo silencing.

Interestingly, AGO5 also binds to miRNA845 in seedlings, a crucial trigger of easiRNA biogenesis necessary for silencing hundreds of LTR/Copia and LTR/Gypsy elements in *ddm1* (Creasey et al. 2014). We could not detect increased TE activity and mobilization in *ago5*, *ago9*, or *ago5 ago9* mutants, but this is likely due to redundancy with other AGO proteins. It is also possible that AGO5 and AGO9 prevent rare instances of TE mobilization, which would be challenging to detect in short-term experiments but crucially important at the population level or from a long-term perspective. Indeed, AGO9 polymorphisms correlated with CHH methylation on TEs in epigenome association studies (Kawakatsu et al. 2016), although we detected only minimal effects of *ago9* on DNA methylation. If AGO9 is necessary for de novo DNA methylation, or if it contributes indirectly to variation of DNA methylation by post-transcriptional silencing, changes in DNA methylation in *ago9* mutants might not be detectable or might be detectable only beyond the time frame of our experiments.

Furthermore, we found that many TEs with corresponding sRNAs in AGO5 and AGO9 showed high expression in *ddm1,* although we observed increased levels of *DDM1* transcript in bulk stem cells (Gutzat et al. 2020). This suggests a chromatin state permissive for TE expression in AGO5- and AGO9-containing stem cells, similar to the chromatin state in *ddm1*. Furthermore, we found reduced levels of histone variants associated with heterochromatin and known to be incorporated by DDM1, providing additional support for this hypothesis.

To date, comprehensive genome-wide studies investigating chromatin structure with high spatial resolution in SAM stem cells are lacking. Nevertheless, previous research has hinted at the presence of specialized chromatin states in meristematic cells (You et al. 2017), which could also play a role in TE regulation. Additionally, it is conceivable that populations of TEs could be activated by stem cell-specific transcription factors or signaling networks, similar to the heat-inducible COPIA78 elements, which capture heat-responsive elements in their LTR regions (Cavrak et al. 2014).

In contrast to AGO1, which has a high affinity for 5`U-containing sRNAs (Mi et al. 2008), AGO5 has a bias for 5`C sRNAs, which probably prevents competition with AGO1 for sRNA duplexes. This suggests a functional specialization of AGO5 for post-transcriptional TE silencing via easiRNAs in the Arabidopsis germ track. However, and as discussed above, we did not find strong derepression of TEs in the absence of AGO5 or AGO9, likely because other AGOs can still trigger easiRNA biosynthesis.

Interestingly, the AGO1/5/10 clade is expanded in several plants (Zhang et al. 2015). AGO10 plays a crucial role in the development of the SAM, specifically in the Arabidopsis Ler accession, but its significance is not observed in other accessions. In Ler, AGO10 is involved in sequestering miRNAs belonging to the miR166/165 clade, which in turn regulates the expression of genes encoding development-related transcription factors of the HD-ZIP III gene family (Zhu et al. 2011; Zhou et al. 2015). AGO6 is important for de novo TGS, and intriguingly, its primary functions have been reported in the shoot and root meristems (Eun et al. 2011; McCue et al. 2015). A role in flowering time regulation has been reported for AGO5 (Roussin-Leveillee et al. 2020); however, under our growth conditions, we did not observe early flowering in the 2 knockout mutants of *ago5* examined.

The AGO5 homologs in maize (*Zea mays*), MAGO1, and MAGO2, are crucial for preventing TE mobilization during male gametogenesis upon heat stress (Lee et al. 2021). Unexpectedly, we found only *AGO5* and *AGO9* expressed in pollen, although AGO1 can silence the mir845-reporter (Borges et al. 2018) and can be precipitated from pollen (Oliver et al. 2022). Carry-over of AGO1 protein or mRNA from the microspore could explain this observation. The roles of both AGOs in female gametogenesis need to be further investigated, especially since LTR/Gypsy elements seem to also be expressed in egg cells (Sprunck et al. 2019). AGO5 was reported to be involved in megasporogenesis; however, this result was obtained with a truncated, dominant allele of AGO5 lacking the ability to selectively bind sRNAs (Tucker et al. 2012; Kawakatsu et al. 2016).

The extent to which the results from Arabidopsis reflect the situation in other plants requires further studies. However, our data from seedlings reveal remarkable similarities with the principles of TE control in germline stem cells in animals. For example, deleting PIWI-interacting RNA (piRNA) pathway components leads to strong activation of TEs in gametes and gamete-companion cells in the gonads of Drosophila, allowing different TE families to mobilize with varying strategies (Brennecke et al. 2007). While Arabidopsis, and plants in general, have diverse and partially redundant TE silencing pathways, studying gene and TE expression in single stem cells in Arabidopsis at different developmental stages, combined with information about the (sub-)cellular localization of the proteins in wild type and mutants, will also provide unprecedented insight into the complex interplay of transposon mobility and silencing along the germ track in plants with different lifetimes and propagation strategies.

## Materials and methods

### Plant material

Experiments were performed with *Arabidopsis thaliana* ecotype Col-0. The mutant and reporter lines used are listed in Supplemental Data Set 5. *AGO5* and *AGO9* reporters were cloned into *pElvis*, a derivative of *pSun* (Thomson et al. 2011) generated by inserting an additional marker conferring seed fluorescence. For this, a functional *OLE1:GFP* expression cassette (Shimada et al. 2010) was assembled from 2 PCR fragments containing promoter:CDS and GFP:terminator (fragment from pEarlyGate103 (Earley et al. 2006)), respectively, and inserted into *pSun* linearized with *EcoR*V using In-Fusion cloning (Takara Bio Cat. #121416) according to the manufacturer’s instructions.

*ProAGO5:EGFP-AGO5* was constructed by amplifying a ∼6 kb genomic fragment containing the ORF and ∼500 bp 3′ sequence and inserting it into *pElvis* using *Hind*III and *Pme*I sites. Next, a ∼2.5 kb promoter fragment was inserted via *Kpn*I and *Hind*III sites. Finally, *EGFP* was inserted using *Hind*III and In-Fusion cloning (Takara Bio Cat. #121416). For *ProAGO9:EGFP-AGO9*, a ∼5 kb fragment containing the ORF and ∼500 bp 3′ sequence was inserted into *pELVIS* using *Kpn*1 and *BamH*1. Next, the vector was cut with *Kpn*1, and a ∼3 kb promoter fragment containing the 5′UTR of *AGO9* was inserted. A *Kpn*1 site remained, and *EGFP* was inserted using in-fusion cloning (Takara Bio Cat. #121416). For *Pro35:DAAO-GFP*, a fragment containing the *CaMV35S* promoter and *DAAO-GFP* was bluntly inserted into pSUN using *Sma*I and *Hind*III filled up with Klenow fragment.

*ProAGO5:Clo-AGO5NESm* was engineered using the GreenGate system (Lampropoulos et al. 2013) by assembling the *pGGA-pAGO5*, *pGGB-Clover*, *pGGC-AGO5NESm*, *pGGD-D-dummy*, *pGGE-3UTR-AGO5*, and *pGGF-YFP*-seed-coat entry modules into *pGGSun* (*pSun* adapted for the Greengate system). For the CRISPR *ago4* lines (*ago4-CR*), sgRNAs were designed *in silico* using CHOPCHOP (Labun et al. 2019). Three sgRNAs were chosen and tested with an in vitro cleavage assay as described (Bente et al. 2020). sgRNAs that showed good cleavage efficiency on PCR products were cloned into a modified version of *pDE-Cas9* (Fauser et al. 2014) as described earlier (Bente et al. 2021) using the tRNA multiplex system (Xie et al. 2015) and 2 pre-annealed oligonucleotides for each sgRNA. The resulting sgRNA cassettes were amplified with primers containing appropriate restriction sites (*Mlu*I) and cloned into the *pDEECO* vector (Bente et al. 2020). The 2 selected sgRNAs matched against the first exon and the first intron of the *AGO4* gene. Plants were genotyped for an approximately 100 bp deletion in exon one, including the start codon.

All oligonucleotides that were used in the study are listed in Supplemental Data Set 5. Plants were transformed by the floral dip method, and transgenic seeds were selected under a fluorescence binocular microscope (Zeiss Stereomikroskop Discovery V12) based on the expression of the oleosin-GFP encoded in the plasmid backbone.

### Growth conditions

All plants were grown either in vitro on GM medium with or without selection or in soil under 16/8 h or 8/16 h light/dark cycles (for long- and short-day regimes, respectively) at 21 °C with 60% relative humidity and 150 *µ*mol m^−2^ s^−1^ light intensity. The light source consisted of white, red, and blue LED (from Photon System Instruments) with spectral peaks in blue and red. Plant material was always harvested at the same time of the light period. All plant lines and transgenic lines produced are described in Supplemental Data Set 5.

### Fixing and clearing of plant tissue

All plant tissue except mature pollen was fixed and cleared prior to microscopy using the following procedure. Samples were first fixed in a 2% FAA solution (2% formaldehyde, 5% acetic acid, 50% ethanol), as described in (Pasternak et al. 2015), for 10 min under vacuum and then placed on a thermoblock for 40 min at 37 °C. The fixative was removed, and samples were incubated in ClearSee solution (Kurihara et al. 2015) at 4 °C for 2 to 7 d. Seven-day-old seedlings were incubated in ClearSee for 4 d; for older plants, leaves were first removed, and the remaining shoots were fixed and incubated for 7 d. For inflorescence meristems, shoot tips from 35-d-old plants were placed on a Petri dish half-filled with 2% agarose, covered with distilled water, and dissected with a needle attached to a syringe to expose the SAM. Explants were fixed, cleared for 2 d, and the main stem was removed before slide preparation. Gynecia for observing egg cells and very young embryos were fixed and cleared for 7 d. Ovules with globular, heart-stage, and torpedo embryos were collected from siliques and observed after fixing and clearing for 7 d. One day before microscopy, samples were stained with 1 mg/mL DAPI in ClearSee, except for gynecia and ovules, which were stained during the whole week of clearing. Samples were washed and mounted on Superfrost microscope slides with ClearSee.

Mature pollen was released by vortexing detached flowers in a 0.3 M mannitol solution. The pollen suspension was pelleted by centrifugation for 1 min and resuspended in 20 *μ*L of the same solution. The whole suspension was loaded onto a Superfrost microscope slide for microscopy. Microscopic analysis was performed with an LSM880 Axio Observer with Airyscan detector.

### Microscopy of enlarged subepidermal cells in ovule primordia

Gynecia at different developmental stages were dissected with forceps and scalpel and fixed overnight in 4% FAA (4% formaldehyde, 5% acetic acid, 50% ethanol), then dehydrated in 70% ethanol, cleared in Herr’s solution (Herr 1971) and observed on a Zeiss Axioobserver Z1 with differential contrast optics. Materials for unfixed controls were dissected, covered with 1XPBS and directly observed.

### Counting of stem cells

Images of meristems of 7-d-old plants expressing H2B-mCherry driven by the *CLV3* promoter were acquired as 16-bit z-stacks with the same settings for all genotypes examined. Segmentation and counting of H2B-mCherry-labeled stem cell nuclei were computed with Imaris 9.5.0 software. Nuclei were identified as single spots and segmentation parameters were set to recognize spots only in the core of stem cell nuclei. The same parameters were applied for all acquisitions: Spots; Points Creation Parameters, Estimated Diameter: 3.250 3.250 3.250; Background subtraction: selected, Filter Type: quality; Lower Threshold Manual Value: 247, Upper Threshold Manual Value: 1.

### Quantification of cytoplasmic versus nuclear GFP

The cytoplasmic to nuclear GFP signal intensity ratio was quantified in meristems of 35-d-old plants after acquiring 16-bit images with the same settings in the GFP channel for each line. Cell selection for segmentation was performed based on the clarity of cell features and nonoverlap with adjacent cells. Perimeter segmentation of the cytoplasm and the nucleus was manually drawn in Fiji for each cell, and the watershed function was applied to smooth edges. The average GFP intensity signal for the cytoplasm and nucleus area was then calculated. The cytoplasmic to nuclear GFP intensity ratio for each meristem represents the average value of the selected cells. Steps were automatized using a dedicated Fiji macro.

### Fluorescence-activated nuclei sorting

The sorting of stem cells is described in detail (Gutzat and Mittelsten Scheid 2020). Pollen was harvested from flowering Arabidopsis plants as described (Johnson-Brousseau and McCormick 2004). A vacuum cleaner was equipped with 150 *µ*m and 60 *µ*m filter meshes to block unwanted plant material and debris, and pollen was collected on a final 10 *µ*m mesh. The pollen was transferred to Eppendorf tubes and stored at −80 °C in aliquots of ca. 20 *µ*L. The pollen was resuspended in 500 *µ*L Galbraith buffer (Galbraith et al. 1983) and processed as described (Borg et al. 2020) to release sperm and vegetative nuclei. The nuclei were stained by adding 0.5% v/v SYBR-Green (Life Technologies #S7563). The resulting suspension was directly subjected to fluorescence-activated nuclei sorting (FANS). Sperm and vegetative nuclei were sorted on a BD Aria III cell sorter (70 *µ*m nozzle). A 488 nm Blue Laser, Coherent Sapphire 20 mW, was used to excite SYBR-Green, and signals were detected with a FITC 530/30 nm bandpass filter. Sorting gates were adjusted according to the different emission intensities between sperm and vegetative nuclei populations. DNA and RNA isolation was performed as described (Gutzat and Mittelsten Scheid 2020).

For sorting L2 AGO9-GFP labeled nuclei, 300 apices were dissected from 3-wk-old plants grown under a short-day regime (8/16 light/dark) and plants induced to flower for 2 additional days under a long-day (16/8 light/dark) regime. The dissected apices were immersed in 3% glyoxal (Sigma #128465). The samples were vacuumed for 10 min twice and washed with Galbraith buffer. Next, the samples were ground for 1 min using a Bioruptor (Qiagen #990890), and debris was filtered (Sysmex #04-0042-2316). The mixture was centrifuged at 2000g for 3 min at 4 °C and resuspended in Galbraith buffer with 5 *µ*g/mL DAPI (Sigma #D9542-1MG). The nuclei, with different marker combinations, were sorted using a BD FACSAriaTM III Cell Sorter featuring a 70 *µ*m nozzle based on reference Col-0 nuclei. 200 nuclei were collected into each well of a 96-well-plate containing smart-seq buffer. mRNA library construction and sequencing were performed by the Next-Generation Sequencing Facility at the Vienna BioCenter (https://www.viennabiocenter.org/vbcf/next-generation-sequencing/).

### AGO5- and AGO9 immunoprecipitation and sRNA preparation

Meristems of D7 and D35 plants transgenic for GFP-tagged AGO5 and AGO9 in the background of the respective mutants were manually collected on ice. Material from 600 plants (D7) and 200 mg (D35) was frozen and ground in liquid nitrogen. The powder was suspended in IP buffer (20 mM, HEPES pH 7.5, 100 mM KCl, 0.2% NP-40, 10% glycerol, 1 mM EDTA, 1 mM PMSF, 20 *µ*M MG132, 5 mM DTT and Roche protease inhibitor #5892953001) and incubated for 1 h on a rotating wheel. This and all subsequent steps were performed at 4 °C. Cell debris was removed by centrifuging twice for 10 min at 12,000*g*. Next, the supernatants were precleared by incubation for 1 h with 200 *µ*L control beads (Chromotek #bmab-20). For the 7 d-old meristem samples, an additional step was applied to deplete AGO1 by adding 10 *µ*L anti-AGO1 (Agrisera #AS09 527) with 50 *µ*L beads (Invitrogen #10001D) and incubated for 30 min. This step was repeated once more. After bead removal, the supernatants were incubated with GFP-trap beads (Chromotek #gtma-10), 5 *µ*L for the 7 d samples, and 20 *µ*L for the 35 d samples and incubated on a rotating wheel for 1 h. The beads were washed 5 times with IP buffer. One-third of the precipitate was used for immunoblotting, and two-thirds were processed for RNA extraction in TRIzol (Invitrogen #10296010) reagent.

For immunoblotting, the precipitation was mixed with 20 *µ*L Laemmli buffer and incubated for 10 min at 95 °C. After removing the beads, the mixture was loaded on mini-PROTEAN stain-free gels (Biorad #4568083). Gel electrophoresis was performed for 90 min at 30 mA, and the gel was washed in transfer buffer (20% methanol, 0.4% SDS, 48 mM Tris, 39 mM glycine). Protein was transferred to a nitrocellulose membrane (Biorad #162-0113) by semidry electroblotting (Biorad) at 20 V for 90 min. The membrane was incubated with anti-GFP primary- (Roche #11814460001/1:2000) and antimouse (Cell Signaling Technology #7076/1:5000) secondary antibody. The membrane was washed 3 times before image acquisition using a ChemiDoc Touch Imaging System (Biorad).

sRNA libraries were constructed with QIAgen miRNA library (QIAgen #331502) and were sequenced on an Illumina HiSeqV4 SR50. All steps were performed by the Next Generation Sequencing Facility (https://www.viennabiocenter.org/vbcf/next-generation-sequencing/).

### Small RNA data analysis

Raw reads from sRNA library sequencing were trimmed using cutadapt v1.18 (Martin 2011), and 18 to 26 nt long reads were selected. The reads were aligned to the Arabidopsis genome (TAIR10 plus TAIR8 transposons, described below) using bowtie2 v2.3.5 (Langmead and Salzberg 2012), allowing 1000-fold multimapping. The 5′ nucleotides of 18 to 26 nt sRNAs were analyzed using a pipeline available on GitHub (https://github.com/AlexSaraz1/paramut_bot). Subsequent data analysis was performed with 21 to 24 nt long reads. Counting of reads was done using featureCounts from the Subread package v2.0.1 (Liao et al. 2014). Differential expression analysis was performed using DESeq2 v1.32 (Love et al. 2014) (fdr <0.05, log2-foldChange >I1I). Genomic features with <5 normalized reads were filtered out. Deeptools v.3.3.1 (Ramirez et al. 2016) was employed to generate normalized count bigwig files using bamCoverage with the “CPM” parameter. Bigwig files merged from both replicates were used to generate metaplots.

Blast+ (Camacho et al. 2009) was employed to find potential targets of AGO5-bound miRNAs. Genomic TE sequences were subjected to Blast analysis with the parameter -task blastn-short for short sequences and with miRNAs as input.

### Library preparation and sequencing

For single nuclei RNA-seq (snRNA-seq), nuclei of shoot apices of 7-d-old seedlings were prepared according to (Gutzat and Mittelsten Scheid 2020). Single nuclei of 3 different extractions were sorted into 96-well plates containing 4 *µ*l smart-seq lysis buffer (Picelli et al. 2014). Library preparation and sequencing were performed by the Next Generation Sequencing Facility (Vienna BioCenter Core Facilities). For mRNA seq of sorted stem and nonstem nuclei, bulks of 100 nuclei (each representing 1 replica) from 2 different extractions were sorted into 96-well plates and proceeded as with single nuclei. For mRNA seq of sperm and vegetative nuclei and D7 shoot apices, total RNA from 3 to 4 biological replicates (1 replicate corresponds to 80 flowering plants; each experimental series (1 replicate of each genotype) was harvested at different times) was extracted using TRIzol reagent (Invitrogen #10296010) according to the manufacturer’s description. Smart-seq2 and 3 sequencing libraries and subsequent sequencing were performed by the Next Generation Sequencing Facility (Vienna BioCenter Core Facilities). For bisulfite library preparation, libraries were prepared from 2 biological replicates with a Pico Methyl-Seq Library Prep Kit (Zymo Research #D5456) and sequenced by the Next Generation Sequencing Facility (Vienna BioCenter Core Facilities).

### Analysis of sequencing data

mRNA sequencing reads were processed with nf-core/rnaseq (Patel et al. 2021). Due to the redundancy of the TAIR annotations “transposable element” and “transposable element gene,” we used a custom annotation file containing TAIR10 features plus “transposable elements” without “transposable element genes” and added the sequences of transgenes (see below).

Differential gene expression analysis was performed with DESeq2 (Love et al. 2014). GO enrichments were calculated using the AmiGO2 tool and the PANTHER classification system (http://amigo.geneontology.org/rte) (Mi et al. 2013). Bisulfite sequencing data were processed with nf-core/methylseq (Ewels et al. 2019). Visualization of the data was achieved using R and Bioconductor (Huber et al. 2015) including the packages tidyverse, ggplot2, pheatmap, and a protocol for GO-term enrichment analysis (Bennot et al. 2019).

### RNA gel blot analysis

Twelve μg of total RNA from apices of D7 seedlings were separated on 17.5% PAGE-urea gels, blotted, and cross-linked to Hybond NX (Amersham ref. RPN203T) nylon membrane, as previously described (Incarbone et al. 2018). Probe hybridization was performed in PerfectHyb Plus buffer (Sigma ref. H7033) overnight at 42 °C, followed by 3 15-min washes in 2×SSC 2% SDS at 48 °C. miRNA160 and U6 probes were obtained by labeling DNA oligonucleotides via a PNK reaction with γ^32^ATP. To detect transposon-derived siRNA, PCR products were labeled with α^32^CTP through Klenow reaction. All primers and oligos used for the synthesis of probes are listed in Supplemental Data Set 5.

### Alignment and counting of transposable elements

With the release of the Arabidopsis genome annotation Tair8, a new transposon annotation, based on multiple homology-based predictions, has been added (Buisine et al. 2008). Existing annotations, overlapping with TE annotations, have been reclassified as locus type “transposable element gene” (https://arabidopsis.org/download_files/Genes/TAIR8_genome_release/Readme-transposons). For alignment and assigning sequencing reads to either genes, transposons (TEs), or TE genes, this creates a problem of redundant annotations. Supplemental Figure S25 shows a large proportion of TE genes overlapping with more than 1 TE and vice versa. To avoid assigning reads to both overlapping TE genes and TEs, we removed TE genes from the Tair10 annotation and added TEs as a single feature type (Tair10 + TEs).

Alignment strategies can vary in accuracy and resolution, especially for TEs (Lanciano and Cristofari 2020; O’Neill et al. 2020). To find an optimal alignment and feature counting method, we compared several alignment and quantification tools with a test data set and used DESeq2 for calculating differentially expressed features (genes and transposons). We proceeded using STAR for alignment and Salmon for read quantification, as this resulted in the smallest number of private DEGs (Supplemental Fig. S26).

### Analysis of single nuclei sequencing data

Before sorting single stem cell nuclei, sorting accuracy was confirmed by counting nuclei by microscopy and quantitative PCR for mRNA and genomic DNA. An example of the gating strategy is displayed in Supplemental Fig. S27. For sequencing, 208 single nuclei were sorted into 96-well plates. We included 2 bulk controls of 50 nuclei and 2 empty negative controls. A count matrix was generated as described above (using STAR for alignment and Salmon for read quantification). Twenty nuclei with low feature and read count were filtered out, resulting in the feature count distribution of Supplemental Fig. S15A. 217 genes with very high read counts and variance and mostly encoding genes for translational or photosynthetic processes were filtered out. We chose 4 as a cutoff based on the number of features expressed in a certain number of nuclei (Supplemental Fig. S28). Therefore, each feature (gene or transposon) was expressed in at least 4 nuclei. We performed an index sort for 1 plate and recorded every nucleus's DAPI and mCherry intensities. The sorting order did not correlate with the number of detected features, showing that mRNA leakage of nuclei during sorting is not problematic (Supplemental Fig. S29A). Surprisingly, DAPI, but not mCherry intensities, were highly correlated with the number of detected features (Supplemental Fig. S29, B to D). This shows that the cell cycle state of the nuclei contributes strongly to variation in the number of detected genes. This correlation was even slightly higher than the correlation of the number of detected genes with the number of aligned reads. We also could assign a cell cycle state to more than 90 nuclei based on the expression of HTR13 (S-G2), CDT1A (G1), and CYCB1.1 (G2-M) (Supplemental Fig. S15B).

For calculating and clustering gene-gene correlations, we first computed Spearman's correlation between all genes, and then adjusted the correlation value between every pair of genes by their sampling depth (Meir et al. 2020). In brief, this strategy subtracts the expected correlation between any pair of genes based on their expression levels only. This allows the detection of notable correlations between genes even if they were lowly sampled (which is typically the case in sparse datasets such as snRNA-seq) and vice versa, to not overestimate high correlation values between well-covered genes. For example, we show correlation and adjusted correlation values for CLV3 (Supplemental Fig. S30). For calculating TE abundance in different nucleus-types, data were processed with DESeq2.

### Accession numbers

Accession numbers plus source, NASC code, and references are listed in Supplemental Data Set 5. All materials are available from the corresponding author upon request. All sequencing data are available at the Gene Expression Omnibus under accession number GSE192611 (most data sets) and GSE239462 (data corresponding to Supplemental Figs. S17 and S18). The code used for sn-RNA-seq is available at: https://github.com/tanaylab/Meir_et_al_nat_gen_2020_clonemem/blob/master/Meir_et_al_2020_nat_gen_functions.r.

## Supplementary Material

koad295_Supplementary_Data

## Supplemental data

The following materials are available in the online version of this article.

**Supplemental Figure S1.** Localization of AGO5 and AGO9 in the SAM in different genetic lines.

**Supplemental Figure S2.** Localization of AGO5 and AGO9 in the SAM at D7.

**Supplemental Figure S3.** Localization of AGO5 and AGO9 translational reporters at different developmental stages.

**Supplemental Figure S4.** Localization of AGO5 and AGO9 translational reporters in axillary buds.

**Supplemental Figure S5.** Localization of AGO9 translational reporters.

**Supplemental Figure S6.** Localization of AGO9 translational reporters during SD to LD transition.

**Supplemental Figure S7.** Localization of AGO5 and AGO9 in samples without fixing.

**Supplemental Figure S8.** Localization of AGO5 and AGO9 translational reporters during heat stress.

**Supplemental Figure S9.** AGO5 and AGO9 have complementary functions to restrict the number of MMC precursors in ovule primordia.

**Supplemental Figure S10.** Number of *pCLV3::H2B-mCherry*-marked stem cells in D7 seedlings.

**Supplemental Figure S11.** Immuno-precipitation of GFP-AGO5 and GFP-AGO9 at D7 and D35.

**Supplemental Figure S12.** Read length distribution and 5′ bias of AGO5- and AGO9-associated sRNAs.

**Supplemental Figure S13.** AGO5 and AGO9 cargo derive from common and specific transposons throughout development.

**Supplemental Figure S14.** Annotation of AGO5- and AGO9-enriched cargo on the 5 chromosomes.

**Supplemental Figure S15.** Number of expressed features and cell cycle stages of single nuclei.

**Supplemental Figure S16.** Length of TEs expressed in single SAM stem cell nuclei.

**Supplemental Figure S17.** Sorting of SAM stem cell nuclei before and after floral induction.

**Supplemental Figure S18.** AGO expression in different SAM stem cell populations.

**Supplemental Figure S19.** Methylation levels on genes and TEs.

**Supplemental Figure S20.** Mutation of the AGO5 nuclear export signal leads to nuclear accumulation of AGO5.

**Supplemental Figure S21.** mRNA-seq analysis of sorted stem, nonstem, sperm, and vegetative (vn) nuclei.

**Supplemental Figure S22.** mRNA-seq analysis of D7 shoots apices.

**Supplemental Figure S23.** Distribution of histone variants in SAM stem cells of D7 seedlings.

**Supplemental Figure S24.** siRNAs derived from TEs targeted by AGO5 are synthesized by PolIV, RDR2, and DCL3.

**Supplemental Figure S25.** Fraction of ambiguous TE annotations.

**Supplemental Figure S26.** Comparison of different alignment strategies.

**Supplemental Figure S27.** Example of gating strategy for sorting of nuclei for snRNA-seq.

**Supplemental Figure S28.** A number of features expressed in number of nuclei.

**Supplemental Figure S29.** Comparison of FACS vs snRNA-seq data.

**Supplemental Figure S30.** Correlation and adjusted correlation to *CLV3*.

**Supplemental Data Set 1.** Enriched transposon superfamilies.

**Supplemental Data Set 2.** Cell cycle-related genes.

**Supplemental Data Set 3.** RNA-seq data of sorted nuclei.

**Supplemental Data Set 4.** Predicted miRNA targets.

**Supplemental Data Set 5.** Description of alleles and primers.

## Dive Curated Terms

The following phenotypic, genotypic, and functional terms are of significance to the work described in this paper:

CMT2 Gramene: AT4G19020
CMT2 Araport: AT4G19020

AGO6 Gramene: AT2G32940
AGO6 Araport: AT2G32940

RDR2 Gramene: AT4G11130
RDR2 Araport: AT4G11130

AGO5 Gramene: AT2G27880
AGO5 Araport: AT2G27880

AGO4 Gramene: AT2G27040
AGO4 Araport: AT2G27040

CDT1A Gramene: AT2G31270
CDT1A Araport: AT2G31270

MCT2 Gramene: AT5G07930
MCT2 Araport: AT5G07930

MGH3 Gramene: AT1G19890
MGH3 Araport: AT1G19890

AGO1 Gramene: AT1G48410
AGO1 Araport: AT1G48410

RDR6 Gramene: AT3G49500
RDR6 Araport: AT3G49500

DUO1 Gramene: AT3G60460
DUO1 Araport: AT3G60460

DDM1 Gramene: AT5G66750
DDM1 Araport: AT5G66750

pollen AmiGo: PO:0025281

AGO10 Gramene: AT5G43810
AGO10 Araport: AT5G43810

AGO8 Gramene: AT5G21030
AGO8 Araport: AT5G21030

AGO9 Gramene: AT5G21150
AGO9 Araport: AT5G21150
