# Supplementary material for: Two ARGONAUTE proteins loaded with transposon-derived small RNAs are associated with the reproductive cell lineage in Arabidopsis
Source: Plant Cell. 2023 Dec 7;36(4):863–80. doi: 10.1093/plcell/koad295 (PMC10980394; doi:10.1093/plcell/koad295)
Supplement: koad295_Supplementary_Data [file koad295_supplementary_data.zip › tpc.23.00052Supplemental Figures.pdf]

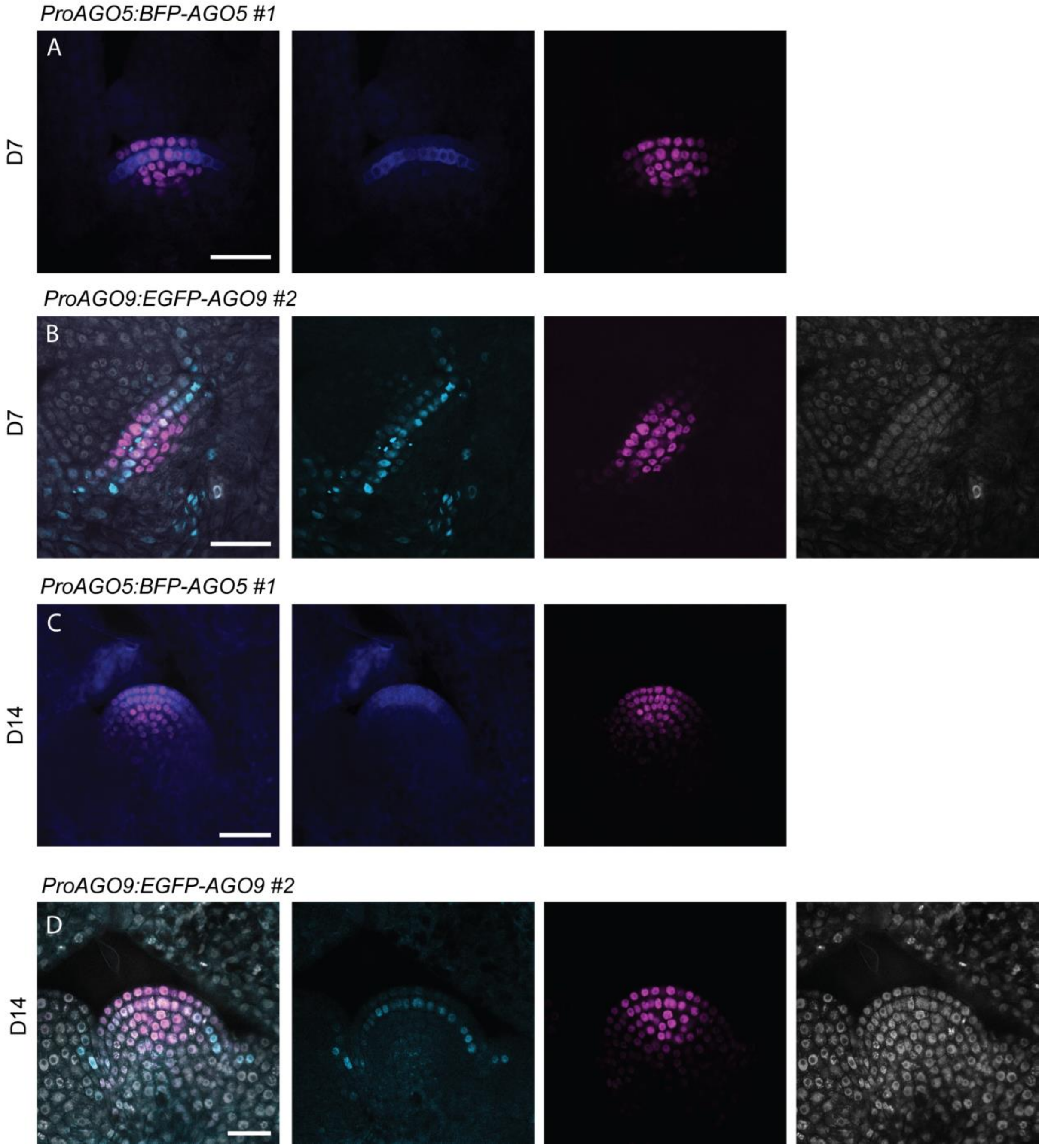

Supplemental Figure S1 | Localization of AGO5 and AGO9 in the SAM in different genetic lines.

a, **b**, localization of BFP-AGO5 and GFP-AGO9 in the SAM at D7. **c**, **d**, localization of BFP-AGO5 and GFP-AGO9 in the SAM at D14. These lines were constructed as described in the main text but represent independent transformants. Scale bar = 20 µm Supports Figure 1.

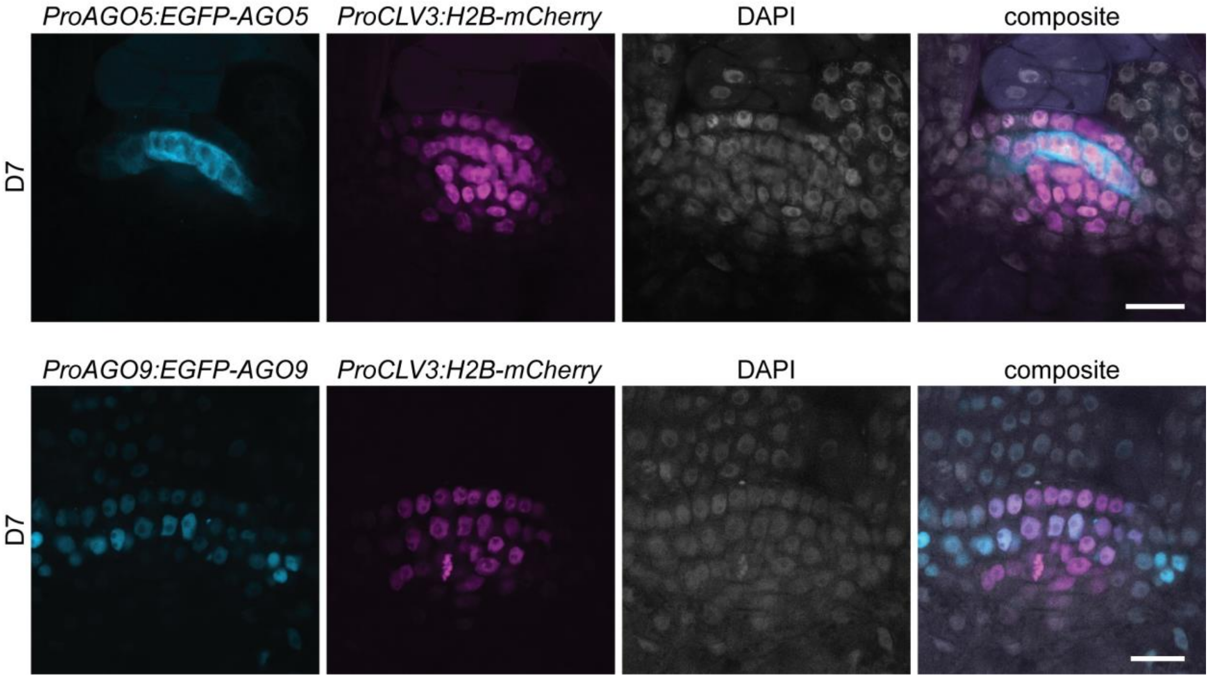

Supplemental Figure 2 | Localization of AGO5 and AGO9 in the SAM at D7.

a-**d**, localization of AGO5 in the SAM at D7, single channels (**a** = GFP, **b** = RFP, **c** = DAPI), and composite image (**d**). **e**-**h**, localization of AGO9 in the SAM at D7, single channels (**e** = GFP, **f** = RFP, **g** = DAPI), and composite image (**h**). Scale bar **d, h** = 20 µm. Images **d** and **h** are also used in Fig. 1a, b. Supports Figure 1.

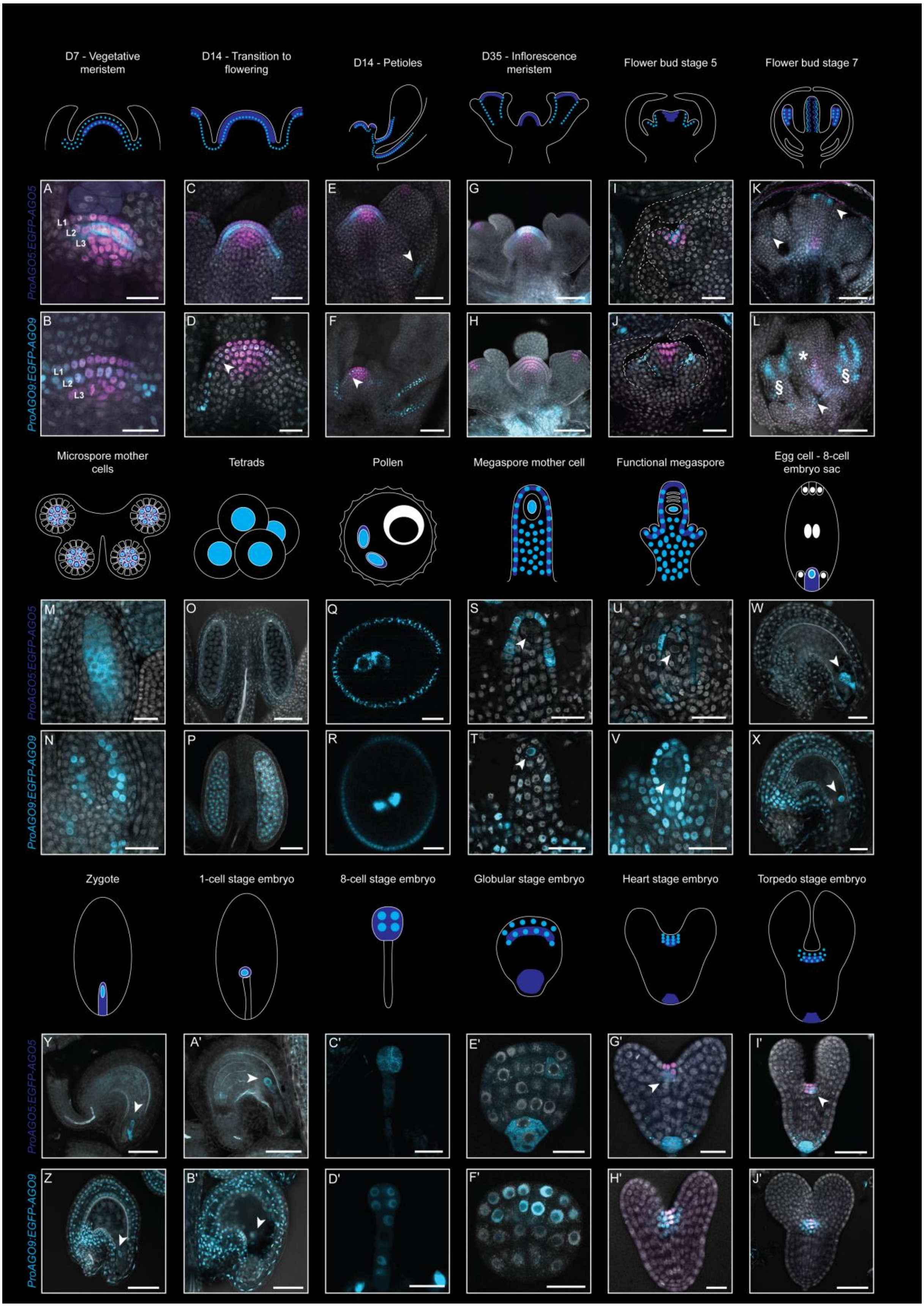

Supplemental Figure S3 | Localization of AGO5 and AGO9 translational reporters at different developmental stages.

a, as Fig. 1a. **b,** as Fig. 1b. **c,** as Fig. 1c. **d,** as Fig. 1d. **e,** AGO5 is localized in the

L1, L2, and on the adaxial side at the base of leaf petioles (arrowhead), where axillary meristems emerge. **f,** AGO9 is localized explicitly in the L2, and the signal is found, as a continuum, along the adaxial side of leaf petioles. **g,** as Fig. 1e. h, as Fig. 1f. **i,** At Stage 5, AGO5 localization in the L1 follows the morphological changes that start shaping the two carpels, and, at this point, it overlaps with stem cell nuclei. **j,** At stage 5 of flower development, AGO9 is exclusively localized between the whorl of carpels and stamens. **k,** as Fig. 1g. **l,** as Fig. 1h. **m,** as Fig. 1i. **n,** as Fig. 1j. **o,** AGO5 is absent from microspore tetrads in contrast to **p,** AGO9. **q,** as Fig. 1k. **r,** as Fig. 1l. **s,** as Fig. 1m. **t,** as Fig. 1n. **u,** At Stage 4 of ovule development, AGO5 is restricted to the L1 and inner integuments and absent from the functional megaspore (FM) (arrowhead). **v,** AGO9 is localized to the FM (arrowhead) in the L1 and the surrounding integuments of Stage 4 ovules. **w,** as Fig. 1o. x, as Fig. 1p. **y,** as Fig. 1q. **z,** as Fig. 1r. **a',** AGO5, and (**b'**) AGO9 are localized in the 1-cell embryo and the suspensor (arrowheads). **c',** AGO5, and (**d'**) AGO9 are present in the body and suspensor of the octant stage embryo. **e',** as Fig. 1s. **f',** as Fig. 1t. **g',** as Fig. 1u. **h',** as Fig. 1v. **i',** Localization in the torpedo stage reveals the same pattern for AGO5 as observed in the heart stage (Fig. 1u) (arrowhead). **j',** In the torpedo stage, the AGO9 domain is broader than the CLV3 domain, and it reaches, to some extent, the basal regions of the cotyledons. Scale bar I, j, u, v, c', d'= 20 µm. Scale bar e, f, o, p, a', b', i', j' = 50 µm. Supports Figure 1.

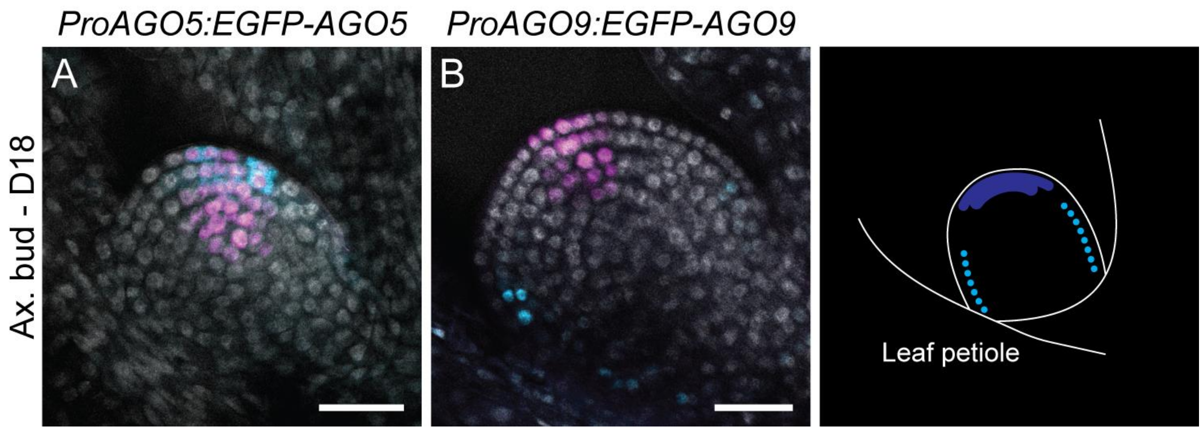

Supplemental Figure S4 | Localization of AGO5 and AGO9 translational reporters in axillary buds. a, AGO5 is localized in the L1 and L2 of axillary buds at D18. **b,** AGO9 is localized at the periphery of axillary buds at D18. Scale bar = 20 µm. Supports Figure 1.

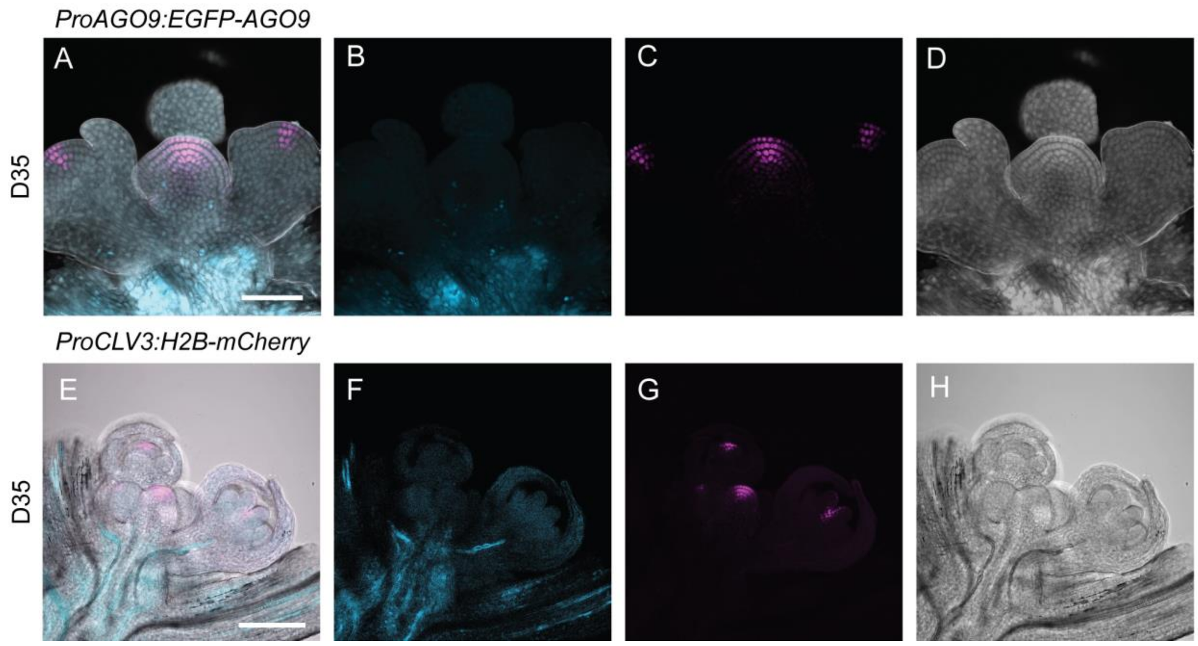

Supplemental Figure S5 | Localization of AGO9 translational reporters. Upper panel (**a-d**): localization of AGO9 in the SAM at D35, single channels (**b-d**), and composite image (**a**). Lower panel (**e-h**): Control images of the pCLV3:H2B-mCherry reporter, showing high autofluorescence below the meristem. Scale bar a-d = 50 µm, e-h = 100 µm. Supports Figure 1.

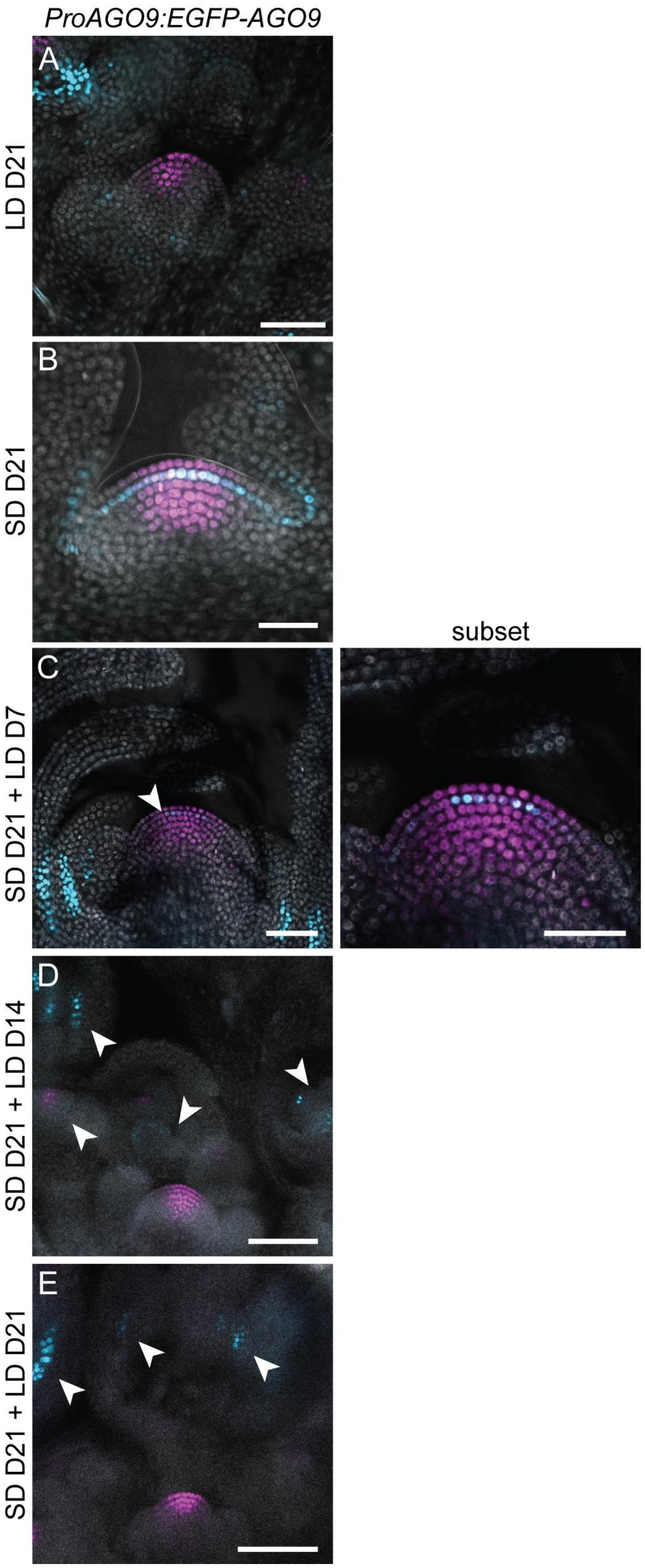

ProAGO9:EGFP-AGO9
A
LD D21
B
SD D21
C
SD D21 + LD D7
subset
D
SD D21 + LD D14
E
SD D21 + LD D21

Supplemental Figure S6 | Localization of AGO9 translational reporters during SD to LD transition. **a,** At D21 in long days, AGO9 is not present in the SAM, but can be detected in developing flowers. **b,** At D21 in short days (SD), AGO9 is specifically localized in the L2 of the SAM and leaf primordia. **c,** three weeks in SD followed by a week of long days (LD D7) results in minor localization changes with AGO9 signal intensity only decreasing in intensity in the L2 (arrowhead + subset) compared to (**b**). **d,** After three weeks of SD and two (D14), or three (D21) (**e**) weeks of LD, AGO9 has disappeared from the SAM and can be detected in flowers instead (arrowheads). Scale bar **a, b** = 50 µm. Scale bar **c, d** = 100 µm. Supports Figure 1.

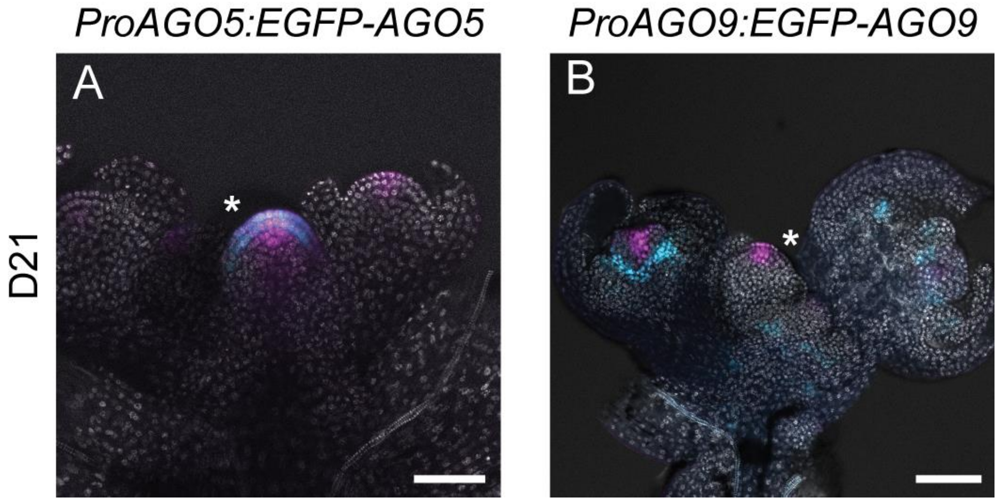

Supplemental Figure S7 | Localization of AGO5 and AGO9 in samples without fixing. Samples were not fixed and life tissue directly observed. * Indicates the SAM. Scale bar = 50 µm. Supports Figure 1.

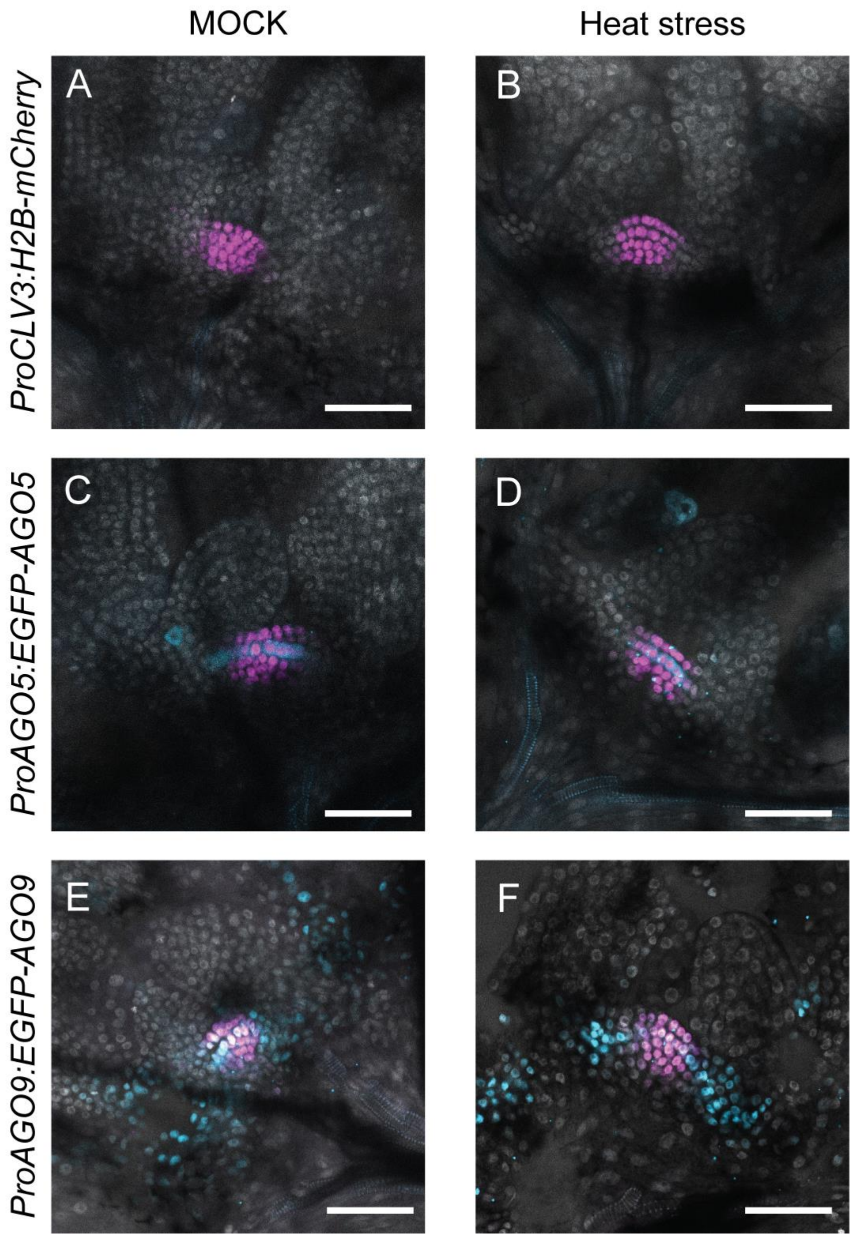

Supplemental Figure S8 | Localization of AGO5 and AGO9 translational reporters during heat stress. D7 Seedlings were either mock (**a, c, e**) or heat treated (**b, d, f**, 24h at 37°C). Scale bar = 50 µm. Supports Figure 1.

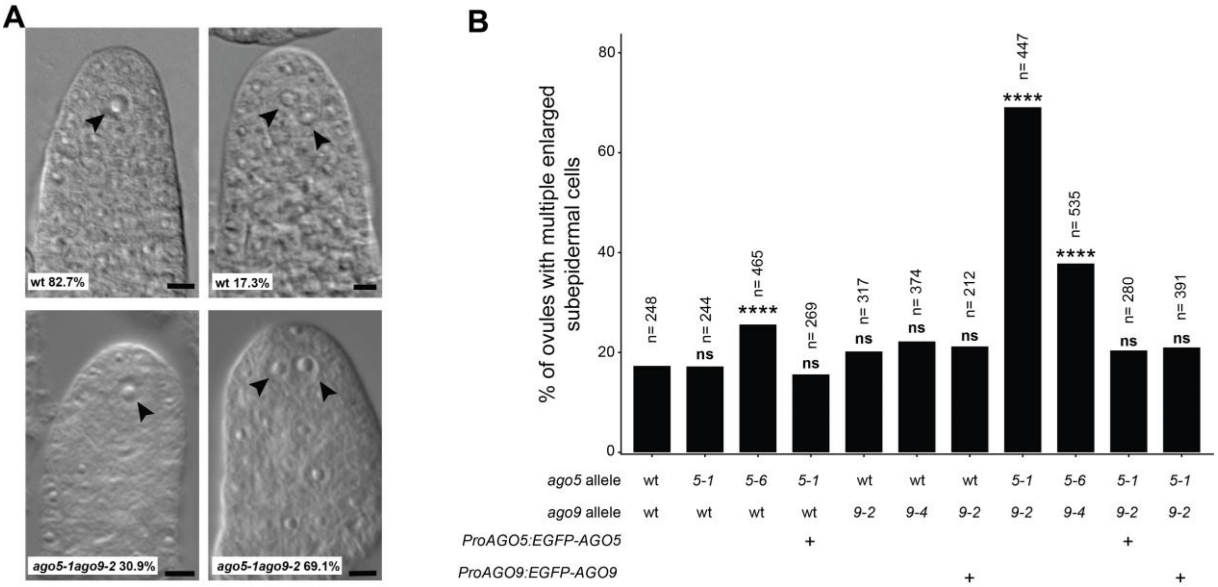

Supplemental Fig. S9 | AGO5 and AGO9 have complementary functions to restrict the number of MMC precursors in ovule primordia. a, Gynoecia were analyzed by Nomarski interference contrast microscopy to determine the percentage of ovules with more than one enlarged subepidermal cell. Top left: a wt ovule showing one subepidermal cell. Top right: a wt ovule with two subepidermal cells. Bottom left: an *ago5-1 ago9-2* ovule with a single subepidermal cell. Bottom right: an *ago5-1 ago9-1* showing two subepidermal cells. Black arrows indicate enlarged subepidermal cells. Scale bar = 5µm. **b**, Statistical analyses of numbers of pre-meiotic ovules of indicated genotypes with multiple enlarged subepidermal cells. Statistical significance using the binominal test was performed against the wt. ****p-value < 0.0001, ns = not significant. + indicates the presence of the reporter line. N indicates the total number of observed ovules. Ovules were prepared from ca. 20 plants.

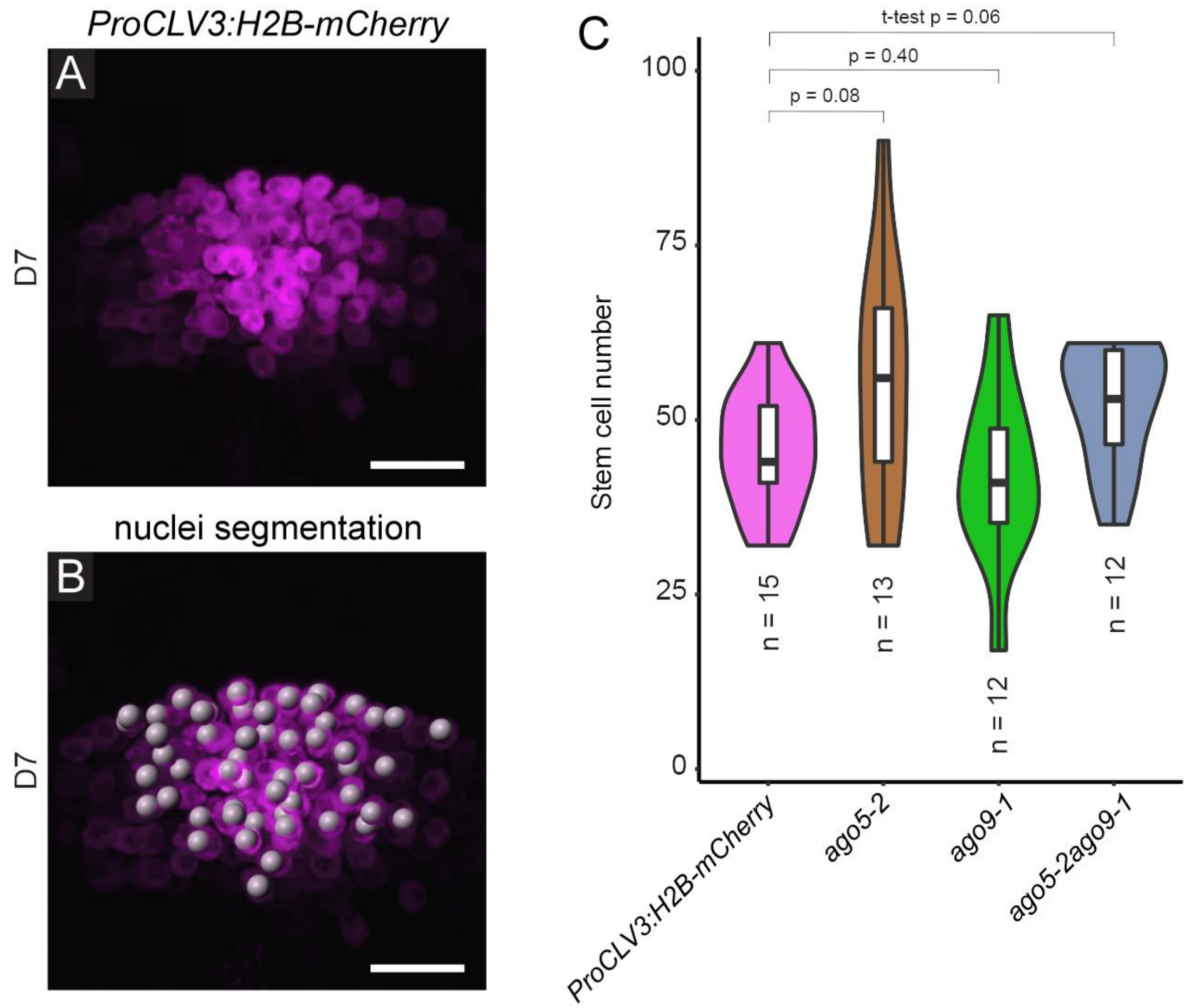

Supplemental Fig. S10 | Number of *pCLV3::H2B-mCherry*-marked stem cells in D7 seedlings. **a**, Representative image of *pCLV3:H2B-mCherry*-marked stem cells in a D7 seedling. **b**, Nuclei segmentation performed by Imaris software and overlaid on the image in a. **c**, The number of stem cells in different lines. Box plots represent the median, upper and lower quartiles, and 1.5x interquartile range. All mutant lines are in the *pCLV3::H2B-mCherry* background. n indicates the number of analyzed seedlings. Statistics were performed using a two-tailed t-test. Scale bar **a, b** = 10 µm.

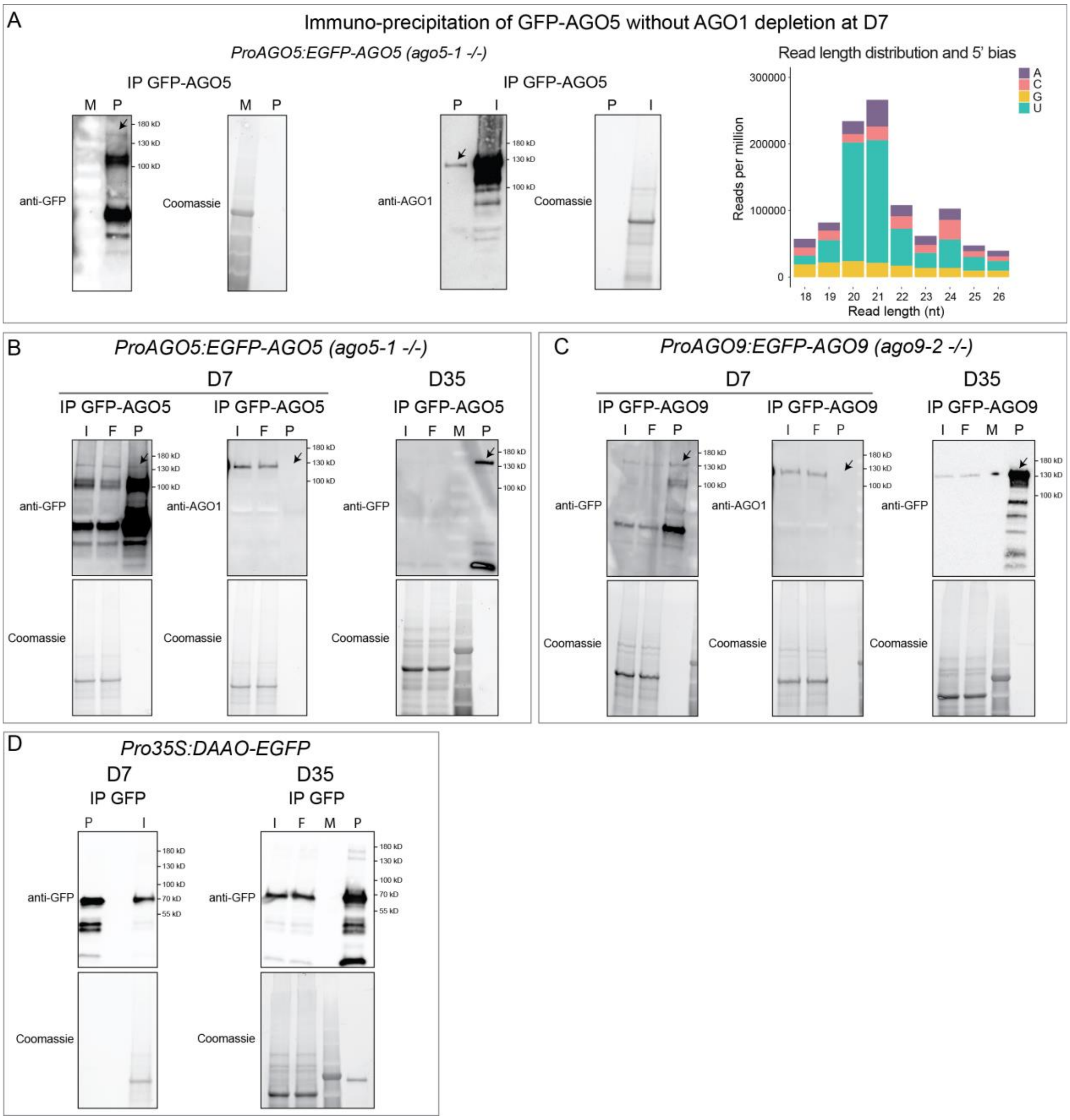

Supplemental Fig. S11 | Immunoprecipitation of GFP-AGO5 and GFP-AGO9 at D7 and D35. a, Initial analysis of AGO5-bound sRNAs at D7 revealed high content of 21 nt sRNAs with 5' U similar to sRNAs described associated with AGO1 (shown on the right side). Therefore, we tested the AGO5 precipitate for AGO1 contamination. Indeed, AGO1 was detectable in the AGO5 precipitate (arrow, immunoblot in the middle). **b**,**c**,**d**, Prior to AGO5 or AGO9 precipitation, we included an AGO1 depletion step and controlled for the absence of AGO1 in the precipitate. Immunoprecipitation of GFP-AGO5 (b), GFP-AGO9 (c), and GFP control (d) at D7 and D35. For the GFP-control (lower panel) we used a line expressing a constitutive p35S::DAAO-GFP. Arrows indicate corresponding signals. Nomenclature: IP (immunoprecipitation), P (precipitate), I (Input), F (Flow), M (protein ladder). Supports Figure 2.

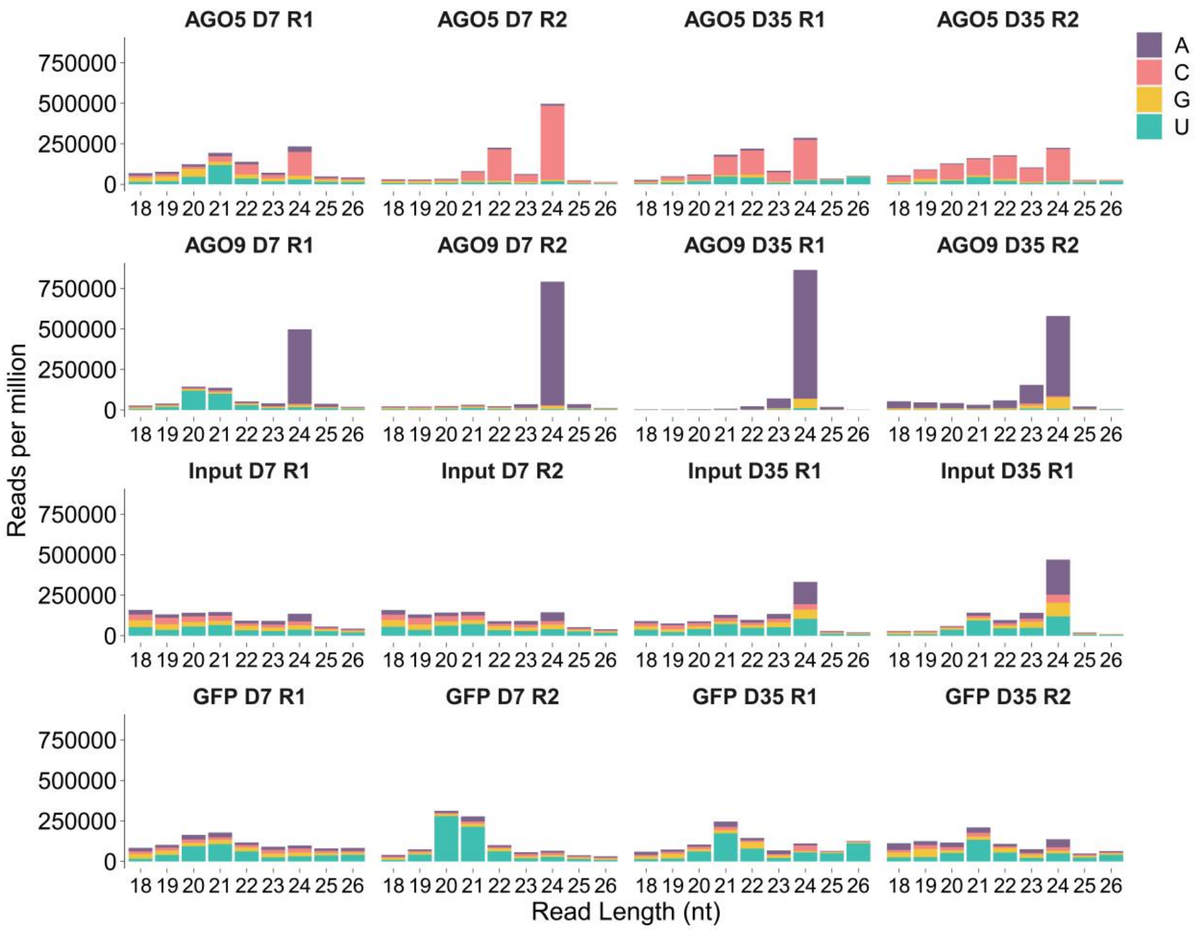

Supplemental Fig. S12 | Read length distribution and 5' bias of AGO5- and AGO9-associated sRNAs. Results are from two independent replicas (R1 and R2). For the GFP-control (lower panel), we used a line expressing a constitutive p35S:DAAO-GFP. Supports Figure 2.

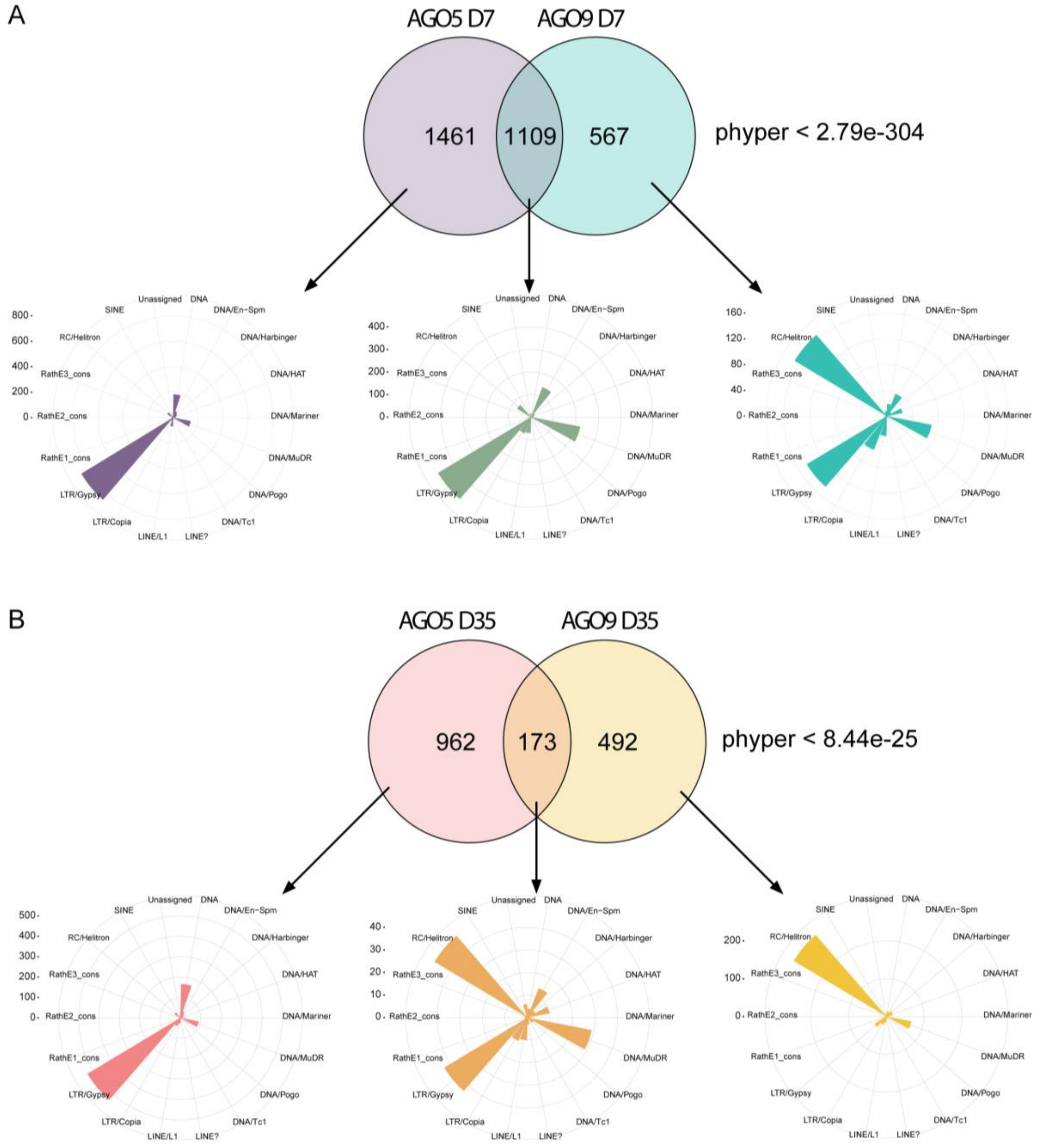

Supplemental Fig. S13 | AGO5 and AGO9 cargo derives from common and specific transposons throughout development. Venn diagrams show the number of overlapping targets and p-values at D7 (a) and D35 (b). The polar charts indicate the composition of corresponding superfamilies. Supports Figure 2.

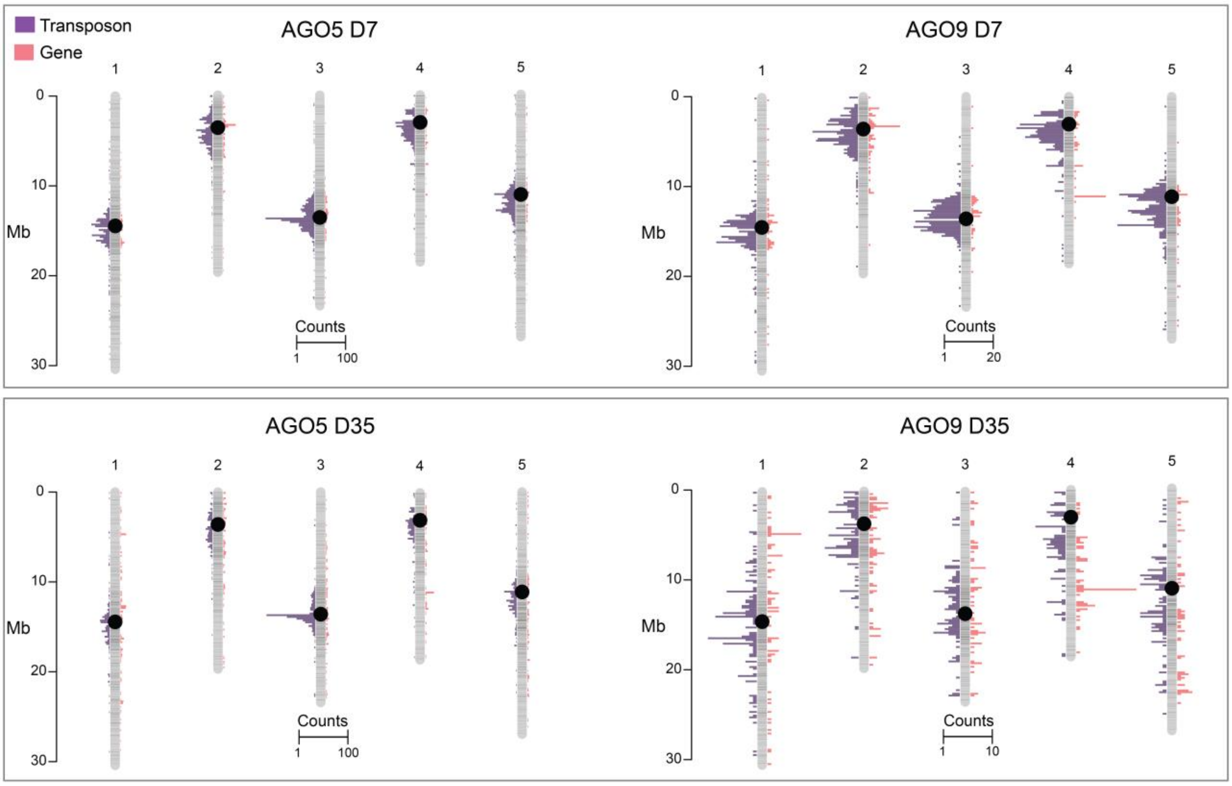

Supplemental Fig. S14 | Annotation of AGO5- and AGO9-enriched cargo on the five chromosomes. AGO5-bound sRNAs at D7 (a) and D35 (c) and AGO9-bound sRNAs at D7 (b) and at D35 (d). Supports Figure 2.

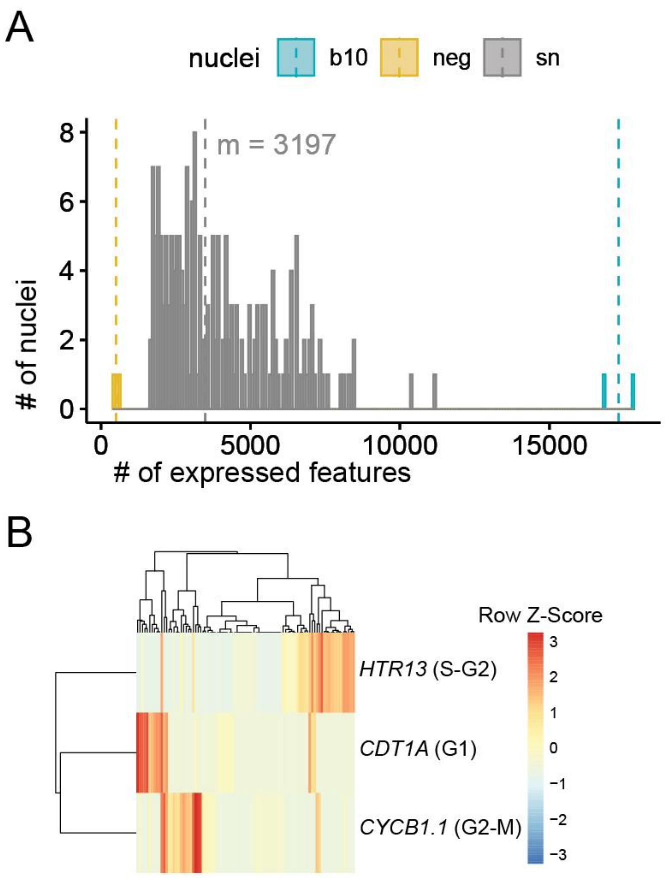

Supplemental Fig. S15 | Number of expressed features and cell cycle stages of single nuclei. a, Number of detected features (genes and TEs) in single nuclei (sn), negative controls (neg), and bulk controls of 50 nuclei (b10). **b,** Cell cycle state of individual SAM nuclei. Shown are expression values. Three cell cycle reporter genes separate individual nuclei into three major clusters.

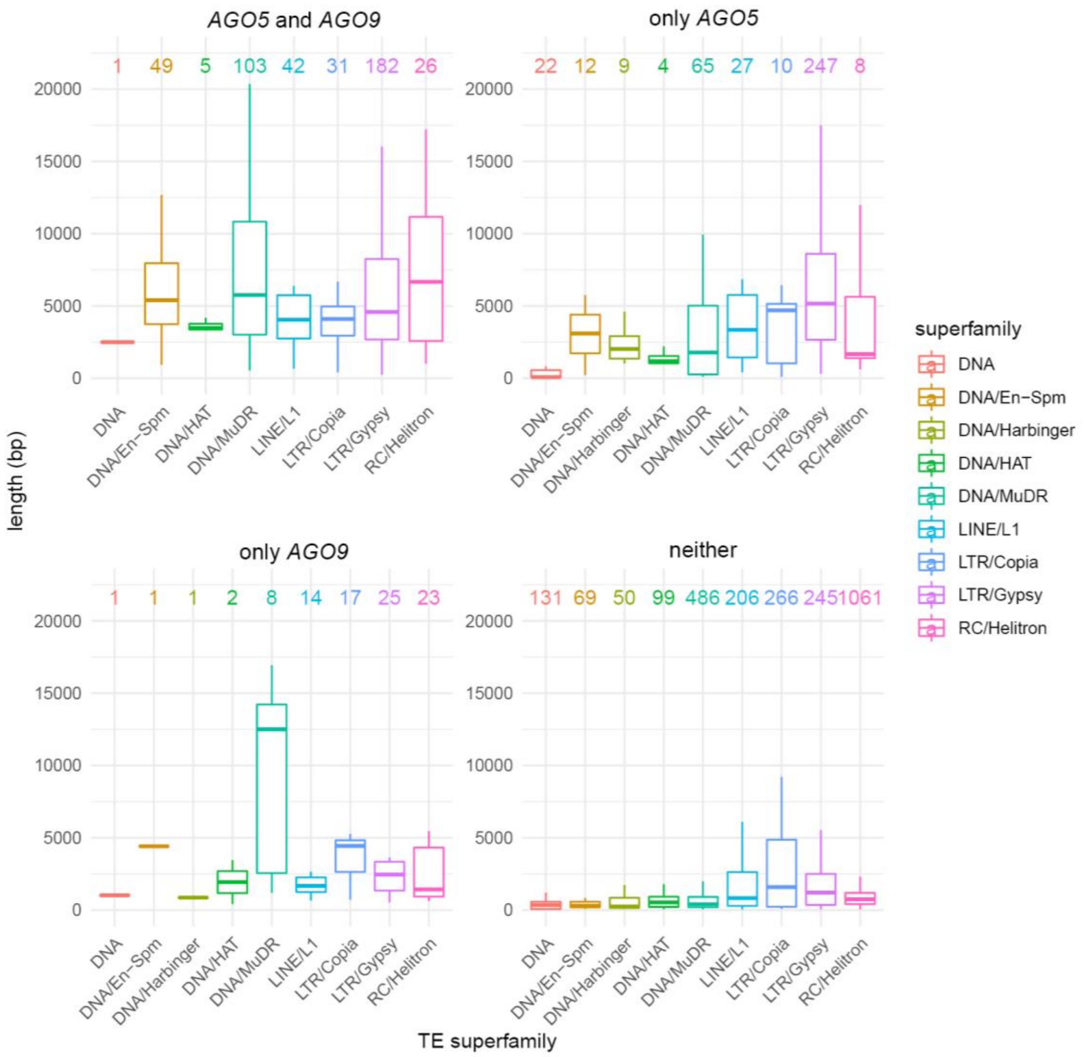

Supplemental Fig. S16 | Length of TEs expressed in single SAM stem cell nuclei. Groups indicate whether TEs are processed into sRNAs associated with AGO5 and AGO9, only AGO5, only AGO9, or neither. Colored numbers show the number of TEs in the respective superfamily. Box plots represent the median, upper and lower quartiles, and 1.5x interquartile range.

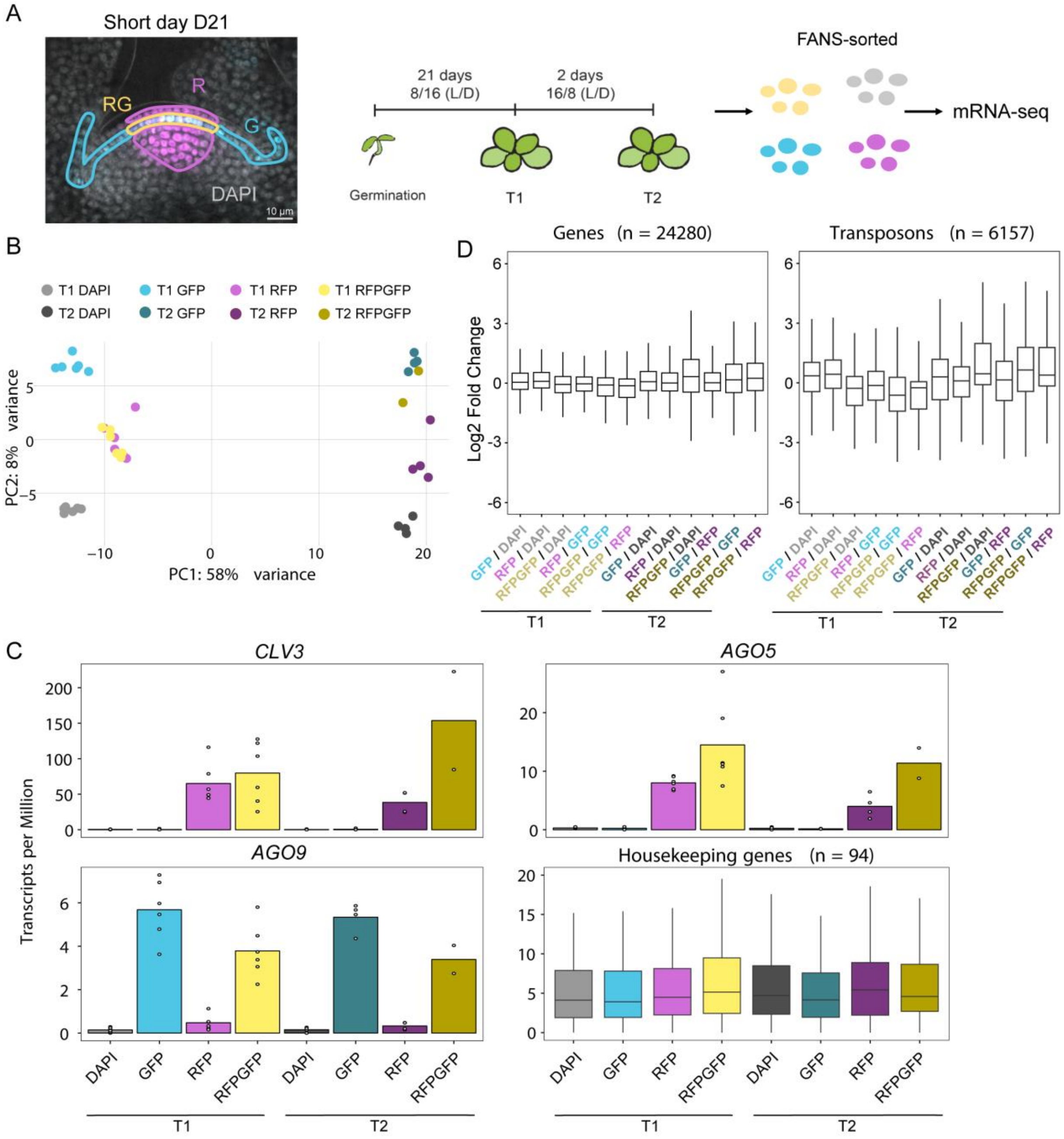

Supplemental Fig. S17 | Sorting of SAM stem cell nuclei before and after floral induction.

a, Experimental procedure: cross section of a meristem of a 21 day-old seedling containing the pAGO9:GFP-AGO9 and pCLV3:H2B-mCherry reporters. The image presented here corresponds to the one shown in Supplemental Fig. 6b, overlaid with outlines to highlight the different cell populations: stem cells (marked with RFP in red), L2 cells (marked with GFP in green), L2 stem cells (marked by the co-localization of RFP and GFP in yellow (RFPGFP). To perform the analysis, we sorted four populations of cell nuclei (including DAPI-stained nuclei), in bulks consisting of 200 nuclei at two specific timepoints: from the apices of 21 day-old plants that were grown under short-day conditions (referred to as T1), and from the apices of 23-day-old plants that were transferred to long-day conditions for two days to induce flowering (referred to as T2) (You et al., 2017). These sorted nuclei were then subjected to sequencing using smart-seq3. b, PCA analysis of the sequencing data showing the different populations of nuclei. c, Expression of the reporter gene for stem cells (CLV3 and AGO5), the L2 (AGO9) and a set of housekeeping genes (Czechowski et al. 2005). d, Expression differences of the indicated pair-wise comparisons at both time points for genes and transposons with non-zero Log_2-fold change. Genes and transposons with less than 10 reads in the data set were filtered out.

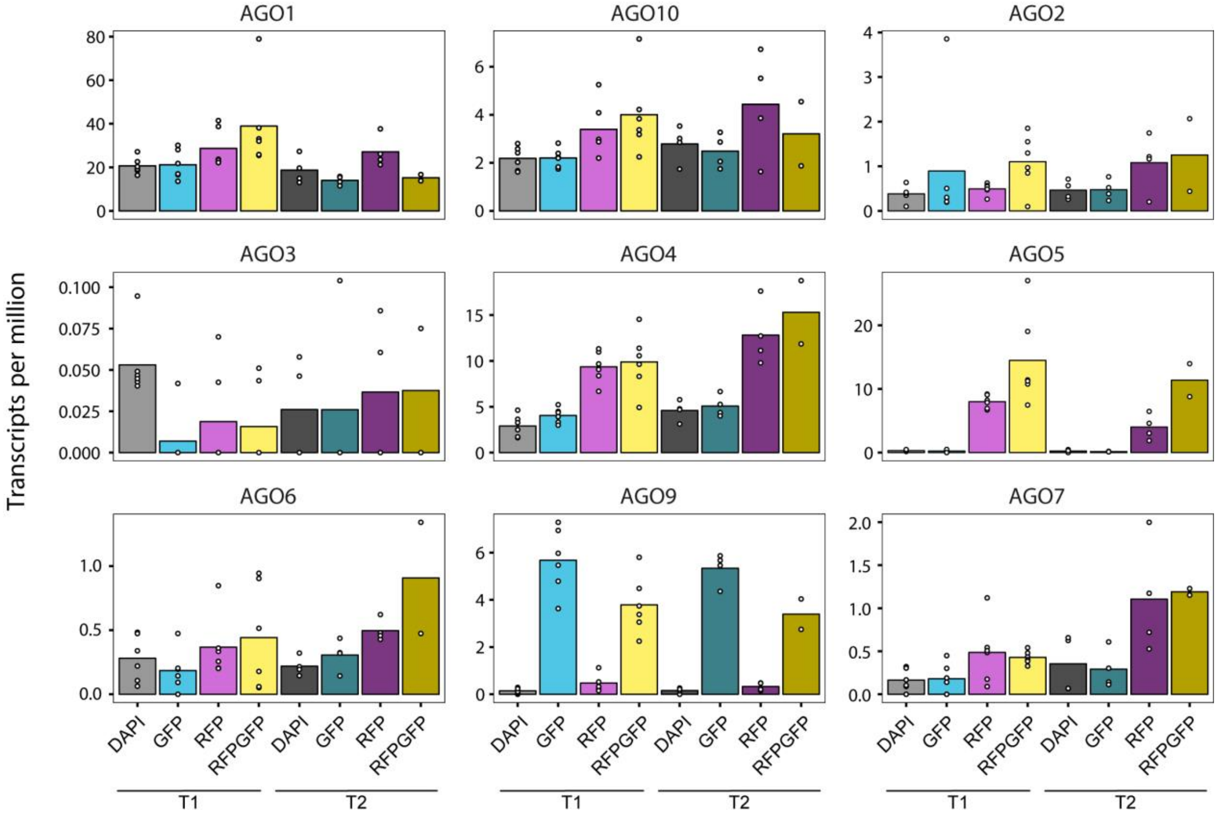

Supplemental Fig. S18 | *AGO* expression in different SAM stem cell populations.

For experimental details, see Supplemental Fig. 17. Expression of *AGO* genes in different sub-populations of stem cells. DAPI represents meristematic cells, GFP represents L2 cells, RFP represents stem cells, RFPGFP represents L2 stem cells. T1 and T2 represent two time points before and after floral induction.

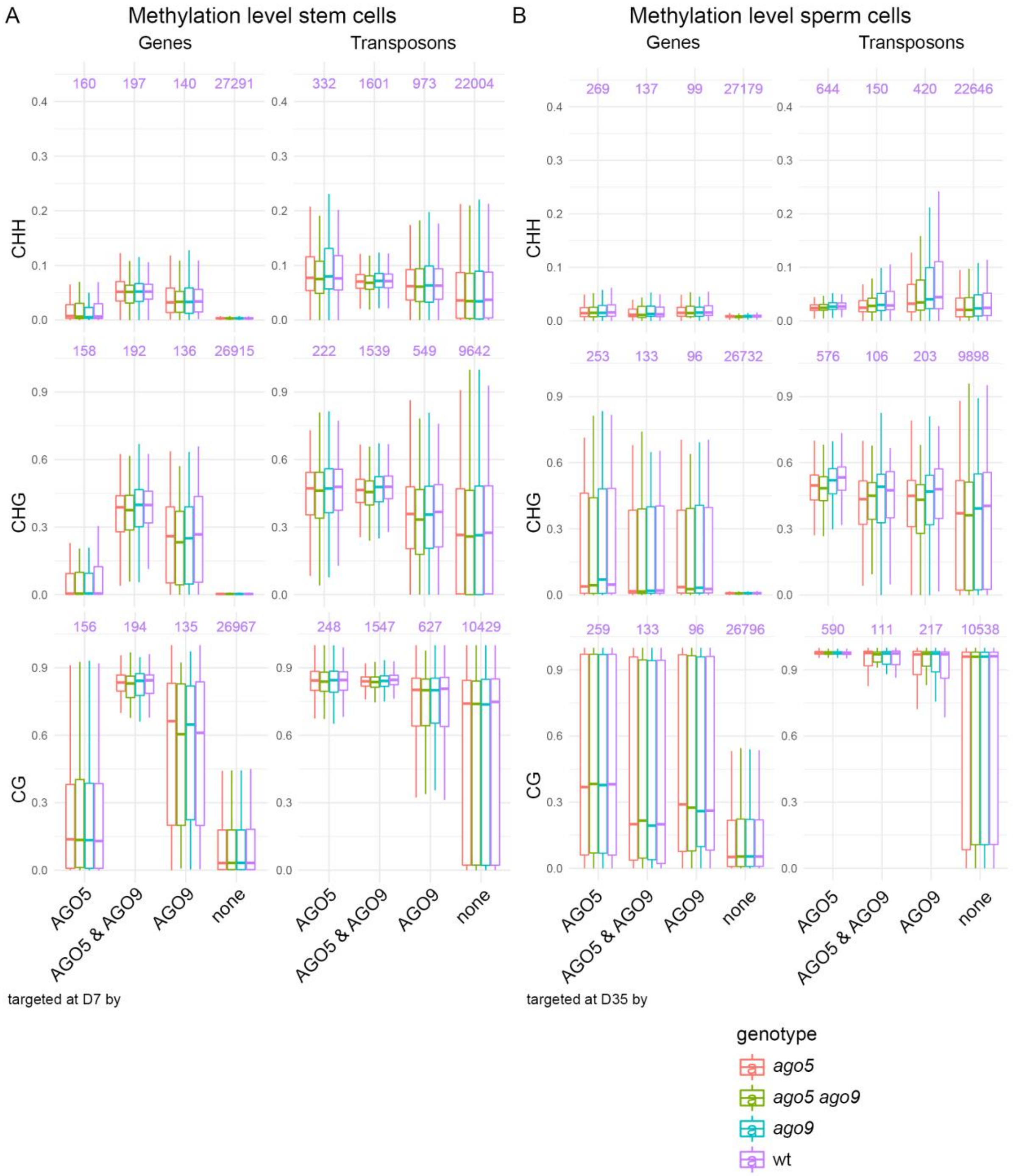

Supplemental Fig. S19 | Methylation levels on genes and TEs. a, Methylation levels of stem cells of *ago5*, *ago9*, *ago5 ago9*, and wt on genes and TEs targeted either by AGO5, AGO9, both, or none, at D7. Purple numbers indicate the number of targets. **b**, Methylation levels of sperm cells of *ago5*, *ago9*, *ago5 ago9*, and wt, on genes and TEs targeted either by AGO5, AGO9, both, or none, at D35. Purple numbers indicate the number of targets. Box plots represent the median, upper and lower quartiles, and 1.5x interquartile range.

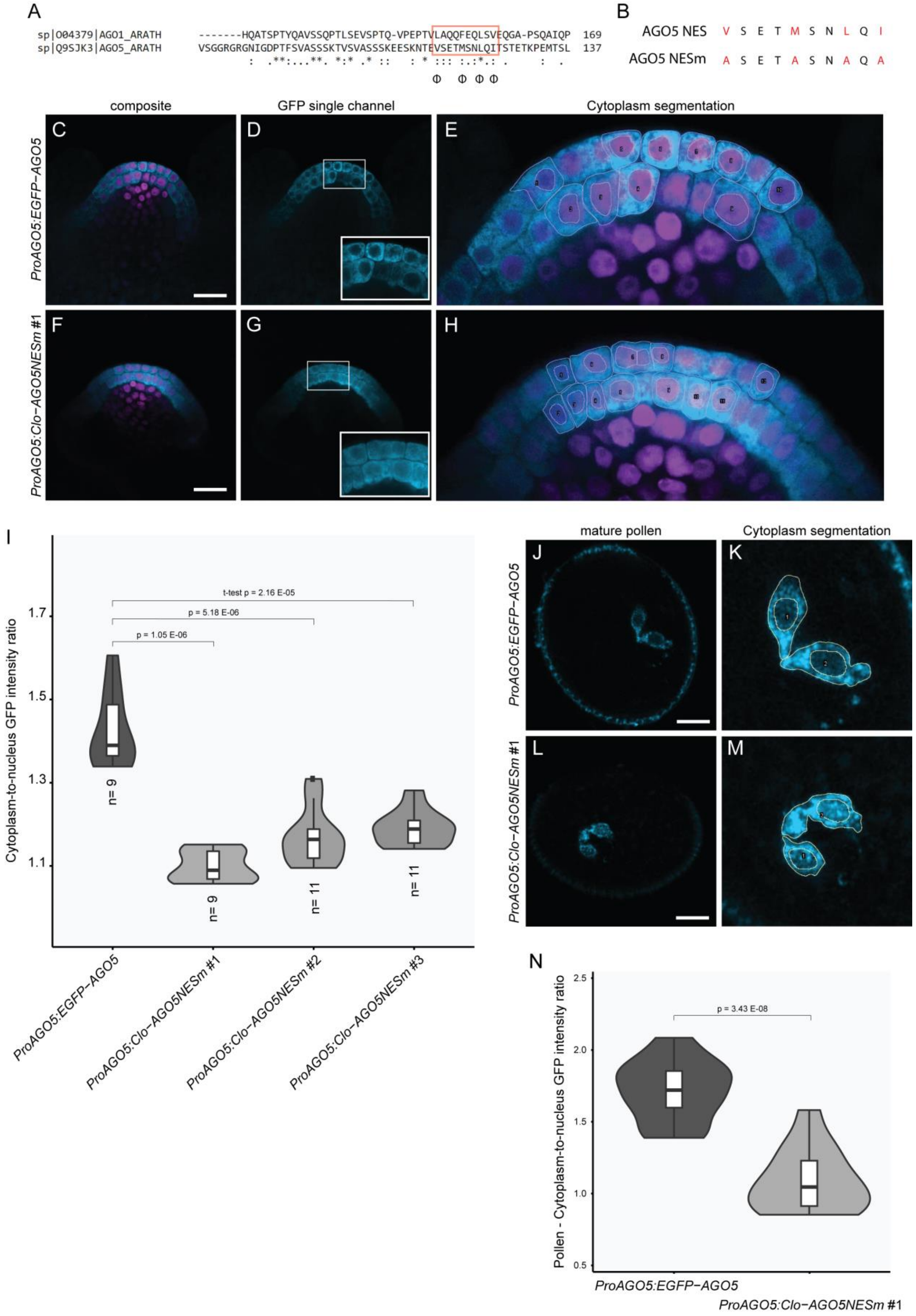

Supplemental Figure S20 | Mutation of the AGO5 nuclear export signal leads to nuclear accumulation of AGO5. a, Clustal O (1.2.4) amino-acid sequence alignment between AGO1 and AGO5. The red box

identifies the presumptive NES, and Φ represents either L, I, V, M, or F, as described for AGO1 (Bologna et al. 2018). **b**, Alanine substitutions are indicated for AGO5 NESm. **c**, Representative SAM image of *pAGO5::EGFP-AGO5* #1 at D35. **d**, GFP single channel of image c; inset: apical GFP labelled cells. **e**, Subset of output image derived from automated cytoplasm segmentation of image c. **f**, Representative SAM image of *pAGO5:Clo-AGO5NESm* #1 at D35. **g**, GFP single channel of image f; inset: apical GFP labeled cells. **h**, Subset of output image derived from automated cytoplasm segmentation of image f. Yellow lines outline the nucleus and cytoplasm perimeter of cells. **i**, Cytoplasm-to-nucleus GFP intensity ratio of L1 and L2 apical cells in D35 plants. Box plots represent the median, upper and lower quartiles, and 1.5x interquartile range. **j**, Representative pollen image of *pAGO5:EGFP-AGO5* #1. **k**, Subset of output image derived from automated cytoplasm segmentation of image j. **l**, Representative pollen image of *pAGO5::Clo-AGO5NESm* #1. **m**, Subset of output image derived from automated cytoplasm segmentation of image l. Yellow lines outline the nucleus and cytoplasm perimeter of cells. **n**, Cytoplasm-to-nucleus GFP intensity ratio of sperm cells. Box plots represent the median, upper and lower quartiles, and 1.5x interquartile range. All lines are in the *pCLV3::H2B-mCherry* and *ago5-1* mutant background. n indicates the number of apices analyzed. Between 9 to 21 cells per apex were used for the calculations. 14 pollen grains per line were used for the calculations. Statistics were performed employing a two-tailed t-test. Scale bar **c** and **f** = 20 µm; **j** and **l** = 5 µm.

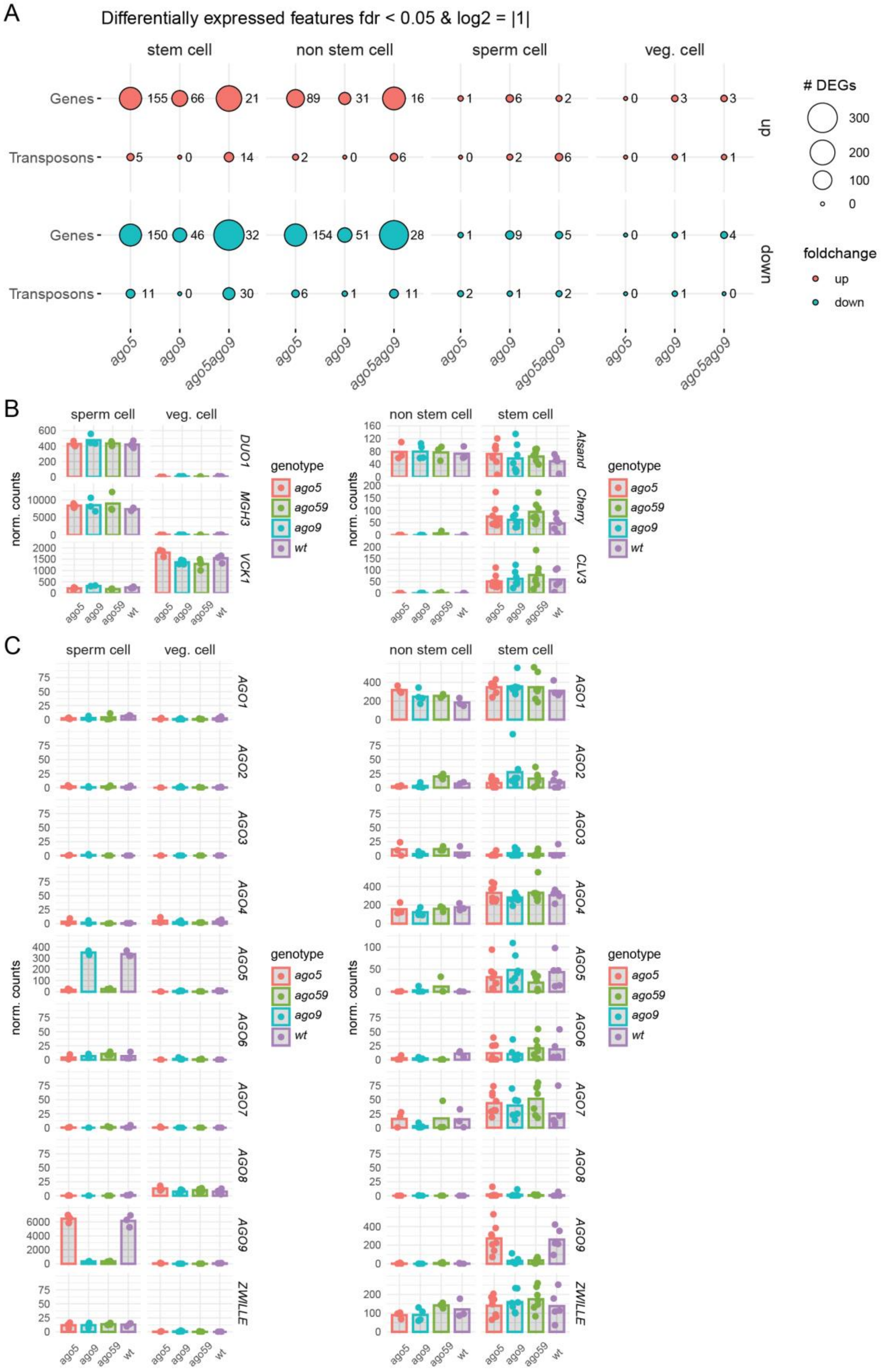

Supplemental Fig. 21 | mRNAseq analysis of sorted stem, non-stem, sperm, and vegetative (vn) nuclei. a, Balloon plot representing the number of DEGs for comparison with the wt (Wald test).

b, Expression of *DUO1*, *MGH3* (reporter for sperm cells), *VCK1* (reporter for vn cells), *CLV3*, mCherry

(reporter for stem cells), and *AtSand* (ubiquitously expressed). **c**, Expression of all 10 *AGO* genes. Reads aligning to *AGO5* in stem cells of *ago5* and *ago5 ago9* (ago59) map to the region before the T-DNA insertion in the mutant. N = 3 (wt, *ago5*, *ago5ago9* non stem cell; wt, *ago5*, *ago9*, *ago5ago9* sperm cell; *ago5* vn cell), N = 4 (*ago9* non stem cell; wt, *ago9*, *ago5ago9* vn cell), N = 5 (wt stem cell), N = 7 (*ago9*, *ago5ago9* stem cell), N = 8 (*ago5* stem cell).

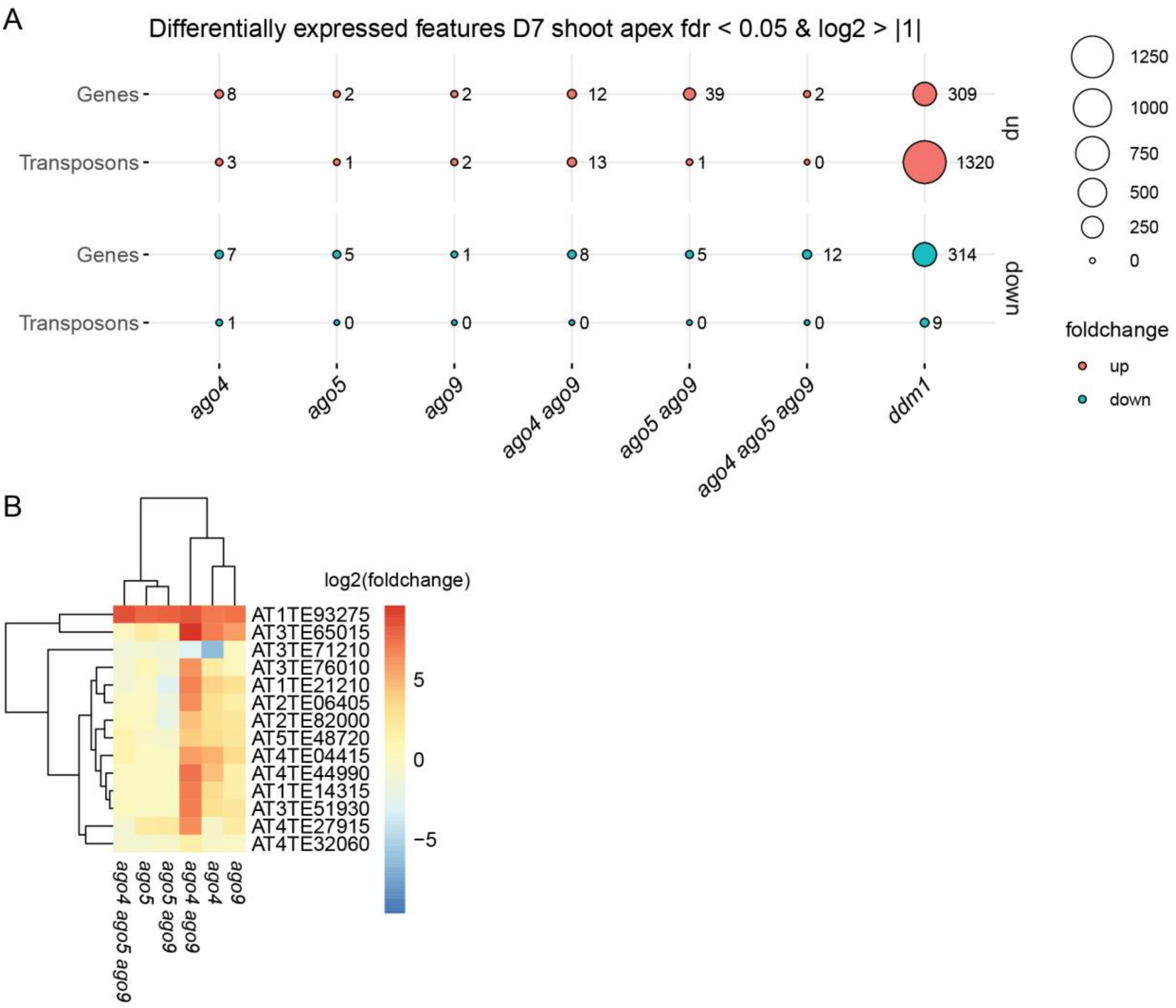

Supplemental Fig. S22 | mRNAseq analysis of D7 shoot apices. a, Number of differentially expressed genes and TEs (DEGs) in D7 shoot apices of indicated mutants compared to wild type (Wald test). **b**, Clustering of log2-fold changes of TEs with increased expression in *ago* mutants showing synergistic effects of AGO4 and AGO9 on the expression of transposons. Some differences between *ago4 ago9* and *ago4 ago5 ago9* might originate from different mutant alleles for *ago4*, as we had to generate the allele in the triple mutant with CRISPR due to the genetic linkage of *AGO4* and *AGO5* on chromosome two. N = 4 (all genotypes except wt), N = 8 (wt).

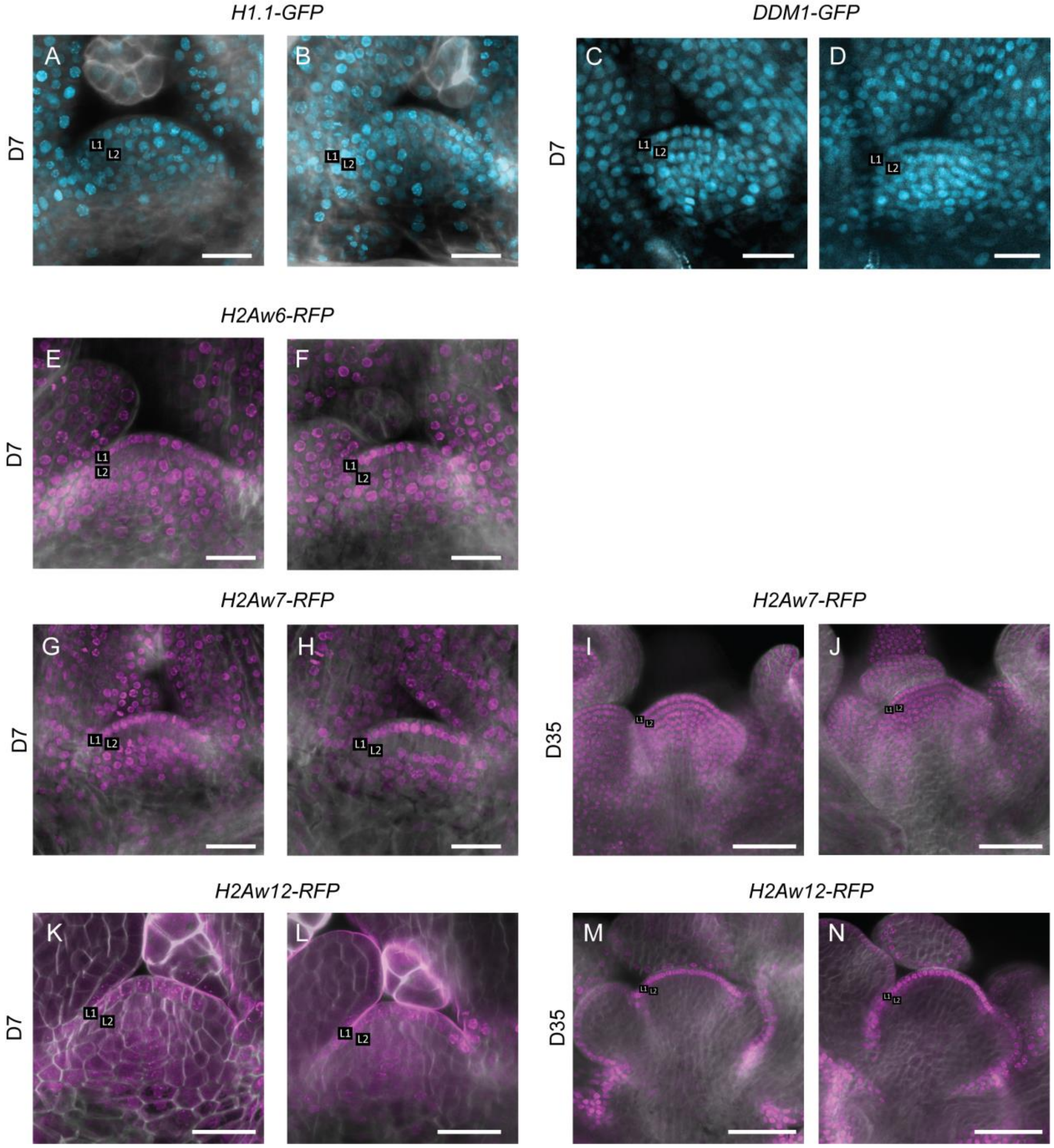

Supplemental Fig. S23 | Distribution of histone variants in SAM stem cells of D7 seedlings.

Two images of different meristems represent each reporter at either 7 d.a.g. (D7) or 35 d.a.g. (D35). **a,b,** Histone H1.1 labels heterochromatin (She et al. 2013). **c,d,** signal of the chromatin remodeler DDM1-GFP (Slotkin et al. 2009). **e,f,** reporter for H2A.W6. **g-j,** reporter for H2A.W7, **k-n,** reporter for H2A.W12. Reporters for H2A.W6, H2A.W7, and H2A.W12 are from (Yelagandula et al. 2014). L1 and L2 inserts label the epidermis (L1) and the sub-epidermis (L2). Scale bar **a-h,k,l** = 20 µm; **i,j,m,n** = 50 µm.

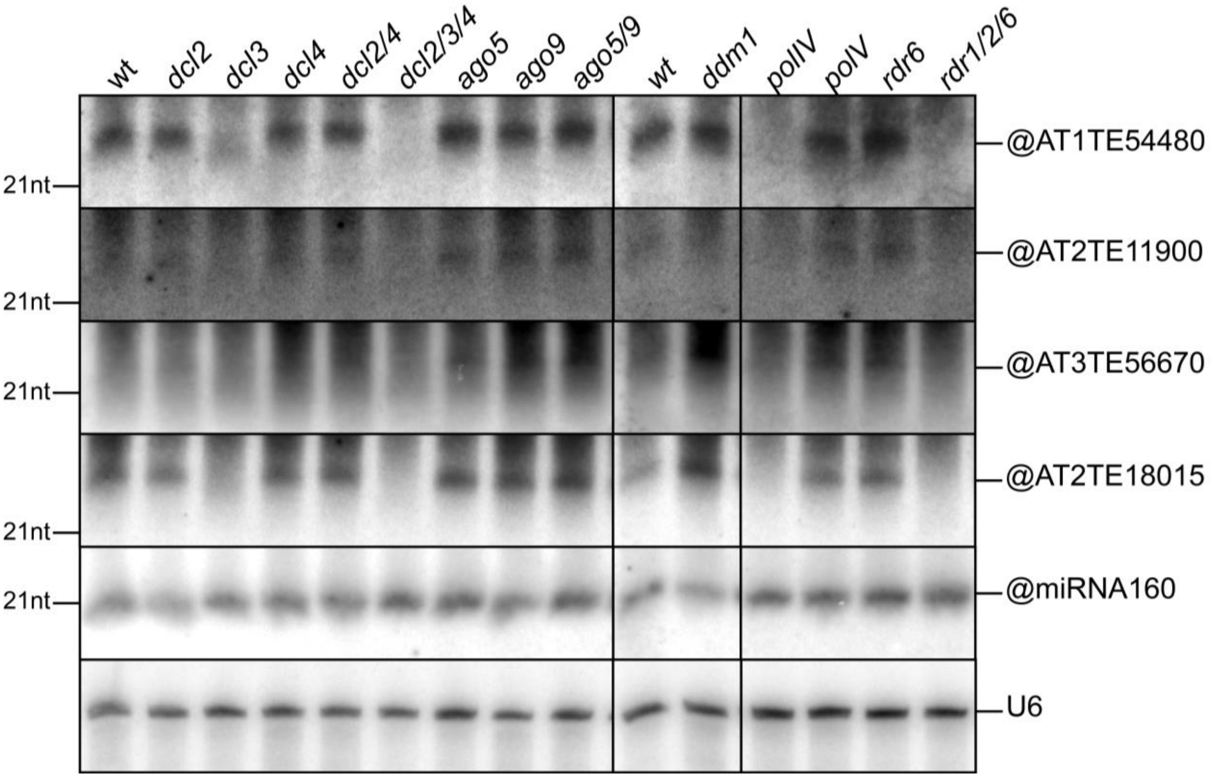

Supplemental Fig. S24 | siRNAs derived from TEs targeted by AGO5 are synthesized by PolIV, RDR2, and DCL3. RNA was extracted from apices of 7 day-old seedlings of the indicated genotypes. sRNAs from the indicated TEs show no or a weak signal in *dcl2*, *dcl2/3/4*, *polIV*, and *rdr1/2/6* mutants, but a visible signal in the size class from 22-24 nt in the other genotypes. miRNA160 and U6 are shown as loading controls.

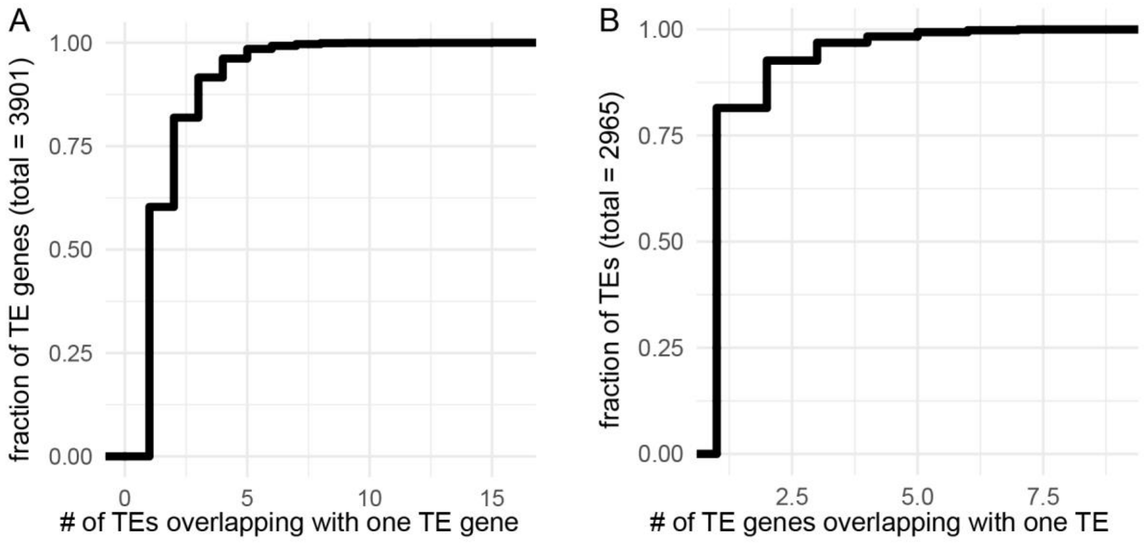

Supplemental Fig. S25 | Fraction of ambiguous TE annotations. Fraction of TE genes with more than one overlapping TEs (**a**) and the fraction of TEs with more than one overlapping TE genes (**b**). Supports Methods.

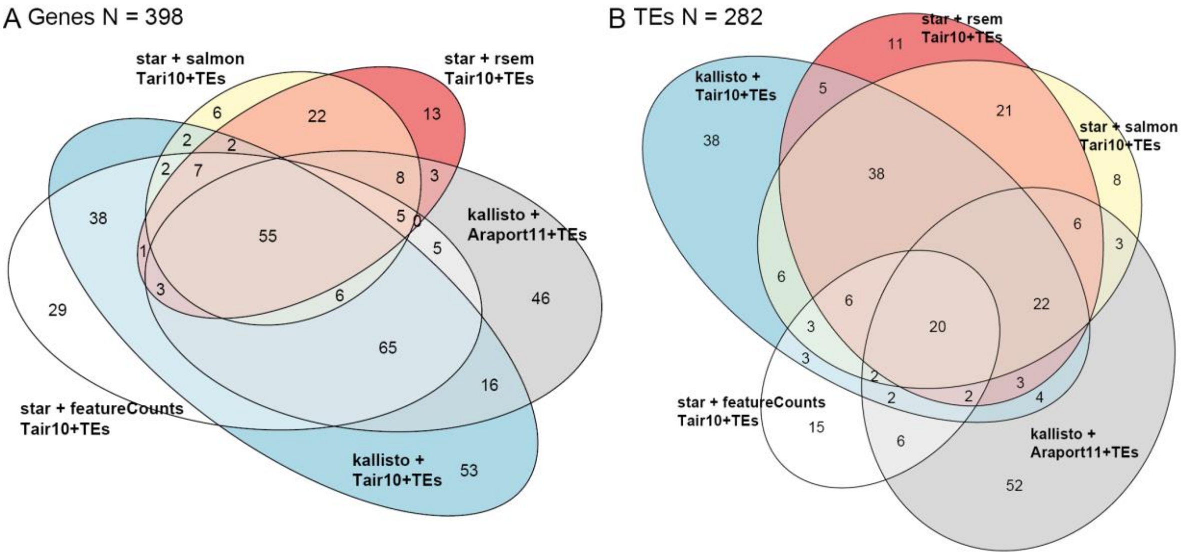

Supplemental Fig. S26 | Comparison of different alignment strategies. An example dataset was aligned with either star or kallisto and reads quantified with featureCounts, Salmon, or Kallisto. Shown is the overlap of DESeq2 differentially expressed features (FDR < 0.05) for each count table for genes (**a**) and transposons (**b**). Supports Methods

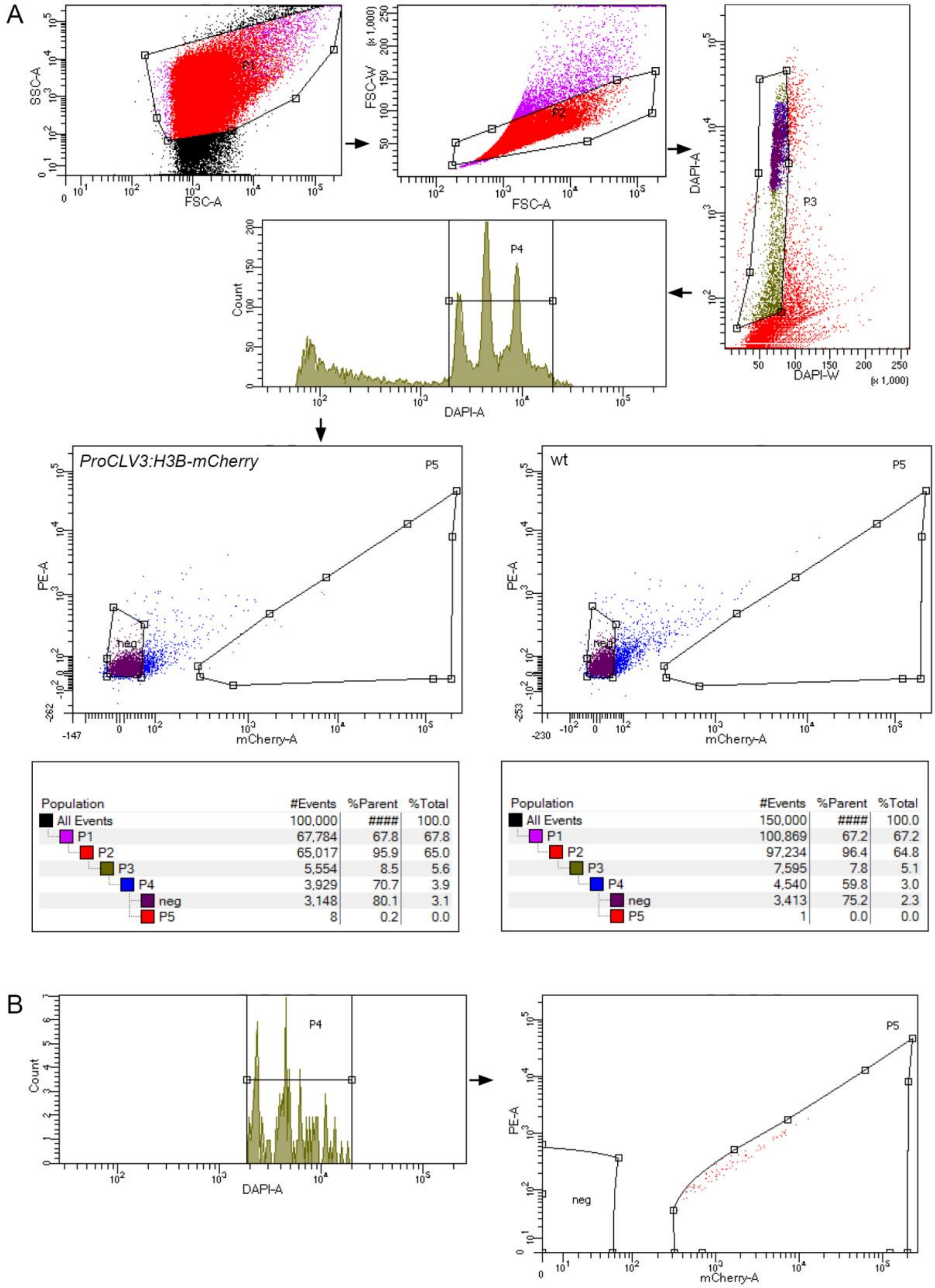

Population	#Events	%Parent	%Total
All Events	100,000	####	100.0
P1	67,784	67.8	67.8
P2	65,017	95.9	65.0
P3	5,554	8.5	5.6
P4	3,929	70.7	3.9
neg	3,148	80.1	3.1
P5	8	0.2	0.0

Population	#Events	%Parent	%Total
All Events	150,000	####	100.0
P1	100,869	67.2	67.2
P2	97,234	96.4	64.8
P3	7,595	7.8	5.1
P4	4,540	59.8	3.0
neg	3,413	75.2	2.3
P5	1	0.0	0.0

Supplemental Fig. S27 | Example of gating strategy for sorting of nuclei for snRNA-seq. a, Gating strategy including DAPI and mCherry gates. b, DAPI and mCherry gate for 85 individual nuclei sorted for sequencing. Supports Methods.

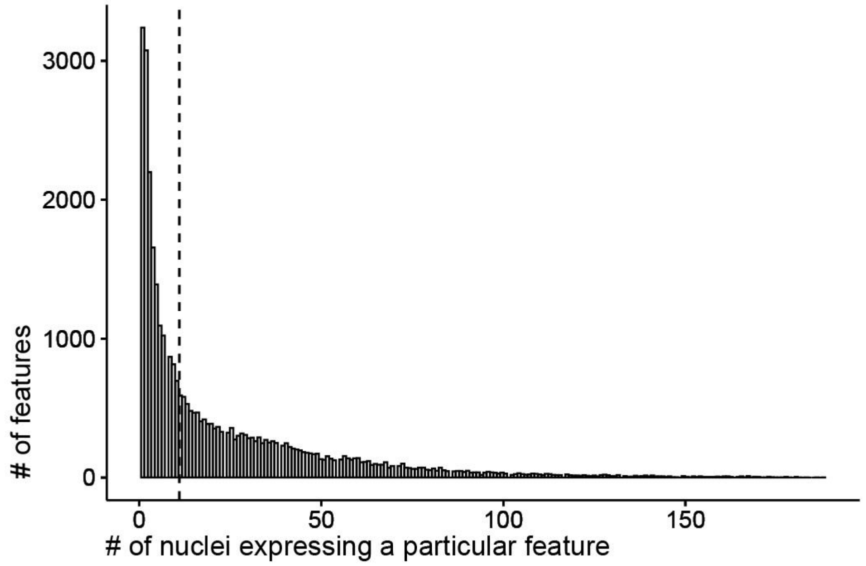

Supplemental Fig. S28 | Number of features expressed in number of nuclei. Supports Methods.

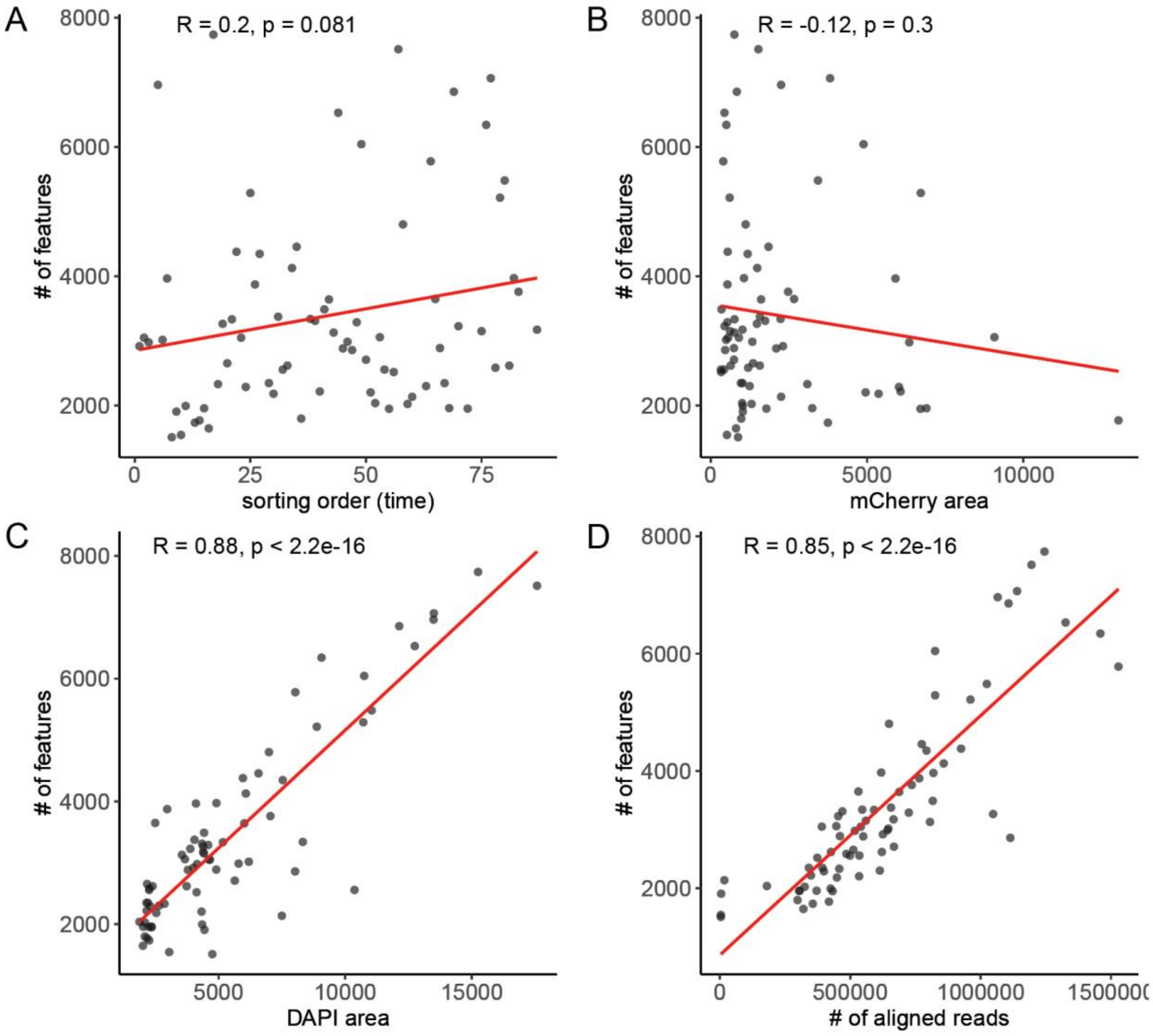

Supplemental Fig. S29 | Comparison of FACS vs snRNA-seq data. a, Scatterplots showing # of detected features (genes and TEs) over sorting order, **b,** mCherry signal, **c,** DAPI signal, and **d,** number of aligned reads. Supports Methods.

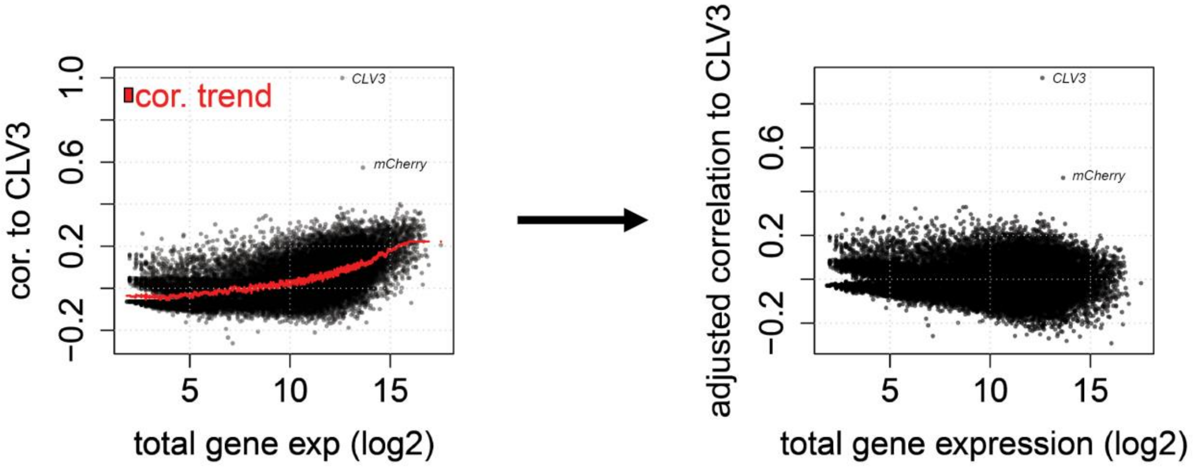

Supplemental Fig. S30 | Correlation and adjusted correlation to *CLV3*. Each dot resembles a gene (N= 24,761), and its raw Spearman's correlation (left) and adjusted correlation (right) to *CLV3* is depicted on the y-axis. Total expression levels (log_2 transformed) are shown on the x-axis.

References Supplemental material Buisine N, Quesneville H, Colot V. 2008. Improved detection and annotation of transposable elements in sequenced genomes using multiple reference sequence sets. *Genomics* **91**: 467-475.

Czechowski T, Stitt M, Altmann T, Udvardi MK, Scheible WR. 2005. Genome-wide identification and testing of superior reference genes for transcript normalization in Arabidopsis. *Plant Physiol* **139**: 5-17.

Lanciano S, Cristofari G. 2020. Measuring and interpreting transposable element expression. *Nat Rev Genet* **21**: 721-736.

Meir Z, Mukamel Z, Chomsky E, Lifshitz A, Tanay A. 2020. Single-cell analysis of clonal maintenance of transcriptional and epigenetic states in cancer cells. *Nat Genet* **52**: 709-718.

O'Neill K, Brocks D, Hammell MG. 2020. Mobile genomics: tools and techniques for tackling transposons. *Philos Trans R Soc Lond B Biol Sci* **375**: 20190345.

She W, Grimanelli D, Rutowicz K, Whitehead MW, Puzio M, Kotlinski M, Jerzmanowski A, Baroux C. 2013. Chromatin reprogramming during the somatic-to-reproductive cell fate transition in plants. *Development* **140**: 4008-4019.

Slotkin RK, Vaughn M, Borges F, Tanurdzic M, Becker JD, Feijo JA, Martienssen RA. 2009. Epigenetic reprogramming and small RNA silencing of transposable elements in pollen. *Cell* **136**: 461-472.

Yelagandula R, Stroud H, Holec S, Zhou K, Feng S, Zhong X, Muthurajan UM, Nie X, Kawashima T, Groth M et al. 2014. The histone variant H2A.W defines heterochromatin and promotes chromatin condensation in Arabidopsis. Cell 158: 98-109.
