# Supplementary material for: Two ARGONAUTE proteins loaded with transposon-derived small RNAs are associated with the reproductive cell lineage in Arabidopsis
Source: Plant Cell. 2023 Dec 7;36(4):863–80. doi: 10.1093/plcell/koad295 (PMC10980394; doi:10.1093/plcell/koad295)
Supplement: koad295_Supplementary_Data [file tpc23.00052.PeerReviewReport.pdf › tpc23.00052.PeerReviewReport.pdf]

Two AGO proteins loaded with transposon-derived sRNA are associated with the reproductive cell lineage throughout development in Arabidopsis

Gabriele Bradamante, Vu Hoang Nguyen, Marco Incarbone, Zohar Meir, Heinrich Bente, Mattia Donà, Nicole Lettner, Ortrun Mittelsten Scheid, Ruben Gutzat

Corresponding author: Ruben Gutzat, ruben.gutzat@gmi.oeaw.ac.at

Review timeline:

**TPC2023-RA-00052**	Submission received:	Jan. 13, 2023
	1st Decision:	Mar. 2, 2023 *revision requested*
**TPC2023-RA-00052R1**	1st Revision received:	July 29, 2023
	2nd Decision:	Sept. 14, 2023 *acceptance pending, sent to science editor*
	Final acceptance:	Nov. 23, 2023

REPORT: (The report shows the major requests for revision and author responses. Minor comments for revision and miscellaneous correspondence are not included. The original format may not be reflected in this compilation, but the reviewer comments and author responses are not edited, except to correct minor typographical or spelling errors that could be a source of ambiguity.)

TPC2023-RA-00052 1st Editorial decision – *revision requested* Mar. 2, 2023

We have received reviews of your manuscript entitled "Two AGO proteins with transposon-derived sRNA cargo mark the germ track in Arabidopsis." Thank you for submitting your best work to The Plant Cell. The editorial board agrees that the work you describe is substantive, falls within the scope of the journal, and may become acceptable for publication, pending revision and re-review. However I should stress that we are reluctant to see manuscripts undergoing multiple rounds of revision and would be unlikely to offer you more than one chance to satisfy the reviewers.

We ask you to pay attention to the following points in preparing your revision:

The "germ track" hypothesis is overstated throughout the manuscript and might be misleading for readers who are not highly familiar with reproductive development. This term should be removed from the title and ideally limited to the discussion section.

More data is needed to support the conclusion that TEs are expressed and enriched in AGO5/9 expression domains. Reviewers offer suggestions on approaches that might be helpful to gather this data.

There is an array of relevant literature in this area that is not cited and must be included. The authors should particularly note and discuss cases where their results differ from previous results.

-- Reviewer comments:

[Reviewer comments shown below along with author responses]

TPC2023-RA-00052R1 1st Revision received July 29, 2023

Reviewer comments and **author responses**:

Editors:

The "germ track" hypothesis is overstated throughout the manuscript and might be misleading for readers who are not highly familiar with reproductive development. This term should be removed from the title and ideally limited to the discussion section.

> **As suggested, we removed the term from the title and the Results to facilitate the reading of our manuscript for a broad audience. However, we still use it in the Introduction and Discussion and want to justify its usage here: the term "germ track" (Haig, 2016, Bioessays) was already a compromise and a placeholder for the original definition of "germline". It describes the cell lineage that has the potential - but is not limited to - develop into gametes, comprising, e.g., the zygote, the cells of the L2 of the shoot apical meristem (SAM), and the spore mother cells. This is exactly the cell lineage that August Weismann named the "Keimbahn" (which literally translates to "germline") (August Weismann 1892: "Das Keimplasma: eine Theorie der Vererbung" page 253-259"). Indeed, August Weismann created the term "germline" for the cell lineage reaching from the fertilized egg cell to cells that are potential contributors to the gametes. He pointed out that cells of the "Keimbahn" play a role in building the soma, while in plants, somatic cells can be part of the germline (page 259 in his publication). However, some**

authors argue that in plants, the term "germline" should be reserved for cells dedicated to developing into gametes, like the generative cell in developing pollen (Berger & Twell 2011). We notice this change in the understanding of the term "germline" but want to bring back a reference to the original definition by August Weismann in the context of our work.

More data is needed to support the conclusion that TEs are expressed and enriched in AGO5/9 expression domains. Reviewers offer suggestions on approaches that might be helpful to gather this data.

In our original manuscript, we had already identified high TE expression in the L2 of stem cells in one- week-old seedlings using two distinct approaches: single nuclear mRNA seq and AGO5 RIP-seq. We explain this in more detail in the direct response.

It is important to note that accessing the stem cells of the SAM (shoot apical meristem) and the generally low expression levels of most TEs pose significant challenges for several of the suggested experimental approaches. We appreciate the valuable comments provided by the referees; however, due to various reasons outlined in the direct response, most of their suggestions were not feasible to implement.

Despite these challenges, we have been able to generate new data on the chromatin configuration (see Supplemental Figure S23) and TE and gene expression in the L2 (Supplementary Figure S17 and S18). The distribution of heterochromatic histone variants presents supporting evidence for an altered chromatin configuration in the L2 of young seedlings. Due to technical constraints, the new L2 TE and gene expression data are derived from seedlings at the transition to flowering, as described in the detailed response. Nonetheless, this new dataset corroborates the dynamic TE expression pattern observed in agreement with our previous findings (Gutzat et al., 2020, EMBO J). This new expression data set will add valuable information to the research community (Supplemental Figure S17, S18 and Supplemental Table T2 and T3).

Please consider that our laboratory at the GMI is currently closed following the retirement of Ortrun Mittelsten Scheid. As a result, conducting follow-up experiments in a new location will take some time to arrange. However, in the meantime, we are committed to sharing our data with the scientific community, given the considerable interest generated by the preprint and multiple conference presentations of our work.

There is an array of relevant literature in this area that is not cited and must be included. The authors should particularly note and discuss cases where their results differ from previous results.

We apologize if we omitted any of the mentioned publications in our original manuscript. We now have included and discussed all of them. Thank you and the referees for pointing this out.

Please note that, for easier access, line numbers in the responses refer to the lines in the version with the marked changes.

<u>Reviewer #1:</u>

In this work, Bradamante and Nguyen et al., investigate the expression, loading and function of two Arabidopsis Argonaute proteins, AGO5 and AGO9, in the shoot apical meristem. These two AGOs are enriched in the stem cells of the SAM and at some developmental stages specifically found in the L2 layer. This finding is very interesting as the germ line, later in development, will derive from that same cell layer. The authors show that AGO5 and AGO9 in stem cells are loaded with sRNA mainly corresponding to transposable elements suggesting that those AGOs could be involved in the protection of the L2 layer against harmful reactivation of transposition. This paper presents novel and important results clearly interesting for the readership of Plant Cell.

We greatly thank the reviewer for the suggestions and constructive criticism and for appreciating our work.

However, the authors should modify their claim about the "germ track" and put their results in the context of other published data.

We followed the reviewer's suggestion and removed the term "germ track" from the title and the Results. We intended to say that both AGOs together mark the "germ track", meaning that at any time, at least one of those two proteins is present -though not exclusively- in cells of the germ track. However, we realized that it could be misunderstood as stating that both proteins are present exclusively in "germ track" cells, interpreting "and" in "AGO5 and AGO9" as a Boolean term. We agree that this would be an overstatement, and therefore we limited usage of the term "germ track" in connection with AGO5 and AGO9 to the Introduction and the Discussion.

We also added and extended the discussion of other published work in the field (as listed below).

Major

The SAM data are really interesting and definitely intriguing with the marking of the L2 layer by AGO5 and AGO9. It is therefore appealing to hypothesize a marking of the "germ track". However, the authors should tune down this concept of AGO9 and AGO5 marking the germ track overall in the manuscript, including the title, as it is not truly the case and misleading to the readers.

As answered above, we removed the term from the title and the Results.

1) Both AGO5 and AGO9 are clearly expressed in other tissues that are not belonging to the "germ track" for example SAM L1 (Fig1.b,e,s,t,w), nucleus (Fig. m,n,p,r), funiculus (Fig1p) and integuments (Fig 1p and 1r).

As answered above, we removed the term from the Results.

2) As the author mentioned the L2 will give rise to the germ line while the L1 will not, could the authors provide an hypothesis or discuss on why AGO5 and AGO9 seem to change their preference for L1-L2 or only L2 during plant development?

Thank you for the suggestions to discuss this in more detail. Although so far speculative, we assume that there are additional functions of AGO5 and AGO9 during development, especially upon flowering induction or stress signaling, likely related to regulatory roles of sRNA cargo of AGO5 or AGO9 in other tissues. We also cannot exclude that there is a feedback from TE activation on AGO5/9 expression. We have added this to the Discussion (lines 377-381).

AGO5 is L2 at D7, but L2-L1 at D35 and SD D21 while AGO9 is L1-L2 at D7 but L1 at D14 and SD D21. Could they be actually stem cell-specific but their L1/L2 expression ratio rather depending on environmental conditions such as seen with the SD experiment?

Certainly, we acknowledge the possibility raised by the reviewer. To investigate whether the spatial expression of AGO5 and AGO9 is influenced by the environment, we conducted heat stress experiments. However, our observations did not reveal any discernible difference in the localization of the reporter proteins in young seedlings (D7). (see the new Supplemental Figure S8).

Are there other genes behaving similarly in the SAM? Is this seen with other AGO5-AGO9 reporters?

We observed the same patterns with independent reporter lines (see the new Supplemental Figure S1). We are unaware of reports demonstrating a similar expression behavior of other genes with the same high temporal and spatial resolution, and we agree that more information for other genes should be collected in a similar way. But this goes beyond the scope of this manuscript.

3) In addition, as the author themselves mentioned, they see gaps of expression during the "germ track" developmental history, for example, AGO5 is not expressed in tetrads and MMC or AGO9 in the FM and IFM. All are arguments against a real marking of the "germ track" throughout development.

Additionally, to the expression pattern, the omics experiment from this paper mostly concerns the SAM CLV3/AGO5/AGO9 expression domain and mature sperm cells, which is not the multitude of cell types that correspond to the "germ track". The authors should heavily tune down or remove the "germ track" notion overall in the manuscript and title. They should replace it with "L2 layer" and develop the "germ track" hypothesis rather in the discussion.

Please see our comment above: we apologize for the misunderstanding. We removed the term from the title and the Results and comment on the germ track issue in the Introduction and Discussion.

For both expression pattern and sRNA IP, the authors should better cite, compare and discuss what is different or not from previously published work.

AGO5 expression pattern in germ line was already described previously and differences can be seen for cell types such as for the nucellus, integuments and hypophyses (1-5). Similarly, AGO9 expression pattern germ line was also thoroughly looked at and debated (2,5-7).

The authors mentioned the AGO5 IP obtained by Mi et al in-culture cells but do not mention if they find similar types of sRNA or not. The authors should mention, compare, and discuss the previously published AGO9 IP in inflorescences. (6,8).

Thank you for these suggestions and we sincerely apologize for missing to mention previous important work. We have now included all references and discussed them in the respective context.

We included (1) and (2) in line 120 and (2) and (5) in line 141. We have included (3) in line 360 and in line 437. (4) is included in line 127 and 441.

We would like to highlight that the variations in reporter signal could be attributed to differences in reporter construction methodologies. For instance, in (2), transcriptional fusions were employed, while in (5), translational AGO9-GFP fusions were generated using a promoter fragment 1.2 kb shorter than that in our study.

We attempted to perform the suggested comparison with the AGO5 IP data from (8). However, we encountered challenges as the fastq files from (8) back in 2008 are no longer compatible with the current alignment tools, such as bowtie.2 and bwa. Further, they contain mainly sRNA from intergenic regions, which at that time were not yet annotated. The 5' bias of their sRNAs is similar to that in our data, and we mention this in line 186.

The data from (6) were generated from fully grown aerial Arabidopsis plants, which also limits a direct comparison. Nonetheless, (6) was the first to describe the 5' bias for AGO9 associated small RNAs, and we sincerely apologize for not acknowledging this work earlier. We have now included a citation in our manuscript (Line 188).

The data for AGO9 cargo from (7) originate from Sanger sequencing and are available only in pdf format, making a comparison difficult, but we quote their work in line 162.

The paper focus on an RdDM view of the sRNA-AGO function. However, there is a lot of miRNA function occurring in the SAM. Could the authors comment or discuss on how their data could interfere with those? Do they also see miRNA loaded in AGO5 or AGO9? Especially as a function of AGO5 in the SAM was linked to this pathway via miRNA-SPL module and should be mentioned in the manuscript 9 .

We show all bound miRNAs in Supplemental Table S4. We discuss and hypothesize miRNA function in the context of easiRNAs, in paragraph 2.5 and the Discussion. In the AGO9 cargo, we found miR829 (D7) and miR839 (D35), both without known functions. As AGO9 mainly binds to 24-mer, and not to 21 nt small RNAs, we would expect a minor role for this association.

As the *ago5* mutant shows no developmental phenotypes in our growth conditions, we speculate in the manuscript that its primary function is transposon repression via DNA methylation or the easiRNA pathway, in agreement with (3).

Indeed, we found miR156 in the AGO5 IP in flowering plants (at D35), in agreement with (9), but we did not observe the described early flowering phenotype in the two knock-out *ago5* alleles we used. Differences in light quality or intensity during culture might explain this. However, we could not find miR156 binding to AGO5 in young seedlings, and we would like to caution against interpreting the functionality of miRNA binding with a 5' uridine. For example, we could observe that the binding of AGO5 to mir164 is not to the guide strand (with a 5' uridine) but to the passenger strand (with a 5' cytosine), which complicates the interpretation of the data. The presence of miRNAs with a 5' uridine in AGO5 precipitate might also result from AGO1 contamination (explained below).

Nevertheless, we have included the reference now in the Discussion (Lines 426-428).

Minor

I could not find which alleles were used for which experiments. The authors should add the allele's names throughout the manuscript and figures.

All mutants used in our study are listed in Supplemental Table S5, including allele number, NASC code, source, and reference. Using the complete allele names throughout the manuscript and figures, especially in case of the double mutants, would reduce the reading flow. Therefore, we would like to stay with the short names in the main manuscript.

It is not clear how many independent transgenic lines were checked including for SAM expression pattern.

Initially, we evaluated many independent lines at the stage of young seedlings, without detecting noticeable differences. Therefore, we focused on one main line for the majority of the work.

However, we now included Supplemental Figure S1 showing the localization of the AGO reporters in a second, independent line each.

The figure referencing is not consistent in the text sometimes 2a or 2a '

We apologize for the inconsistency. We corrected wrong references.

It seems that the expression patterns observed by the authors are narrower than previously published, could it be due to the fixation? Did the authors observe the same pattern on live tissues?

As it was not specified which data the reviewer is referring to, we cannot answer directly. Please note that fixing the tissue is necessary to liberate and visualize intact meristems in the vegetative growth stage in young seedlings. We applied only a mild and short fixation with formaldehyde. However, to address the concern, we now provide unfixed samples of inflorescence meristems (Supplemental Figure S7), which look precisely like the fixed respective samples.

It is not clear why the AGO5 IP is contaminated with AGO1 while the authors use GFP beads. Was the interaction of AGO1 with GFP beads previously documented? Why is it not happening in other AGO IPs?

We apologize for not discussing this in more detail. AGO5 abundance is very low in seedlings and restricted to only a few cells, while AGO1 is very high, as seen by the strong AGO1 band in the input material (excised shoot apices of 7 days-old seedlings, Supplemental Figure S11a, notice the strong band in the input (I) using anti-AGO1). Without the preceding removal of AGO1, and even after several washing steps of the precipitating beads, we still found a considerable amount of AGO1 in the bead exclusion volume (see the AGO2 band in the AGO5 precipitate (P)). In addition, AGO1 seems to be especially "sticky" compared to other AGO proteins and was found enriched during immune precipitation also by others. Whether this is only a consequence of the high abundance of AGO1 or due to structural or biophysical properties of AGO1 goes beyond our study.

The PCA analysis from fig2c shows the high similarity between the D7 samples. Is it due to a high background at this stage? How does the PCA look like without D35?

The differences in length distribution profiles and 5' base preferences of the loaded sRNAs confirm the specificity of the cargo and exclude high background of small RNAs in the precipitate. In accordance with the reviewer's suggestion, we generated the PCA without D35 (see Figure below), which validates the findings depicted in Figure 2. Furthermore, it highlights a strong resemblance in the putative AGO5 and AGO9 targeted regions. As there is no significant difference to the PCA in Figure 2c, we show the PCA without D35 only here. The much bigger differences between AGO5 and AGO9 in the D35 samples are confirmed by different TE superfamilies represented in the cargo.

PCA analysis of the small RNA data without D35:

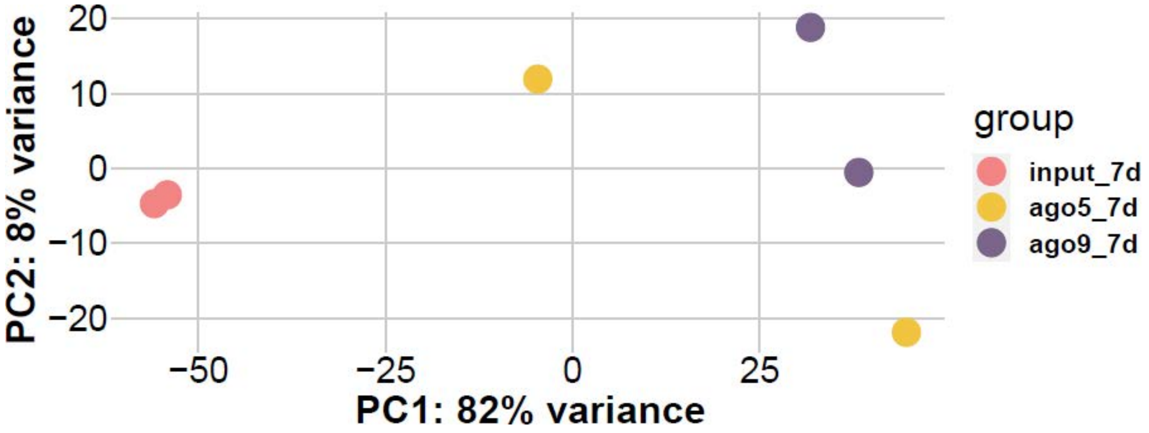

Could the authors discuss more about the function of other AGOs in the meristem? Are there other AGOs in the L2?

We concur with the reviewer regarding the value of a comprehensive investigation into the functions of all AGOs throughout development, particularly at the same spatial and temporal resolution as demonstrated in our study for AGO5 and AGO9. However, undertaking such an endeavor for these revisions would exceed the scope of our current manuscript. However, we agree with the reviewer that we could have better discussed known functions of AGOs in the meristem. Previous research has indicated the function of AGO10 as a sponge for mir166. Notably, one *ago10* mutant exhibited a strong developmental phenotype, however, only in a specific genetic background (Ler accession). We have included this in the Discussion in lines 419-423 (Zhu et al., 2011 and Zhou et al., 2015). AGO6 has been previously established as a crucial player in de novo transcriptional silencing, as demonstrated in studies conducted by Eun et al. (2011) and McCue et al. (2015), which we have included in the Discussion (lines 424-426).

We also added our newly generated transcript data of all AGOs in the SAM (Supplemental Figure S18), which shows high expression also of AGO1, AGO10, and AGO4 in all SAM stem cells and not limited to the L2 like AGO9 at this time point.

In sup Fig2, the drawing is wrong for AGO9 expression pattern in MMC and FM. AGO2 expression is not limited to the FM and MMC.

We have corrected this.

The authors do not see AGO5 and AGO9 transcripts in what I assume are mature sperm cells and they hypothesize a carryover from the microspore. However, at least for AGO5 they do not see it at the tetrad stage. Could it be simply that in mature sperm cell, those genes are not transcribed but the protein is stable compared to immature sperm cells or generative cells where those genes would be transcribed and protein produced?

We apologize for the misunderstanding. As depicted in Supplemental Figure S21c, our data show that AGO transcripts in sperm cell nuclei are ONLY found for AGO5 and AGO9.

Nevertheless, it is worth noting that prior research (Borges et al., 2018 and Oliver et al., 2022) has documented AGO1 activity in sperm cells. To reconcile this discrepancy, we postulate that the AGO1 activity detected in sperm cells may originate from carryover via transfer from the microspore mother cell. To address this point more explicitly, we have incorporated an additional sentence into the Discussion (lines 435-438) of our manuscript.

The authors suggest a particular chromatin state of the L2. As they performed DAPI staining of SAM nuclei during their imaging, could the authors observe or measure chromocenter organization of the L2 versus other cells of the SAM?

We could not observe obvious differences in DAPI staining in SAM stem cell nuclei. We also looked at the heterochromatic histone H1.1 (new Supplemental Figure S23a,b) and could not identify notable differences.

However, following the reviewer's suggestion (see also below), we have added images showing the distribution of other histone variants, indicative of different chromatin states in the SAM (Supplemental Figure S23). This indeed suggests that the SAM layers differ in the amount of histone variants characteristic for repressive chromatin (see images for H2A.W6 and H2A.W7, Supplementary Figure S23 e-h). This has been added to the text (lines 343-349 and lines 403-405).

In Fig1f-The picture of GFP-AGO9 in the IFM is not clear. We cannot see the GFP-AGO9 signal but we mainly see a strong green fluorescence below the IFM. Could the authors comment on that and maybe provide unmerge channels in supplemental data.

We now provide images prior to merging in Supplemental Figure S5. Additionally, we present corresponding images without the GFP-AGO9 reporter, revealing strong autofluorescence in the tissue below the meristem.

The authors do not observe changes in DNA methylation in the stem cells of ago5 and ago9 mutants, could these AGOs rather act to recruit histone modifiers as suggested by parent et al 10? Did the authors try to look at histone marks changes in the SAM by IF? Were histone marks checked in the SAM previously?

We agree that distribution of histone variants in SAM stem cells and effects of the AGO mutations on histone modifications, in combination with or independent from DNA methylation, are the next features to study. So far, there is limited information. The only genome-wide study that we are aware of investigated H3K27me3, H3K4me3 and H3K9me2 in the whole SAM, but with insufficient resolution to distinguish the stem cells. We now cited this work in the Discussion, lines 406-409.

As answered above, we have now added images showing the distribution of repressive histone variants (Supplemental Figure S23) and discuss the results (lines 343-349 and lines 403-405).

The authors hint at a direct role of AGO5 in DNA methylation. Does their NESmutant complement the reduction of DNA methylation observed in the sperm cell?

This could indeed be an interesting continuation to study the role of AGO5, but it would address only the possible role for DNA methylation as a cytoplasmic protein. It would require more controls and constructs, and we believe that this goes beyond the scope of this manuscript.

The authors do not see DNA methylation changes in ago9 mutant in both stem cell and sperm cell. Is it overall or only on TE? Which allele is used? Is it a KO? Could there be sequence difference between AGO4 and AGO9 interfering with its role in Rddm?

For our study, we employed a previously characterized knock-out allele for *ago9*, as indicated in Supplemental Table S5. Supplemental Figure 19 demonstrates that there are no discernible differences in DNA methylation patterns on genes or transposable elements (TEs) when comparing *ago9* to the wild type. The absence of DNA methylation changes, particularly in stem cells, could potentially be attributed to redundancy with AGO4 and AGO6, given that both are also expressed in SAM stem cells (Supplemental Figure S18 and S21c). Although our transcript analysis reveals only AGO5 and AGO9 to be expressed in sperm cells, we cannot exclude the possibility of AGO4 or AGO6 protein carry-over. Naturally, the sequence disparity between AGO4 and AGO9 may also imply distinct molecular functions.

Tune down: "The preferential loading of TE-related sRNAs implies that both, AGO5 and AGO9, are TE-silencing factors in the Arabidopsis germ track throughout. »

As described above, we removed the term "germ track".

Line 211 "AND" in capitalize letter

Now line 251, we wanted to emphasize AND as Boolean operator. References:

Borges, F., Pereira, P. a, Slotkin, R. K., Martienssen, R. a & Becker, J. D. MicroRNA activity in the Arabidopsis male germline. J Exp Bot 62, 1611-20 (2011).

Sprunck, S. et al. Elucidating small RNA pathways in Arabidopsis thaliana egg cells. bioRxiv (2019) doi:http://dx.doi.org/10.1101/525956.

Oliver, C. et al. The miRNome function transitions from regulating developmental genes to transposable elements during pollen maturation. Plant Cell 34, 784-801 (2022).

Tucker, M. R. et al. Somatic small RNA pathways promote the mitotic events of megagametogenesis during female reproductive development in Arabidopsis. Development 139, 1399-1404 (2012).

Jullien, P. E., Schröder, J. A., Bonnet, D. M. v, Pumplin, N. & Voinnet, O. Asymmetric expression of Argonautes in reproductive tissues. Plant Physiol kiab474 (2021) doi:10.1093/plphys/kiab474.

Havecker, E. R. et al. The Arabidopsis RNA-directed DNA methylation argonautes functionally diverge based on their expression and interaction with target loci. Plant Cell 22, 321-34 (2010).

Olmedo-Monfil, V. et al. Control of female gamete formation by a small RNA pathway in Arabidopsis. Nature 464, 628-632 (2010

Mi, S. et al. Sorting of small RNAs into Arabidopsis argonaute complexes is directed by the 5' terminal nucleotide. Cell 133, 116-27 (2008).

Roussin-Léveillée, C., Silva-Martins, G. & Moffett, P. ARGONAUTE5 Represses Age-Dependent Induction of Flowering through Physical and Functional Interaction with miR156 in Arabidopsis. Plant Cell Physiol 61, 957-966 (2020).

Parent, J. S., Cahn, J., Herridge, R. P., Grimanelli, D. & Martienssen, R. A. Small RNAs guide histone methylation in Arabidopsis embryos. Genes Dev 38, 1-6 (2021).

Reviewer #2:

In this manuscript, Bradamante and colleagues report that two Argonaute proteins (AGO5 and AGO9) mark cells associated with sexual reproduction, or germlines, throughout Arabidopsis development. Further, they found that AGO5/9 proteins are loaded with sRNAs that derive from highly methylated and long transposons located in pericentromeric domains of the genome. Because transposons are co-expressed with AGO5/9 in the SAM, they suggest that this supports the existence of an early segregating germline in plants.

The authors have performed an impressive series of difficult experiments and these datasets could be valuable to the research community.

This research topic is also of high interest in the field currently making this report is timely. Most of the data presented in the paper appear solid and the authors have described such complex topics and techniques well.

We would like to thank the reviewer for critically reading our manuscript, for the constructive suggestions on how to improve it, and for appreciating the challenge inherent to many of the experiments.

That said, the main conclusions of this paper rely on evidence that transposons are expressed in the AGO5/9 domain. Below, I have made suggestions on how this hypothesis could be tested further and other comments that may improve this manuscript.

Major comments

The authors claim that thousands of transposons are expressed and are enriched in AGO5/9 expression domains. This is an important claim for the authors' conclusions, but the data in Fig. 3 is not convincing. Typically, transposon transcripts are barely or undetectable in WT Arabidopsis plants with standard RNA-seq and so it is surprising that they were able to detect these with the much less sensitive single-cell RNA-seq approach. The average number of normalized counts appear extremely low and it is difficult to make firm conclusions based on such sparse data. Could these effects be strengthened by using uniquely mapping mRNA-seq reads? Or is there another comparison with available datasets that can be used to test this? Otherwise, I think fluorescent reporters, RNA in situ (or qPCR) are needed to substantiate this claim.

We want to highlight that our assertion of high TE expression in the L2 of SAM stem cells is not solely based on Figure 3. We used an orthogonal approach involving the precipitation of L2-specific AGO5 and subsequent sequencing of its associated small RNAs. This revealed a substantial enrichment of small RNAs originating from the same TEs that we observed to be expressed in the L2 using the single nuclear sequencing method. This combined evidence bolstered our confidence in claiming high L2- specific TE expression.

Furthermore, we would like to comment on the RNAseq data analysis. We present evidence supporting the enrichment of the expression of hundreds of transposons in the AGO5/9 expression domain. However, it is

important to note that we have observed reads corresponding to around 3700 transposons in the complete single nuclei sequencing dataset. We agree that transposon expression in somatic tissue is usually very low in wt. However, the detectability of rare transcripts can be significantly influenced by the sequencing method employed. For example, we have sequenced low input samples with smartseq2 and Trueseq (see figure below). This revealed that Trueseq libraries can suffer from low read complexity, especially on low abundant genes and transposons.

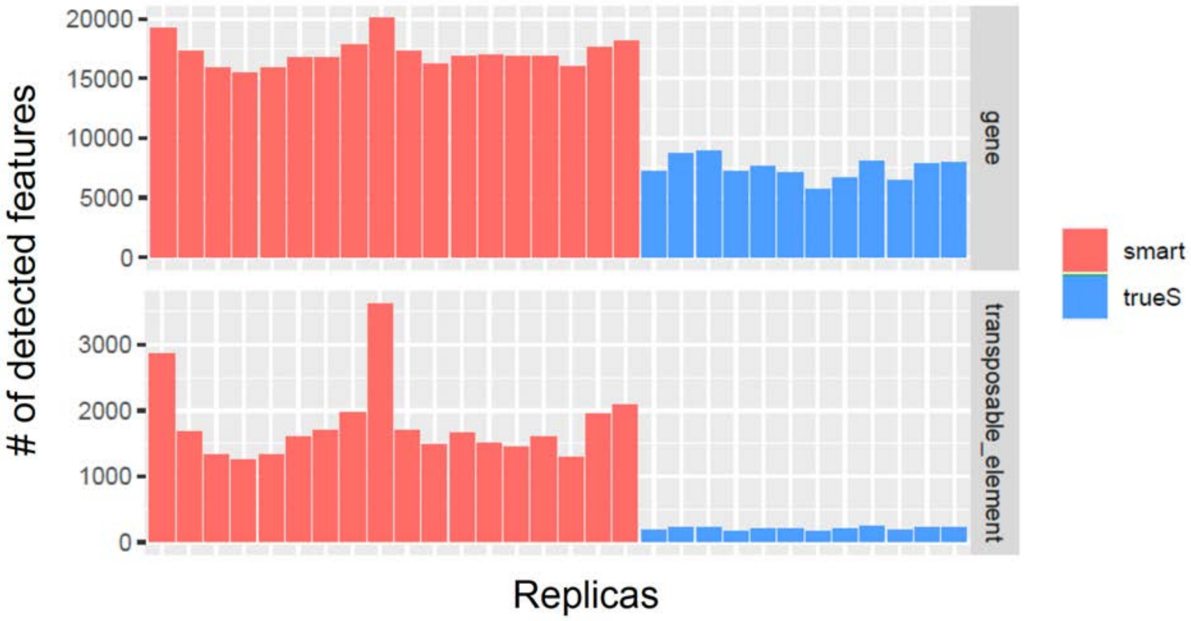

Replicas

We have outlined our data analysis methods in great detail in the Supplemental Methods and we were very careful to use the best available analysis methods. We used STAR for alignment, as it has been recommended for TEs (Kathryn O'Neill et al., 2019, Philos Trans R Soc Lond B Biol Sci.). As the reviewer correctly points out, single-cell omics data typically exhibit greater sparsity and lower complexity, making it crucial to reliably estimate transcript abundance. For this purpose, we employed Salmon, known for its superior performance in quantifying transcript expression (Patro et al., 2017, Nature Methods). Supplemental Figure S26 demonstrates that this mapping and quantification strategy is the most conservative, yielding the least "private" detected genes or transposons. In addition, community-based open-source tools like the next-flow RNAseq pipeline we have used are essential to augment reliability and reproducibility.

We expected that rare transcripts are better represented if data from 100 single sequenced nuclei are merged, as compared to bulk sequencing of 100 nuclei. While the average number of reads may appear low in Figure 3e (e.g., around 1 in AGO5/AGO9 nuclei), considering the total number of nuclei -24-, an average of 1 read corresponds to approximately 24 reads in the data set.

We appreciate the suggestions for additional experiments. However, generating fluorescent reporter lines for transposons is difficult, and we are unaware of any case developed for Arabidopsis transposons. Existing reporters utilizing the LTR do not correspond to the accurate presence of the TE, as exemplified by the COPIA93 LTR-retrotransposon EVADE (compare Figure 1k from Mari- Ordonez et al., 2013 Nature Genetics, showing a distinct presence of EVADE in the L2 of *met1* epiRILs plants with Figure 1B and 1D from Zervudacki et al., 2018 EMBO J, showing LTR:GUS signal of the same transposon in leaf vasculature). Generating a translational fusion between a reporter gene and a coding region of the TE is difficult as TE proteins are processed by a protease, with an unknown cutting-site preference, from a polyprotein derived from a polycistronic mRNA. We believe succeeding in producing a translational reporter would go beyond the scope of the manuscript.

Indeed, we also tried using *in situ* hybridization, with a CLV3 probe as positive control. This resulted in only a very weak signal in 7-day-old seedlings (see picture below). Considering the much lower expression levels of most transposons compared to CLV3 (in our data sets at least 10x lower), *in situ* hybridization with TE probes would have

a low likelihood of success.

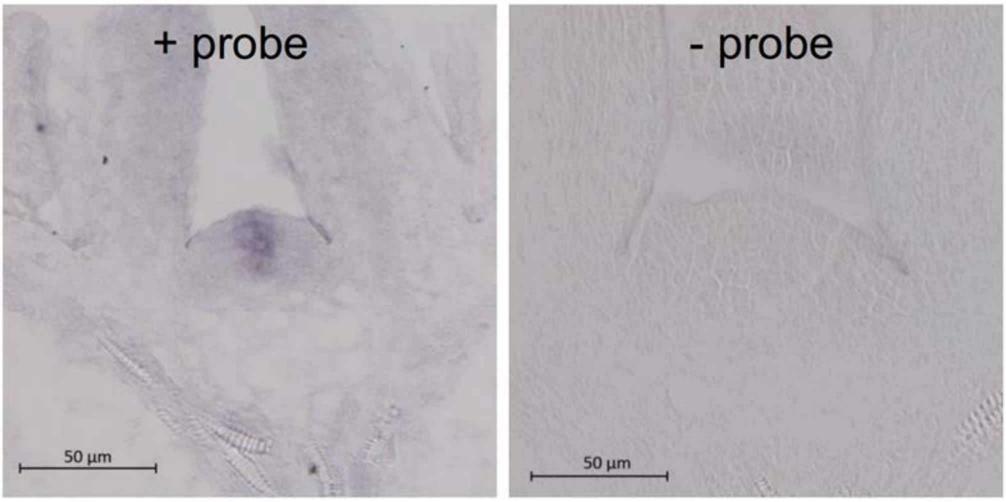

***In situ* hybridization for CLV3 in 7 day old seedlings.**

In addition to the single-nuclei mRNA-seq data, we wanted to test our hypothesis of L2 heterogeneity by bulk-sorting L2-specific nuclei and comparing their TE expression state to nuclei derived from other SAM stem cells or other meristematic cells. As nuclear AGO9 is confined to the L2 layer in older vegetative meristems in plants grown in short day conditions (Fig. 1x), we used AGO9-GFP signal in combination with the SAM marker CLV3-H2BmCherry as a reporter to sort L2 nuclei. This required a mild fixation (now added to Methods line 555-565). We sorted L2 nuclei from the center of the SAM (CLV3 (red) + AGO9 (green) + DAPI (blue), L2 nuclei from the periphery (green + blue), L1 and L3 nuclei (red + blue), and blue-only. Furthermore, we examined samples 2 days later after inducing flowering by shifting the plants to long day growth conditions (Supplemental Figure S17 and paragraph 2.3 lines 257-271 and Material and Methods section lines 555-565).

Intriguingly, our analysis revealed dynamic TE expression patterns in stem cells (Supplemental Figure S17d), consistent with findings in (Gutzat et al., 2020 EMBO J), wherein TE expression was high in young seedlings, but decreased in older seedlings. These findings suggest the possibility of a spike in TE expression during developmental transitions (from juvenile to adult, and vegetative to reproductive stages), or it may indicate that the increased TE expression observed in young seedlings contributes to TE silencing in older seedlings.

Although, for reasons explained above, we cannot analyze sequence-specific chromatin changes in parallel, distribution of the heterochromatin-specific H2A.W variants (included in Supplemental Figure S23 and paragraph 2.5 lines 343-349) revealed a distinct chromatin state in the L2 of D7 seedlings, which may potentially be associated with the observed high TE expression at this developmental stage and encourage future work in this direction.

Additionally, our data reveals unique L2-specific gene expression patterns, e.g., shedding light on the expression of cell cycle regulators (Supplemental Table S2). This finding is of great interest to the research community, as the slower cell division cycle in stem cells remains to be understood. We firmly believe that this dataset holds immense significance for the scientific community, especially for researchers investigating flower initiation and SAM stem cell biology.

Elaborate why evolutionary theory predicts that the germ track should have active transposons (line 55). For most readers, it may seem counterintuitive for the germline to express mutagenic TEs. And how the existence of transposon expression supports the existence of an early segregating germline should be explained.

This seemingly counterintuitive view is discussed in the literature and assumed to be due to the fact that the evolutionary success of transposons hinges on their ability to proliferate in host cells that guarantee vertical transmission from parent to offspring. In somatic cells, new insertions may pose a risk to host fitness without the benefit of being propagated to subsequent generations, whereas prevalence in cells that contribute to the germline ensures the continued propagation of transposons (for more information, see Haig 2016, Bioassays). To provide further clarity on this concept, we have extended the Introduction and cited the referenced article accordingly (lines

60-67).

Minor comments

Although the authors have done an outstanding job describing AGO5/9 expression domains in fine detail, AGO5 and/or AGO9 expression patterns were previously reported to be expressed in the SAM and germline by a couple other groups (e.g. Havecker et al. 2010, Tucker et al. 2012 (see Supplemental Figure 3), Jullien et al. 2021), and these papers should be cited. A similar comment can made about the previously reported AGO5 and AGO9-bound sRNAs.

We apologize if we did not include all references or did not discuss them in more detail. We have corrected this now (see also comments to Reviewer 1).

Instead of BLAST+, I recommend the authors to use plant sRNA target prediction tools that are commonly used.

We followed the recommendation of the reviewer and now also used another tool (psTARGET), in general resulting in higher numbers of target prediction than with BLAST+. We added this to Supplemental Table S4.

Line 50: In my opinion, it would be more clear to use the term "germline" rather than "germline sensu latu" or "germ track". I also disagree that plant germlines or "germ tracks" are synonymous with Weismann's "Keimbahn" described for animals.

Please see our general comments to this question above.

Line 129: It was surprising that 17.3% of WT ovule primordia produce >1 MMC. The authors should mention how this compares with other studies to help readers evaluate these results.

Given that we utilized the same allele as in the previous study by Olmedo-Monfil et al. 2010, our observation leads us to attribute the phenotype variation primarily to differences in growth conditions. Factors such as humidity, light spectrum, or intensity are likely contributing to the differences.

<u>Reviewer #3:</u>

Bradamante et al. claim according to the title of their manuscript that 'two AGO proteins with transposon-derived sRNA cargo mark the germ track in Arabidopsis' and further conclude that they demonstrated the existence of an early segregating germline in plants. This finding would represent a breakthrough in plant biology.

According to the famous Weismann's germline or germ track theory (Weismann, 1893), it was postulated that genetic determinants in egg cells/zygotes are inherited to the next generation by cells known as the germline and which after meiosis generate the gametes of a new organism. While the original concept considered a continuity of the germline and a 'Weismann barrier' between germline and somatic cells that was found in some animal species, most animals were later described to contain a discontinuous germline and germline determination occurs via epigenesis during (early) embryo development by the formation of primordial germ cells. However, molecular components that are expressed in egg cells and zygotes as well as other cells of the germline were shown to be also expressed in multipotent somatic cells, which led to the debate whether such cells named as primordial stem cells close the circle of germline-egg cell/zygote-germline via a subgroup of stem cells. In contrast to animals, it is thought that an embryonic germline or germ track does not exist in flowering plants and the germlines are not established before flowering.

We appreciate this recapitulation of the original “germline” definition; please see our comment on this topic in the common points.

Bradamante et al. now suggest that AGO5 and AGO9 of Arabidopsis involved in PTGS and RdDM pathways, respectively, represent molecular components that mark the plant germline/germ track throughout development. By showing beautiful microscopic imaging they detected cytosolic AGO5 in sperm cells and egg cells/zygotes, the apical cell after zygote division, the L2-layer of late globular stage embryos, L1+L2 layer of inflorescence meristems and microspore mother cells. It should be noted that AGO5 is not expressed in tetrads and microspores, and it is unclear

whether it is expressed in male generative cells. It is also not expressed in megaspore mother cells and its expression in developing female gametophytes was not investigated. AGO9 shows a similar pattern, but it is generally also expressed in differentiated cells of the ovule, somatic cells of axillary buds and lacks in their stem cells as well as in inflorescence meristems. In contrast to AGO5 it is present in megaspore mother cells and microspores.

Part of above information is not novel as Jullien et al. (2022) analysed the expression pattern of all 10 AGOs from Arabidopsis and found that most AGOs are expressed in male and female gametes as well as in zygotes/early embryos. AGO5/7/9 were also shown to be specifically enriched in the SAM area at the heart stage embryo and that both, AGO5 and AGO9 are also expressed in various differentiated somatic cells.

We have discussed similarities and differences with this previous work now more prominently, please see also comments to Reviewer 1 and 2.

Next authors analyzed shoot apices of D7 seedlings and compared these to those from mature plants (D35) containing inflorescence meristems, floral meristems, and very young flower buds. They could show that AGO5 cargo consists of 21, 22, and 24 nt sRNAs, while AGO9 cargo mainly consist of 24 nt sRNAs; sRNAs from both AGOs are mainly derived from different types of transposons. Furthermore, after FACS-sorting of stem cells from D7 seedlings, authors analyzed transcriptomes of 188 individual SAM stem cell nuclei and performed DNA methylation analysis involving also single and double ago5/9 mutants.

Overall, the manuscript is well written. Five figures are supplemented with 23 suppl. figures. There is a lot of novelty in the manuscript, and it deepens our understanding about AGO5/9 functions. However, parts appear incomplete, and the major conclusion seems to be an overstatement.

We thank the reviewer for her/his assessment and for all constructive suggestions for improvement.

MAJOR

1) In conclusion, the described expression pattern is clearly associated with reproduction and development, but both genes do not fulfill the concept of molecular components that are continuously transmitted via a germ track as postulated by the authors. The 'germ track' is interrupted multiple times, the genes are also expressed in differentiated somatic cells, thus in my opinion the major conclusion of the manuscript cannot be maintained. I am also very surprised, that this issue is not at all discussed in the manuscript.

   We addressed this point, also raised by the other reviewers, and as described above, removed the term "germ track" from the title and the Results. Again: we wanted to point out that at any time, AGO5 or AGO9 or both are present - though not exclusively - in cells of the germ track. We have now limited usage of the terms "germ track and germline" in connection with AGO5 and AGO9 to the Introduction and the Discussion.

2) An exciting finding is that genes expressed specifically in stem cells form 2 groups; it would have been exciting to see, for example, promoter-reporter expression of two candidates of each group. Similar, authors show that transposons are highly expressed in AGO5/9-expressing cells. Reporters confirming this enriched expression pattern would have been very helpful.

   Please see our detailed answer to Reviewer #2' major comments and our extended analysis of layer- specific gene expression described above.

3) Authors compare the AGO5/9 cargo and the transcriptome in stem cells/meristems, but not in the germline/gametes. DNA methylation was also studied in sperm cells. I think sperm cells should have been included also in cargo and transcriptomic studies.

   Please note that we provide transcriptome data from sperm cells, including the exclusive transcripts for AGO5 and AGO9 among the whole AGO family in Supplemental Figure S21a,b,c. We acknowledge the reviewer's suggestion to include AGO5 and AGO9 cargo data from sperm cells, and we agree that it could be a valuable addition. However, such experiments are not trivial and would extend beyond the scope of our current work.

MINOR

4) I am not really sure, whether the data shown in Suppl. Fig. 5 (role of AGO5 and AGO9 in restricting the number of MMC precursors in ovule primordia) is relevant for the manuscript and should be removed.

Please understand that we decided not to remove these data, even though the *ago9* mutant phenotype differs quantitatively from a previous report (Olmedo-Monfil et al. 2010). Here, the data are crucial to demonstrate that our translational reporter is functional, as it can complement the ago5/ago9 double mutant phenotype.

5) Lines 110-111: authors write that 'At the onset of flowering, the AGO9 signal seemingly migrates from the inflorescence meristem into floral meristems.' I don't think that they show migration - either they show indeed that AGO9 migrates or they change their wording.

We have changed the text from "seemingly migrates" to "relocates" (now line 136).

6) Lines 151-161; authors partly refer to Fig. 3 images, but mean Fig. 2 images.

We have checked and corrected all references to Figures and Supplements.

7) Legend of Fig. 3: it is not possible for a non-expert to understand the figure after reading the short figure legend.

We apologize for the cryptic legend and expanded the text accordingly (Lines 721-736).

TPC2023-RA-00052R1 2nd Editorial decision – *acceptance pending* Sept. 14, 2023

We are pleased to inform you that your paper entitled "Two AGO proteins loaded with transposon-derived sRNA are associated with the reproductive cell lineage throughout development in Arabidopsis" has been accepted for publication in The Plant Cell, pending a final editorial review by a science editor.

Final acceptance from Science Editor Nov. 23, 2023
